# Folic Acid Attenuates Glial Activation in Neonatal Mice and Improves Adult Mood Disorders Through Epigenetic Regulation

**DOI:** 10.3389/fphar.2022.818423

**Published:** 2022-02-07

**Authors:** Tiantian Zhao, Dong Wu, Jingyi Du, Guowei Liu, Guangyu Ji, Zixiao Wang, Fan Peng, Lajie Man, Wenjuan Zhou, Aijun Hao

**Affiliations:** ^1^ Key Laboratory for Experimental Teratology of Ministry of Education, Shandong Key Laboratory of Mental Disorders, Key Laboratory of Birth Regulation and Control Technology of National Health Commission of China, Department of Anatomy and Histoembryology, School of Basic Medical Sciences, Cheeloo College of Medicine, Shandong University, Jinan, China; ^2^ Department of Neurosurgery, Cheeloo College of Medicine, Qilu Hospital of Shandong University and Institute of Brain and Brain-Inspired Science, Shandong University, Jinan, China

**Keywords:** folic acid, postnatal immune infection, glial cells, depression, anxiety, epigenetics

## Abstract

Growing evidence indicates that postnatal immune activation (PIA) can adversely increase the lifetime risk for several neuropsychiatric disorders, including anxiety and depression, which involve the activation of glial cells and early neural developmental events. Several glia-targeted agents are required to protect neonates. Folic acid (FA), a clinical medication used during pregnancy, has been reported to have neuroprotective properties. However, the effects and mechanisms of FA in PIA-induced neonatal encephalitis and mood disorders remain unclear. Here, we investigated the roles of FA in a mouse model of PIA, and found that FA treatment improved depressive- and anxiety-like behaviors in adults, accompanied by a decrease in the number of activated microglia and astrocytes, as well as a reduction in the inflammatory response in the cortex and hippocampus of neonatal mice. Furthermore, we offer new evidence describing the functional differences in FA between microglia and astrocytes. Our data show that epigenetic regulation plays an essential role in FA-treated glial cells following PIA stimulation. In astrocytes, FA promoted the expression of IL-10 by decreasing the level of EZH2-mediated H3K27me3 at its promoter, whereas FA promoted the expression of IL-13 by reducing the promoter binding of H3K9me3 mediated by KDM4A in microglia. Importantly, FA specifically regulated the expression level of BDNF in astrocytes through H3K27me3. Overall, our data supported that FA may be an effective treatment for reducing mood disorders induced by PIA, and we also demonstrated significant functional differences in FA between the two cell types following PIA stimulation.

## Highlights

1) FA improves postnatal immune activation (PIA)-induced depression and anxiety by inhibiting glial cell activation.
2) FA exerts anti-inflammatory effects on microglia and astrocytes through different mechanisms.
3) FA regulates gene expression *via* different epigenetic mechanisms in glial cells.

## 1 Introduction

Postnatal immune activation (PIA), especially neuroinflammation due to infections during the early stages of brain development, including the prenatal and early postnatal periods, aggravates neuropsychiatric disorders in adulthood, such as depression and anxiety (Hagberg et al., 2012; Estes and McAllister, 2016; Claypoole et al., 2017; Claypoole et al., 2017; Schelder-Marzzani et al., 2019; Cao et al., 2021). The ontogeny of these disorders is poorly understood, but clinical research has identified early life immune activation as a significant risk factor for the scaffolding of lifelong social deficits (Brown et al., 2004; Khandaker et al., 2014; Metcalf et al., 2017). Intraperitoneal injection of LPS into neonatal mice mimics bacterial infection, providing a PIA model to observe long-lasting behavioral changes and investigate human psychiatric development after postnatal infection (Carlezon et al., 2019; Liang et al., 2019). LPS exposure in juvenile mice can significantly induce the formation of depressive phenotypes in adult mice (Liang et al., 2019) and the reactive activation of astrocytes and microglia (Norden et al., 2016; Dos-Santos-Pereira et al., 2020). Although the mechanisms underlying neurological deficits in PIA-induced sickness behaviors remain unclear, it has been proposed that inflammation-induced chronic cytokine upregulation by activated glial cells plays an important role (O'Loughlin et al., 2017; Liang et al., 2019). Recent studies have also reported that the occurrence of depressive symptoms is related to the abnormal function of astrocytes and microglia (Sanacora and Banasr, 2013; Yirmiya et al., 2015; Wang et al., 2017). Therefore, anti-inflammatory agents targeting glial cells may have potential protective effects against PIA-induced neuropsychiatric disorders. However, treatment options remain very limited, especially in newborns. It is urgent to find safe and effective drugs for newborns.

Folic acid (FA), a water-soluble vitamin, plays an important role in preventing neural tube malformation and various neonatal diseases. FA supplementation can effectively reduce neonatal mortality of NTDs (Blencowe et al., 2010). In the neonatal hypoxia-ischemia animal model, FA can prevent anxiety-like behaviors and aversive memory deficits (Carletti et al., 2012). FA deficiency decreased cell proliferation and inhibited neuronal differentiation from rhesus monkey embryonic stem cells (Chen et al., 2012). However, it has not been reported whether FA can be used to treat neonatal encephalitis and PIA-induced neuropsychiatric disorders. In this study, we found that intraperitoneal injection of FA in newborn mice significantly inhibited LPS-induced activation of glial cells and improved depressive- and anxiety-like behaviors induced by PIA in adulthood. However, it is not completely clear whether FA can regulate glial cell reactions and function in the PIA model, and its mechanisms in PIA-induced depression and anxiety still need to be elucidated. FA, as a methyl donor for carbon metabolism, can regulate gene transcription expression by affecting the methylation of histones, DNA, and RNA (Leung et al., 2013). Epigenetic mechanisms could mediate lasting increases in the risk of depression following exposure to adverse life events (Provenzi et al., 2018; Penner-Goeke and Binder, 2019). Interference with epigenetic modification patterns can alter gene expression, leading to abnormal functions. A previous study showed that FA treatment induces trimethylation of histone H3 lysine 27 (H3K27me3) in neural stem cells in a neural tube defects model (Zhai et al., 2016a). This observation points to the potential interference of FA with epigenetic mechanisms and regulates cell function. In this study, we mainly focused on the effects of FA on epigenetic mechanisms in PIA-induced neuropsychiatric disorders, in addition to inflammation-related signaling pathways, such as NF-κB and MAPK signaling pathways.

In this study, we aimed to investigate the effects and mechanisms of FA on neonatal encephalitis and PIA-induced neuropsychiatric disorders. Our data demonstrated that FA could improve PIA-induced depressive-like and anxiety-like phenotypes in adult mice. Furthermore, FA could provide a potential therapeutic role by ameliorating the excessive activation of glial cells and neuroinflammation in neonatal mice through different regulatory mechanisms.

## 2 Materials and Methods

### 2.1 Animals and Experimental Design

All animal care and experiments followed the National Institutes of Health Guide for the Care and Use of Laboratory Animals and were approved by the Institutional Animal Care and Use Committee of Shandong University. C57BL/6 mouse pups (weighing 3 g) were purchased from the Shandong University Experimental Animal Center. The animals included both males and females. All animals were housed under standard laboratory conditions in ventilated cages with 12 h light: dark cycles in a specific pathogen-free environment.

The PIA model used in this study was based on previous studies with slight modifications (Claypoole et al., 2017; Liang et al., 2019; Hashimoto et al., 2020). The day of birth was defined as postnatal day 0 (P0). From P3 to P5, the PIA group was removed from their dams, weighed, and received a daily subcutaneous injection of 50 μg/kg dose of LPS (*Escherichia coli* strain O111:B4; Sigma, United States, #L2630; dissolved in 0.9% saline), while the control group received a daily subcutaneous injection of 0.9% saline. FA (Sigma, United States, #F7876) was dissolved in 0.9% saline, and the PIA+FA group received daily subcutaneous injections of 4 mg/kg FA. Previous studies have shown that drug pretreatment offers better protection against brain injury and mood disorders induced by LPS (Arioz et al., 2019; Ali et al., 2020b; Li et al., 2021b). Therefore, FA was administered daily *via* intraperitoneal injection 2 h before LPS injection. In our preliminary experiment, the PIA+FA group was administered different doses of FA (1, 2, 4, and 5 mg/kg) to determine the optimal therapeutic dose. Our results showed that 4 mg/kg FA appeared to be the most optimally effective concentration (Supplementary Figures S1A–J). Therefore, a dose of 4 mg/kg was used to investigate the effects of FA on PIA-induced mood disorders. We also detected the effect of the sham + FA group on mouse behaviors and found no significant difference compared with the control group (Supplementary Figures S1H–K). Animals were divided into two large subgroups: some animals were euthanized and perfused at P6 after the last treatment for short-term morphological and biochemical assessment, while some animals were brought up for long-term behavioral experiments at P42–P48.

### 2.2 Behavioral Tests

Animal behavioral experiments were performed in the following order: open field test (OFT), elevated plus-maze test (EPM), tail suspension test (TST), and forced swimming test (FST). The mice rested for 24 h after each experiment. During the experiment, the operator was blinded to the treatment groups. Animal behavioral experiments were carried out in a separate darkroom free from unwanted external light and sound. All behavioral experiments were recorded, measured, and analyzed using a tracking system (TopScan 3.0).

#### 2.2.1 Open Field Test

The OFT was conducted similarly to the previous study (Chen et al., 2014). Briefly, mice were acclimated to the experimental room for 1 h and then placed in the center (20 × 20) of a 35 cm × 35 cm × 25 cm chamber. The mice were allowed to freely explore the field for 10 min to observe their locomotor activity. The total travel distance and time spent in the central areas were recorded and analyzed as measures of locomotor activity and anxiety-like behavior. The floor of the open field was cleaned with a 5% ethanol solution between tests to prevent the transmission of olfactory cues (Li et al., 2017). The number of mice in each group was as follows: control, *n* = 8–9; PIA, *n* = 7–8; and PIA+FA, *n* = 6–9.

#### 2.2.2 Elevated Plus-Maze Test

The EPM was conducted as previously described (Samad et al., 2018). The EPM apparatus consists of two open arms and two closed arms with a center platform. The EPM was elevated 50 cm above the floor, and mouse was placed in the center area and allowed to explore the EPM for 6 min freely. The amount of duration spent in open arms (%) and bouts in the open arms (%) were recorded and analyzed as a measure of anxiety-like behavior with a tracking system. The floor of the arms was cleaned with 5% ethanol solution between tests to prevent transmission of olfactory cues. The number of mice in each group was as follows: control, *n* = 6–7; PIA, *n* = 6; and PIA+FA, *n* = 6–7.

#### 2.2.3 Tail Suspension Test

The depressive-like behavior of the mice was analyzed with TST as previously described, with slight modifications (Gould et al., 2008). For the test, the mice were suspended individually by an adhesive tape. The tape was placed 1 cm from the tip of the tail. The test procedures were videotaped for 6 min and analyzed by a blind observer. Mice that climbed their tails were removed from the experimental analysis. Mobility was defined as the movement of the hind legs. The number of mice in each group was as follows: control, *n* = 7; PIA, *n* = 6; and PIA+FA, *n* = 9.

#### 2.2.4 Forced Swimming Test

The FST was another widely used behavioral paradigm to measure depressive-like behavior (Shi et al., 2012; Zheng et al., 2016). The experiment was conducted in an open glass cylinder (25 cm height, 18 cm diameter) filled up to 18 cm with water at 22°C–25°C as previously described. Each mouse was forced to swim for 6 min. Water in the cylinder was changed after each animal to prevent behavioral alteration among animals. The observers were blind to the treatment design. Mice were considered immobile when they stopped swimming and stayed motionless, making only necessary movements to keep their heads above the water. Immobility time during the last 5 min of the 6-min session was recorded. The number of mice in each group was as follows: control, *n* = 6; PIA, *n* = 7; and PIA+FA, *n* = 6.

### 2.3 Production of Microglial and Astroglial Cultures

#### 2.3.1 Coating Procedure With Poly-L-Lysine

Poly-L-lysine (PLL; #P3143, Sigma, United States) was used to coat culture flasks to culture adherent growth primary microglia and astrocytes as previously described (Sparapani et al., 1997). PLL was diluted to 0.1 mg/ml in phosphate of saline solution (PH 7.0–7.4). After at least 1 h of incubation, at 37°C, PLL was discarded, and the culture flasks were washed three times with sterile double-steamed water. The surface was allowed to dry. Ultraviolet radiation was then used to disinfect the culture flasks for 30 min before applying the culture medium and cell seeding.

#### 2.3.2 Microglia and Astrocyte Isolation

Primary astrocytes and microglia from the cerebral cortex and corpus callosum were isolated from mice pups (3-day-old) and prepared for *in vitro* experiments. These glial cells were placed in a 75-cm^2^ flask (Beaver) and cultured in Dulbecco’s modified Eagle’s medium–high-glucose medium (Gibco, United States; #11965092) containing 10% fetal bovine serum (Gibco, United States; #10099141C) and penicillin–streptomycin–amphotericin cocktail (Macgene, Beijing, China; #CC033). The flasks were then placed in a 5% CO_2_ incubator at 37°C. The medium was changed every 3 days. After approximately 10–12 days, mixed primary glial cells were shaken at 200 rpm and 37°C for 4 h to harvest the primary microglial cells. The majority of the other cells were primary astrocytes.

### 2.4 Immunofluorescence

Immunofluorescence (IF) was performed as previously described (Zhou et al., 2019). Briefly, the pups were decapitated, and the brain tissues were removed and post-fixed in 4% paraformaldehyde for 24 h, and then transferred to 30% sucrose in PBS until the brain sunk to the bottom. Sagittal brain sections (30 μm) were obtained using a frozen microtome. Floating sections were permeabilized with 0.1% Triton X-100 in PBS, incubated in 0.3% H_2_O_2_/50% methanol in PBS, mounted on slides, and dried. The slides were blocked with 10% normal goat or donkey serum containing 0.5% Triton X-100 in PBS at room temperature for 3 h and then incubated with primary antibodies (diluted in PBS) overnight at 4°C. The dilutions for the primary antibodies were as follows: anti-GFAP (1:200), anti-Iba1 (1:200), anti-H3K27me3 (1:200), and anti-H3K9me3 (1:100). After washing with PBS, the cells and sections were incubated with secondary antibodies conjugated to Alexa Fluor 488 or Alexa Fluor 594 for 1 h at room temperature, and then washed three times with PBS. Before fluorescent photography, cells and brain sections were stained with 2 µg/ml 4, 6-diamidino-2-phenylindole (DAPI). The images were captured using a fluorescence microscope (Olympus BX51; Olympus, Shinjuku, Japan). Details of antibodies are listed in Table 1 (*n* = 4 per group).

**TABLE 1:** Antibodies used for immunohistochemistry, Western blotting, and ChIP.

Antibodies	Catalog number	Company
P-NF-κB	3033T	Cell signaling
NF-κB	8242S	Cell signaling
p-IκB	2859T	Cell signaling
H3K27me3	9733S	Cell signaling
H3K9me3	5327S	Cell signaling
H3K4me3	9751S	Cell signaling
Acetylated-lysine	9441S	Cell signaling
iNOS	13120S	Cell signaling
GFAP	3670P	Cell signaling
H3	9715S	Cell signaling
P-P38	AM063-1	Beyotime
EZH2	Ab191080	Abcam
Iba1	Ab178680	Abcam
JNK	AJ518	Beyotime
P-JNK	9251S	Abcam
ERK1/2	AF1051	Beyotime
P-ERK1/2	CY5277	Abways
TNFα	bs-2081R	Bioss
IL-1β	AF5103	Affbiotech
IL-10	DF6894	Affbiotech
KDM4A	24943-1-AP	Proteintech

### 2.5 Western Blotting

The culture medium was removed from the culture dish, and the cells were washed three times with pre-cooled PBS. The cells were centrifuged at 800 rpm for 10 min at 4°C, lysed with RIPA buffer, and placed on ice for 40 min. Then, it was centrifuged at 13,000 rpm for 20 min at 4°C, and the supernatant was collected. The protein concentration of experimental samples was determined using a BCA Assay Kit (Beyotime, Shanghai, China). Three micrograms of the protein sample were loaded and separated using 10% sodium dodecyl sulfate-polyacrylamide gels. Next, the protein samples were embedded in gels and transferred to polyvinylidene fluoride membranes using a semi-dry electrophoretic transfer tank (Bio-Rad, United States). The membranes were washed three times with 1x TBST and incubated with 5% skim milk for 2 h at room temperature. The membranes were then incubated with the primary antibodies listed in Table 1 overnight on a shaker at 4°C. After recovering the primary antibody, the membranes were washed three times with 1X TBST and incubated with a horseradish peroxidase–conjugated secondary antibody for 1 h. The proteins were detected using a chemical exposure meter (Millipore, United States) and captured on the films. The stripe intensity was quantified using ImageJ software (ImageJ 1.46r). All experiments were repeated for a minimum of triplicates. The details of the antibodies used are listed in Table 1 (*n* = 4 per group).

### 2.6 RNA Extraction and Quantitative Real-Time Polymerase Chain Reaction

Total RNA was isolated from the cells using TRIzol reagent (Invitrogen, United States) according to the manufacturer’s instructions. The RNA concentration was determined using a spectrophotometer (Bio-Rad, United States) at 260 nm. Complementary DNA (cDNA) was generated from 1 μg of total RNA using the Primescript RT reagent Kit with gDNA Eraser (TOYOBO, Japan) according to the manufacturer’s instructions. The mRNA levels of various genes were quantified by qPCR using the SYBR Green QuantiTect RT-PCR Kit (TOYOBO, Japan) and specific primers. Primer sequences for the target genes are shown in Table 2. Actin and 18S were used as the reference gene. qPCR was conducted under the following conditions for 39 cycles: pre-degeneration at 95°C for 10 min, denaturation at 95°C for 15s, and annealing at 60°C for 1 min. Data were analyzed using the relative standard curve method, according to the manufacturer’s protocol (*n* = 4 per group).

**TABLE 2:** Primer sequences used for qRT-PCR experiments.

qRT-PCR primer sequence (mouse)		
Iba-1	Forward	ATG​CTG​GAG​AAA​CTT​GGG​GT
	Reverse	CCA​GTT​GGC​CTC​TTG​TGT​TC
TNF-α	Forward	TCT​CAT​TCC​TGC​TTG​TGG​C
	Reverse	CAC​TTG​GTG​GTT​TGC​TAC​G
IL-1β	Forward	AGCATCCAGCTTCAAATC
	Reverse	CTTCTCCACAGCCACAAT
iNOS	Forward	GGG​CTG​TCA​CGG​AGA​TCA​ATG
	Reverse	GCC​CGG​TAC​TCA​TTC​TGC​ATG
IL-10	Forward	ATA​ACT​GCA​CCC​ACT​TCC​CA
	Reverse	GGG​CAT​CAC​TTC​TAC​CAG​GT
GFAP	Forward	AACAACCTGGCTGCGTAT
	Reverse	ACT​GCC​TCG​TAT​TGA​GTG​C
18S	Forward	GTA​ACC​CGT​TGA​ACC​CCA​TT
	Reverse	CCA​TCC​AAT​CGG​TAG​TAG​CG
KDM6A	Forward	TAC​CAG​GCC​TCC​TCA​TTC​CA
	Reverse	ACA​ACT​CTC​ACG​AAG​GCA​GG
KDM6B	Forward	TCG​CGG​TAC​ATG​AGC​ACT​AT
	Reverse	TAG​CCT​GCA​CCC​AAT​GTA​CA
BDNF	Forward	TGT​GAT​CCC​GGA​GAG​CAG​AG
	Reverse	ACC​CAG​TAT​ACC​AAC​CCG​GA
EZH2	Forward	TCT​GGA​GGG​AGC​TAA​GGA​GT
	Reverse	GTC​CCT​GCT​TCT​CTG​TCA​CT
KMT1A	Forward	TGG​TTA​AGT​GGC​GTG​GGT​ATC​C
	Reverse	GGC​TAG​GTT​TGG​GTC​CAG​ATG​C
KMT1B	Forward	GGC​TGT​GGT​TGG​GGT​GTA​AA
	Reverse	AGC​TGC​ATC​CAC​TGT​GAA​CT
KMT1C	Forward	GAT​GTG​ATT​CGT​ATG​CTG​CTG​ACC
	Reverse	CTC​CCT​GGC​GGC​TAT​GTG​C
KMT1D	Forward	ACG​CTC​GGT​TCT​ATG​GGA​ATG​TC
	Reverse	ACA​CTC​GCA​CAG​GCA​CAA​GG
KMT1E	Forward	AAC​AAG​TAG​CCA​AGA​AGA​GCA​CAT​C
	Reverse	GTT​TAT​CCC​AAG​TTT​CCC​AGC​AGA​C
KMT8	Forward	GGGACCTCGGAACTCAAC
	Reverse	TGTATCTGCCTGGGACTG
KDM4A	Forward	CTC​TTT​GCC​TGC​CCC​TGG​TC
	Reverse	AAC​ACT​GAC​TCC​TCT​GAA​CTC​CTC
KDM4B	Forward	GGC​ACC​AGT​CCA​CTC​ATC​TCC
	Reverse	CAG​CAT​TCC​GCA​GTC​CAA​GC
KDM4C	Forward	AGA​TGG​ATT​GAC​TAC​GGC​AAG​GTT​G
	Reverse	AGA​TTC​TGG​AGT​GGG​CTT​TGT​ATG​G
KDM4D	Forward	AGT​CTT​GGT​CGT​CGT​CCT​TGT​G
	Reverse	TCT​CTG​CCT​GCT​GGA​AGT​TGC
IL-4	Forward	ATC​GGC​ATT​TTG​AAC​GAG​GTC​ACA
	Reverse	CGA​AGC​ACC​TTG​GAA​GCC​CTA
IL-13	Forward	TGG​CTC​TTG​CTT​GCC​TTG​GTG​G
	Reverse	CCA​TAC​CAT​GCT​GCC​GTT​GCA

### 2.7 Cell Viability Assay

Cell viability was determined using the 3-(4, 5-dimethylthiazol-2-yl)-2, 5-diphenyltetrazolium bromide (MTT) assay (Sigma, United States). Astrocytes were plated in 96-well plates at a density of 8,000 cells/well and supplemented DMEM. After treatment with different concentrations of FA for 24 h, 10 μl of MTT solution (5 mg/ml) was added to each well and incubated at 37°C for 4 h. Then, the medium was discarded, and 200 μl of dimethyl sulfoxide (DMSO; Solarbio, Beijing, China) was added. The absorbance was measured at 490 nm using a multi-well spectrophotometer (Bio-Rad).

### 2.8 Chromatin Immunoprecipitation Assay

Chromatin immunoprecipitation assay (ChIP) was performed using a Simple CHIP (R) Kit (Cell Signaling Technology, United States). Cultured microglia and astrocytes were treated with 1% formaldehyde for 15 min at room time to generate cross-links between histones and DNA. The cells were then washed thrice with PBS and collected in SDS lysis buffer supplemented with protease inhibitors. After sonication, the chromatin in each cell lysate sample was sheared into 200–500  bp fragments. The supernatants were immunoprecipitated with a specific antibody, anti-H3K9me3, anti-H3K27me3, or a control antibody (anti-IgG) overnight at 4°C. The supernatants were then subjected to washing, elution, and cross-link reversal, following the manufacturer’s instructions. Purified genomic DNA from the supernatants was analyzed by real-time PCR. The primers used are listed in Table 3 (*n* = 3 per group).

**TABLE 3:** Primer sequences used for ChIP-qPCR experiments

BDNF	Forward	GGA​GGA​AGC​GAG​TGT​GTG​AGT​C
	Reverse	AAA​CCA​GGG​GAG​AAA​GAT​TTG
IL-10	Forward	GGA​GGA​GGA​GCC​TGA​ATA​AC
	Reverse	CTG​TTC​TTG​GTC​CCC​CTT​TT
IL-13	Forward	TGC​CCT​GTC​CTC​CAG​ATT​GA
	Reverse	GAA​ATC​TGC​CTC​CGT​CCC​TT

### 2.9 Statistical Analysis

Experimental values expressed as mean ± SEM were derived from at least three independent experiments. Data statistical significance was calculated by one-way ANOVA, followed by the Bonferroni *post hoc* test using GraphPad Prism version 8 for Windows (GraphPad Software, La Jolla, CA).

## 3 Results

### 3.1 Folic Acid Treatment Ameliorated Depressive- and Anxiety-like Behaviors Induced by Postnatal Immune Activation in Adult Mice

**FIGURE 1:**
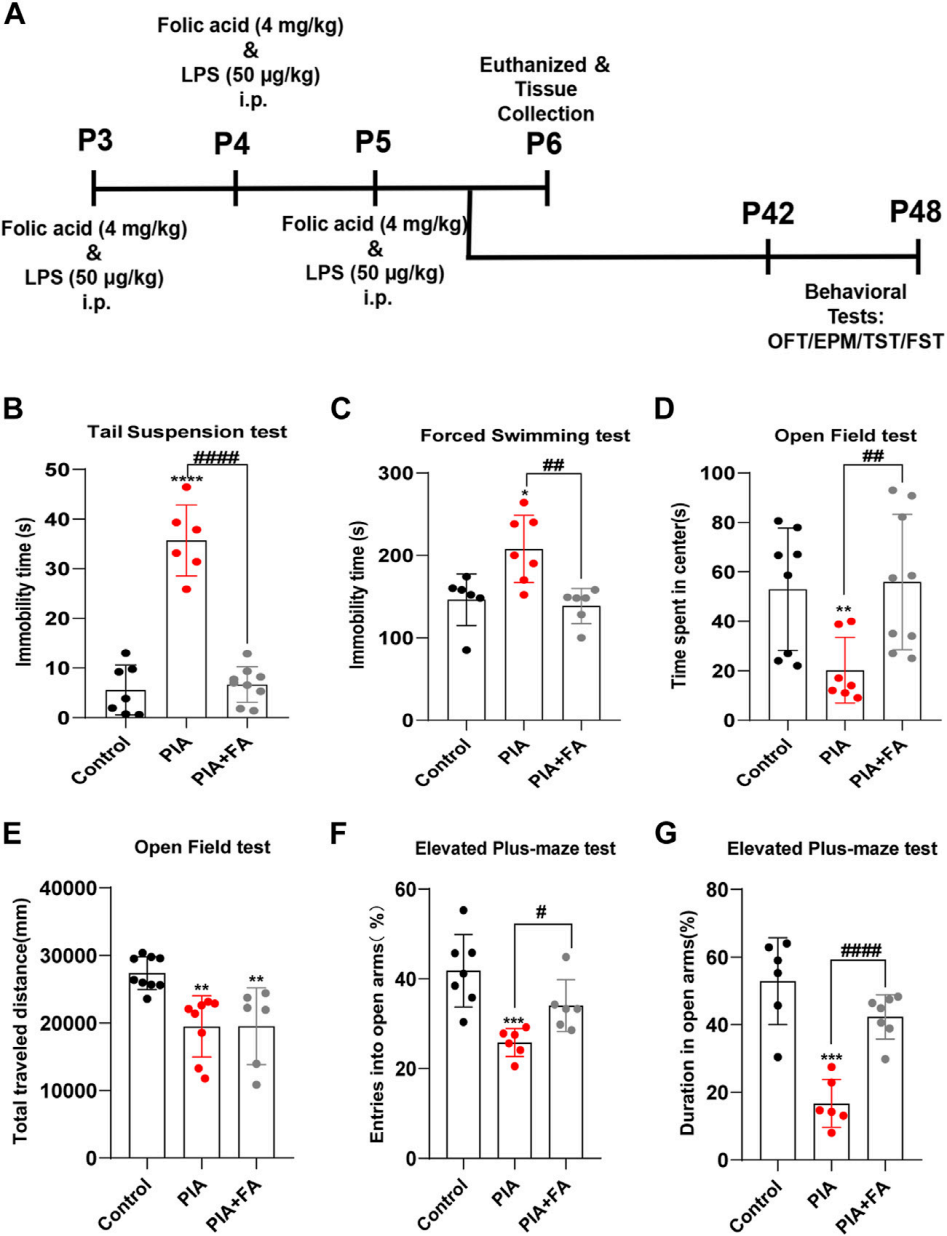
FA improved PIA-induced depressive-like and anxiety-like behaviors in adults. **(A)** Schematic representation of the experimental design for the PIA model. **(B,C)** Immobility time in TST and FST. FA improved depressive-like behaviors in PIA-induced mice. **(D)** Time spent in the center of OFT. **(E)** Traveling distances in OFT were used to examine motor ability. **(F)** The percentage of entries into the open arms in EPM. **(G)** The percentage of time spent in the open arm in EPM. Anxiety-like behaviors were evaluated after RA treatment in EPM and OFT. Data: mean ± SEM; *p < 0.05, **p < 0.01, ***p < 0.001, ****p < 0.0001 versus the control group; ^#^ *p* < 0.05, ^##^ *p* < 0.01, ^####^ *p* < 0.0001 versus the PIA group; *n* = 6–9 per group (one-way ANOVA).

To examine the effects of FA on depressive- and anxiety-like behaviors induced by PIA, we performed different behavioral tests, including OFT, EPM, TST, and FST (Figure 1A). Compared to the control group, mice in the PIA group showed severe behavioral deficits in adults. FA treatment significantly improved depressive- and anxiety-like behaviors following PIA exposure. For depressive-like behaviors, FA treatment significantly reduced the immobility time in the TST (*p* < 0.0001) and FST (*p* = 0.0034) compared with the PIA group (Figures 1B,C). Regarding anxiety-like behaviors, the decrease of time spent in the center induced by PIA exposure was reversed by FA treatment in the OFT (*p* = 0.0071) (Figure 1D). However, there was no significant difference in the total distance traveled between the PIA and PIA+FA treatment groups (*p* = 0.9833) (Figure 1E). In EPM, the percentage of entries into the open arms (*p* = 0.0119) and the percentage of time spent in the open arms (*p* < 0.0001) were significantly increased by FA treatment compared to the PIA group (Figures 1F,G). Overall, these results indicate that FA can improve depressive- and anxiety-like behaviors induced by PIA in adults.

### 3.2 Folic Acid Treatment Inhibited Postnatal Immune Activation–Induced Microglia and Astrocyte Activation as Well as Inflammatory Responses in Neonatal Mice

**FIGURE 2:**
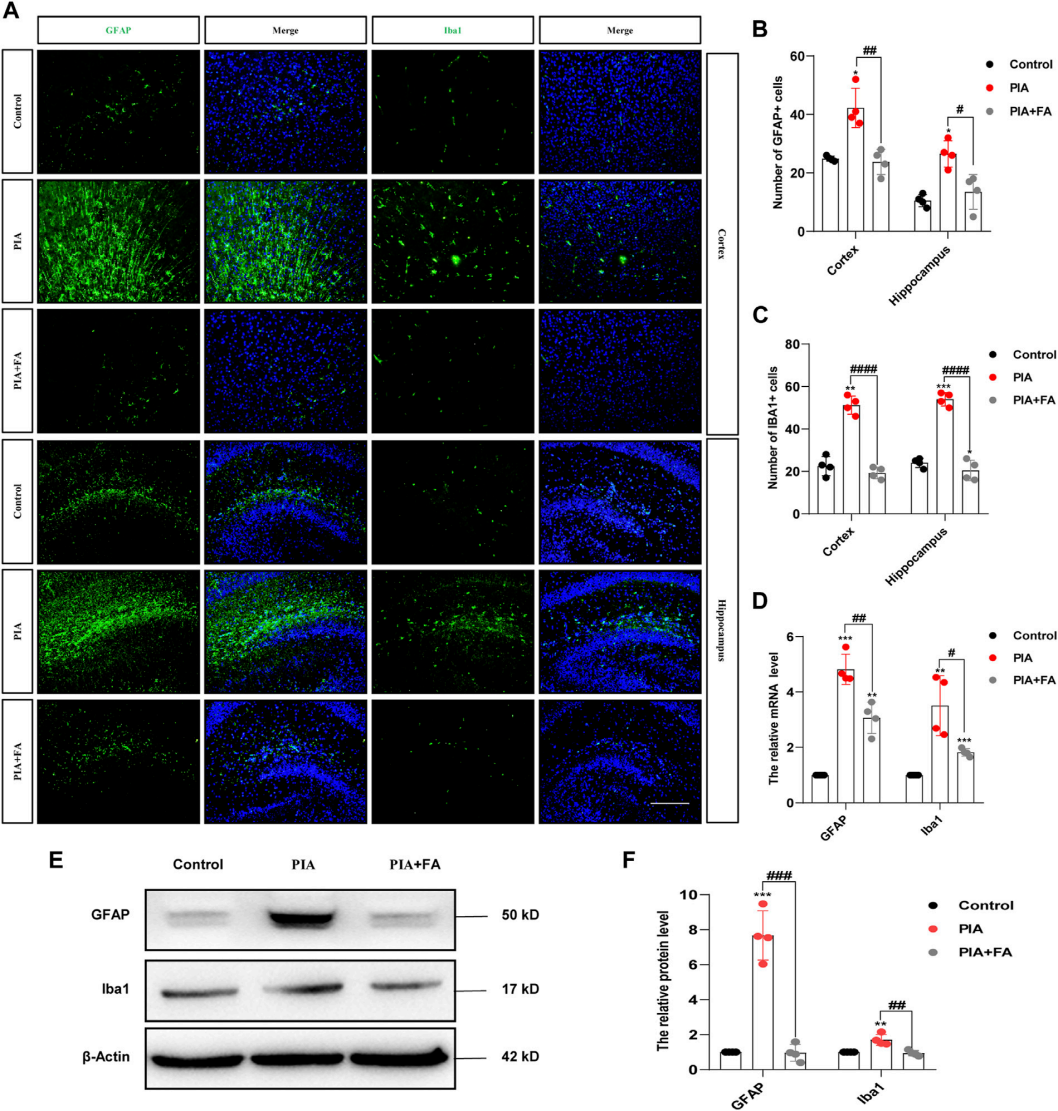
FA inhibited the activation of astrocytes and microglia in the brain of neonatal mice. **(A)** Cortex and hippocampal sections were stained with GFAP (the astrocytes marker) or Iba1 (the microglia marker) (green), and nuclei were stained with DAPI to localize and assess the activation levels of astrocytes and microglia. **(B,C)** Quantification of the GFAP-positive cells and Iba1-positive cells. **(D)** Quantification of the mRNA levels of GFAP and Iba1 in the neonatal mice brain. **(E,F)** Representative images and quantification of Western blotting analysis of GFAP and Iba1 expression in the neonatal mouse brain (scale bar: 50 μm). Data: mean ± SEM; *p < 0.05, **p < 0.01, ***p < 0.001 versus the control group; ^#^ *p* < 0.05, ^##^ *p* < 0.01, ^###^ *p* < 0.001, ^####^ *p* < 0.0001 versus the PIA group; *n* = 4 per group (one-way ANOVA).

Microglia and astrocytes are two important types of glial cells in the central nervous system (CNS) that perform a vast number of immune-related duties in response to various CNS injuries (Karve et al., 2016; Saavedra et al., 2021). In this study, we further determined whether the anti-depressant and anti-anxiety effects of FA were mediated by the inhibition of glial activation and inflammatory responses. Immunofluorescence staining for GFAP (a marker for astrocytes) and Iba1 (a marker for microglia) in the cortex and hippocampus sections was performed to evaluate the effects of FA on glial cell activation *in vivo*. The results showed that GFAP-positive (GFAP^+^) cells and Iba1-positive (Iba1^+^) cells were increased in the cortex and hippocampus of neonatal mice in the PIA group. However, FA treatment reduced the number of activated astrocytes (*p* = 0.0036; *p* = 0.0129) and microglia (*p* < 0.0001; *p* < 0.0001) compared to that in the PIA group (Figures 2A–C). Additionally, Western blotting and qPCR showed that FA treatment significantly reduced the protein and mRNA levels of GFAP (*p* = 0.0001; *p* = 0.0043) and Iba1 (*p* = 0.0048; *p* = 0.0213) compared with those in the PIA group (Figures 2D–F).

**FIGURE 3:**
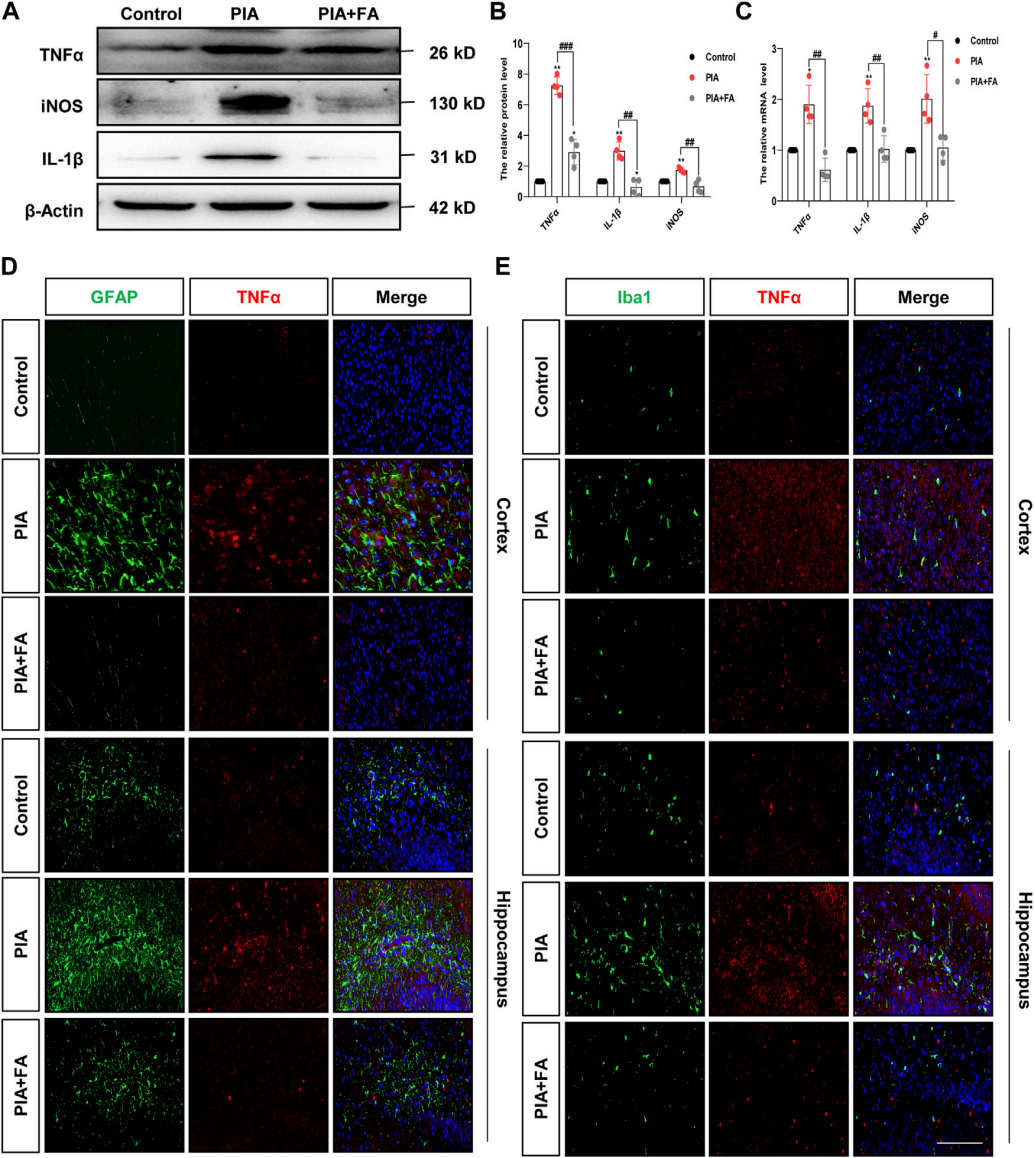
FA reduced the expression of pro-inflammatory mediators in astrocytes and microglia in the PIA model. **(A,B)** Representative images and quantification of Western blotting analysis of pro-inflammatory mediator TNFα, iNOS, and IL-1β protein level in the brain of neonatal mice. **(C)** Quantification of the mRNA levels of pro-inflammatory mediators in the brain of neonatal mice. **(D)** Cortex sections were stained for GFAP (green) and TNFα (red). Nuclei were stained with DAPI (blue). Increased TNFα and GFAP intensity by PIA induction was lowered with FA treatment. **(E)** Cortex sections were stained for Iba1 (green) as the microglia marker and TNFα (red). Nuclei were stained with DAPI (blue). Increased TNFα and Iba1 intensity by PIA induction was lowered with FA treatment (scale bar: 20 μm). Data: mean ± SEM; *p < 0.05, **p < 0.01 versus the control group; ^#^ *p* < 0.05, ^##^ *p* < 0.01, ^###^ *p* < 0.001 versus the PIA group; *n* = 4 per group (one-way ANOVA).

Cytokine induction by glial cells, including IL-1β, TNFα, and iNOS, helps propagate these immune-derived signals and mediate physiological and behavioral responses (Singhal and Baune, 2017; Wang et al., 2019; Lan et al., 2011). The results showed that FA treatment attenuated the induction of IL-1β (*p* = 0.0010; *p* = 0.0074), TNFα (*p* = 0.0001; *p* = 0.0012), and iNOS (*p* = 0.0025; *p* = 0.0118) at the protein and mRNA levels by PIA (Figures 3A–C). Furthermore, immunofluorescence staining of TNFα/GFAP or TNFα/Iba1 further confirmed that FA treatment inhibited the release of pro-inflammatory factors and glial cell activation in the brains of PIA mice (Figures 3D,E). However, the effect of FA in the PIA model was inhibited by using FA antagonist, pyrimethamine. Pyrimethamine is a dihydrofolate reductase (DHFR) inhibitor, which leads to folate deficiency by inhibiting DHFR (Raynaud and Horvàth, 1994; Lambie and Johnson, 1985). The results showed that pyrimidine treatment (PIA+FA+PR group) promoted the inflammatory factor release of IL-1β (*p* = 0.0009) and TNFα (*p* = 0.0028), and the expression of GFAP (*p* = 0.0067) and Iba1 (*p* = 0.0067), as well as the reduction of the ratio of BCL2 to BAX (*p* = 0.0036) compared with the PIA+FA group (Supplementary Figure S2). Together, these results indicate that FA could inhibit the activation of microglia and astrocytes, and exert anti-inflammatory effects in PIA-induced depressive- and anxiety-like behaviors.

**FIGURE 4:**
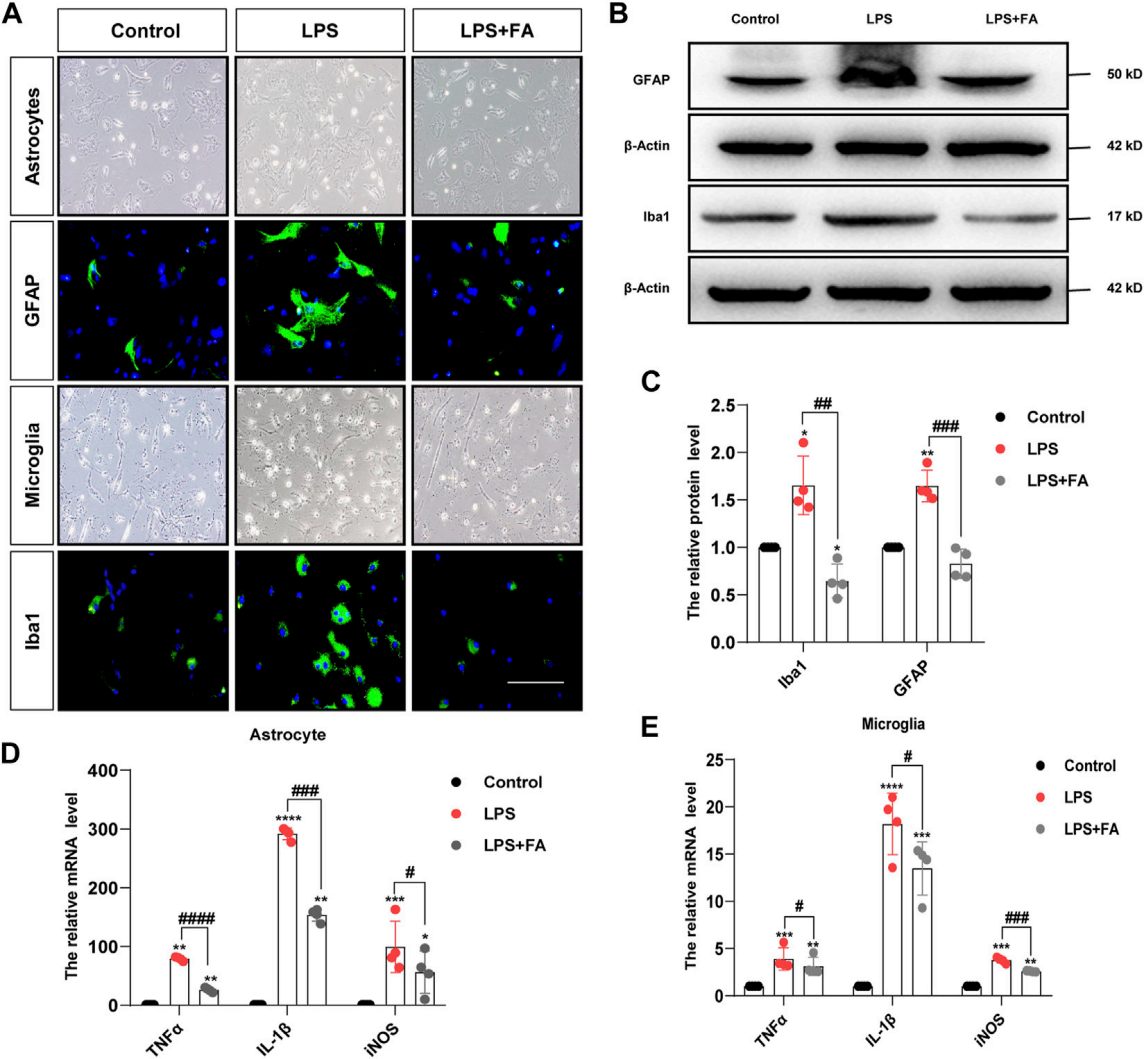
FA inhibited astrocytes and microglia activation induced by LPS *in vitro*. **(A)** Primary astrocytes and microglia were treated with LPS for 24 h, and GFAP and Iba1 were stained (green). **(B,C)** Representative images and quantification of Western blotting analysis of the GFAP and Iba1 expression in primary astrocytes and microglia. **(D,E)** Quantification of the mRNA levels of pro-inflammatory mediator TNFα, IL-1β, and iNOS in astrocytes and microglia (scale bar: 20 μm). Data: mean ± SEM; *p < 0.05, **p < 0.01, ***p < 0.001, ****p < 0.0001 versus the control group; ^#^ *p* < 0.05, ^##^ *p* < 0.01, ^###^ *p* < 0.001, ^####^ *p* < 0.0001 versus the PIA group; *n* = 4 per group (one-way ANOVA).

We further verified the effects of FA on astrocyte and microglial activation, as well as inflammatory responses *in vitro*. Non-toxic doses of FA were determined using the MTT assay. Among these doses, we selected 50 μg/ml as the optimal concentration for primary astrocytes (Supplementary Figure 3A). According to a previous study, the dose of FA for microglia was also 50 μg/ml (Cianciulli et al., 2016). Primary microglia and astrocytes were cultured to evaluate the anti-inflammatory effects of FA (Supplementary Figure 3B). As expected, immunofluorescence staining showed that treatment with FA significantly reduced the expression of the phenotypic activation markers Iba1 and GFAP (Figure 4A). Western blotting also revealed the inhibition of GFAP (*p* = 0.0004) and Iba1 (*p* = 0.0013) expression by FA following LPS treatment (Figures 4B,C). Meanwhile, LPS strongly stimulated the mRNA expression levels of pro-inflammatory cytokines TNFα, IL-1β, and iNOS in astrocytes and microglia, while the expression of TNFα (*p* < 0.0001; *p* = 0.0412), IL-1β (*p* = 0.0001; *p* = 0.0239), and iNOS (*p* = 0.0193; *p* = 0.0002) induced by LPS was curtailed efficiently by FA exposure (Figures 4D,E).

### 3.3 Folic Acid Prevents Inflammatory Events in Glial Cells by Interfering With MAPK and NF-κB Signaling Pathways

**FIGURE 5:**
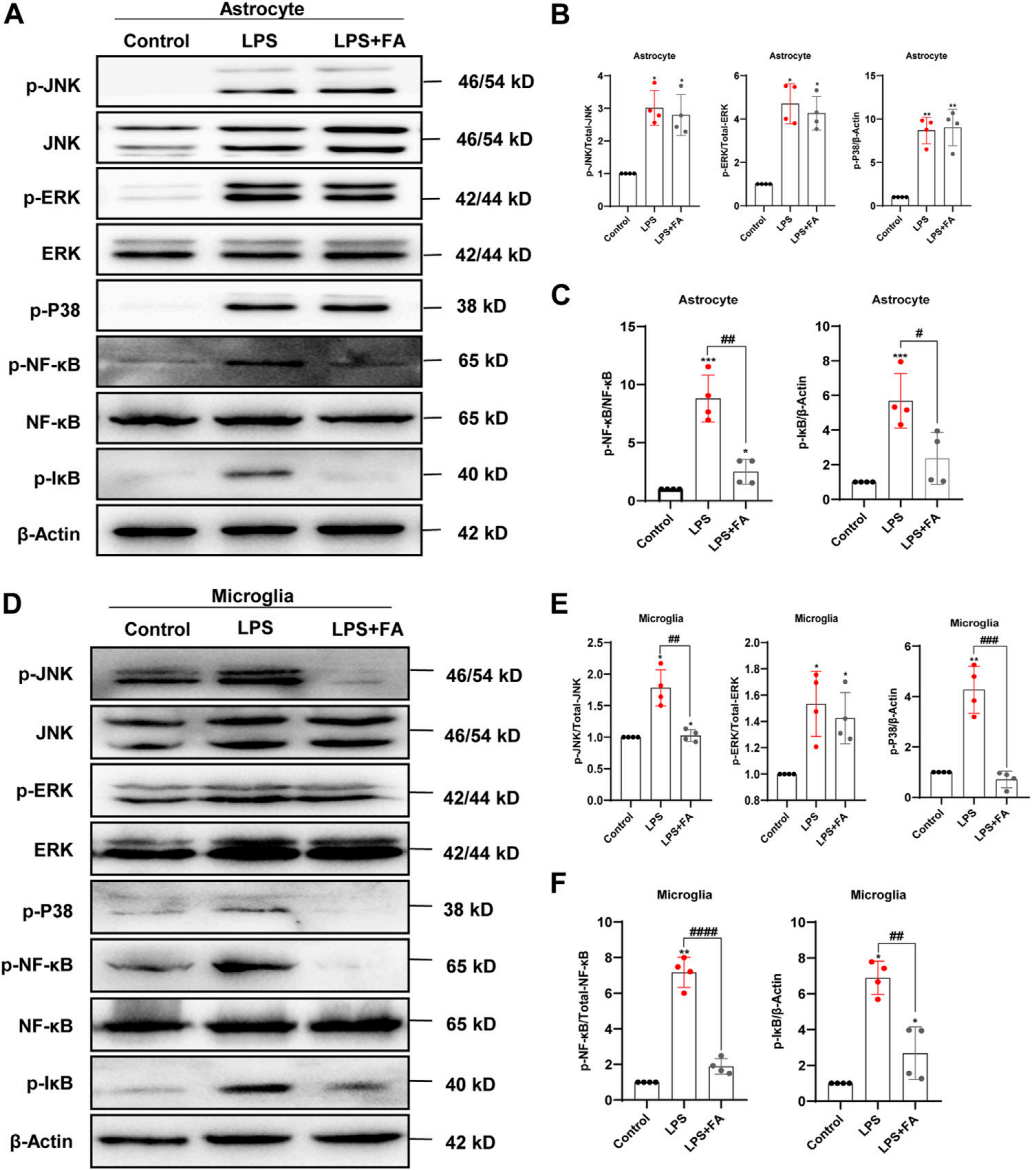
FA regulated the expression levels of key proteins in NF-kB and MAPK signaling pathways in LPS-induced astrocytes and microglia. **(A)** Representative Western blotting images of NF-κB and MAPK signaling protein expressions in the primary astrocytes. **(B,C)** Quantification of the expression levels of proteins in **(A)**. **(D)** Representative Western blotting images of NF-κB and MAPK signaling protein expressions in the primary microglia. **(E,F)** Quantification of the expression levels of proteins in **(D)**. Data: mean ± SEM; *p < 0.05, **p < 0.01, ***p < 0.001 versus the control group; ^#^ *p* < 0.05, ^##^ *p* < 0.01, ^###^ *p* < 0.001, ^####^ *p* < 0.0001 versus the PIA group; *n* = 4 per group (one-way ANOVA).

Previous studies have shown that FA may affect NF-κB and MAPK signaling-dependent events in BV2 cells (Feng et al., 2011). However, it is unclear whether FA plays the same role in astrocytes. To address this point, we monitored the expression levels of the phosphorylated p65 subunit of NF-κB (p-NF-κB), phosphorylated ERK (p-ERK), phosphorylated P38 (p-P38), and phosphorylated JNK (p-JNK) in LPS-treated primary cultured astrocytes and microglia, with or without FA treatment, by Western blotting. The results showed that treatment with FA reduced p-NF-κB (*p* = 0.0015), and p-IκB (*p* = 0.0221) expression compared to the LPS group, but the MAPK signaling pathway was not affected in primary astrocytes (*p* = 0.6179) (Figures 5A–C). In microglia, FA not only reduced the protein levels of p-NF-κB, and p-IκB (*p* < 0.0001; *p* = 0.0029) but also inhibited the activation of p-JNK (*p* = 0.0024) and p-P38 (*p* = 0.0004), which was consistent with a previous study involving BV2 cells (Figures 5D–F). Interestingly, we found that the anti-inflammatory mechanisms of FA in astrocytes and microglia were slightly different.

### 3.4 Folic Acid Regulates Gene Expression in Astrocytes and Microglia Through H3K27me3 and H3K9me3, Respectively

**FIGURE 6:**
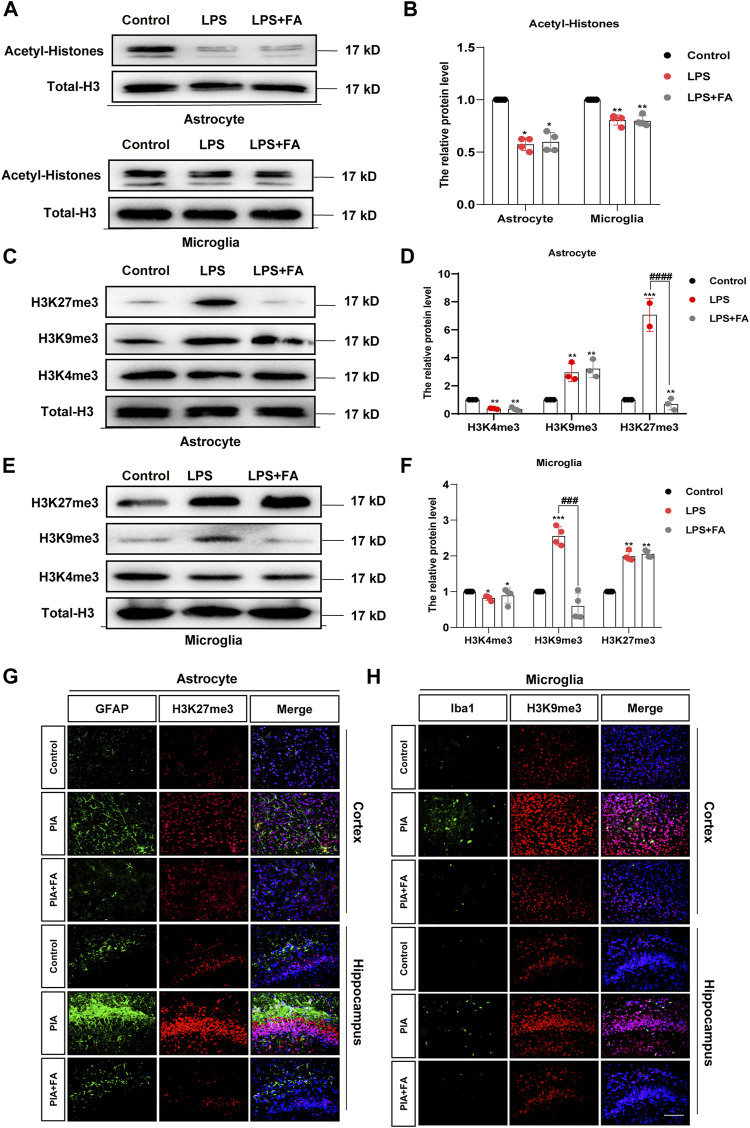
FA regulated astrocyte and microglia activation through H3K27me3 and H3K9me3, respectively **(A,B)** Western blotting analysis of acetyl-H3 protein levels in the primary astrocytes and microglia. FA had no effect on the acetyl-H3 levels. **(C,D)** Western blotting analysis of H3K27me3, H3K9me3, and H3K4me3 protein levels in the primary astrocytes. FA only affected the protein levels of H3K27me3. **(E,F)** Western blotting analysis of H3K27me3, H3K9me3, and H3K4me3 protein levels in the primary microglia. FA only affected the protein levels of H3K9me3. **(G)** Cortex and hippocampus sections were stained with GFAP (green) and H3K27me3 (red). Nuclei were stained with DAPI (blue). Increased H3K27me3 and GFAP intensity by PIA induction was lowered with FA treatment. **(H)** Cortex and hippocampus sections were stained for Iba1 (green) and H3K9me3 (red). Nuclei were stained with DAPI (blue). Increased Iba1 and H3K9me3 intensity by PIA induction was lowered with FA treatment (scale bar: 20 μm). Data: mean ± SEM; *p < 0.05, **p < 0.01, ***p < 0.001 versus the control group; ^##^ *p* < 0.01, ^####^ *p* < 0.0001 versus the PIA group; *n* = 4 per group (one-way ANOVA).

Histone modifications play vital roles in the activation and silencing of gene transcription, and have been proven to be involved in regulating neuroinflammation and neurodevelopmental diseases (Alam et al., 2017; Li et al., 2019). It is well known that histone acetylation can significantly promote gene expression, while the effects of histone methylation on gene expression are relatively more complex. We first detected the effect of FA on the level of histone acetylation and found that FA treatment did not change the LPS-induced decrease in histone acetylation levels in primary astrocytes (*p* = 0.7013) and microglia (*p* = 0.8119) (Figures 6A,B). We further detected changes in several important histone methylation modifications, such as H3K27me3, H3K9me3, and H3K4me3. We found that FA treatment significantly reduced the LPS-induced increase of H3K27me3 in astrocytes (*p* < 0.0001), but the changes in H3K9me3 (*p* = 0.8984) and H3K4me3 (*p* = 0.1519) were not significant (Figures 6C,D). Interestingly, in microglia, Western blotting indicated that LPS treatment increased H3K9me3 levels, which was reversed by FA (*p* = 0.0001), but the changes in H3K27me3 (*p* = 0.4371) and H3K4me3 (*p* = 0.5520) were not significant (Figures 6E–H). Immunofluorescence staining of GFAP/H3K27me3 and Iba1/H3K9me3 further demonstrated that FA regulates histone methylation at different sites in astrocytes and microglia (Figures 6G, H).

**FIGURE 7:**
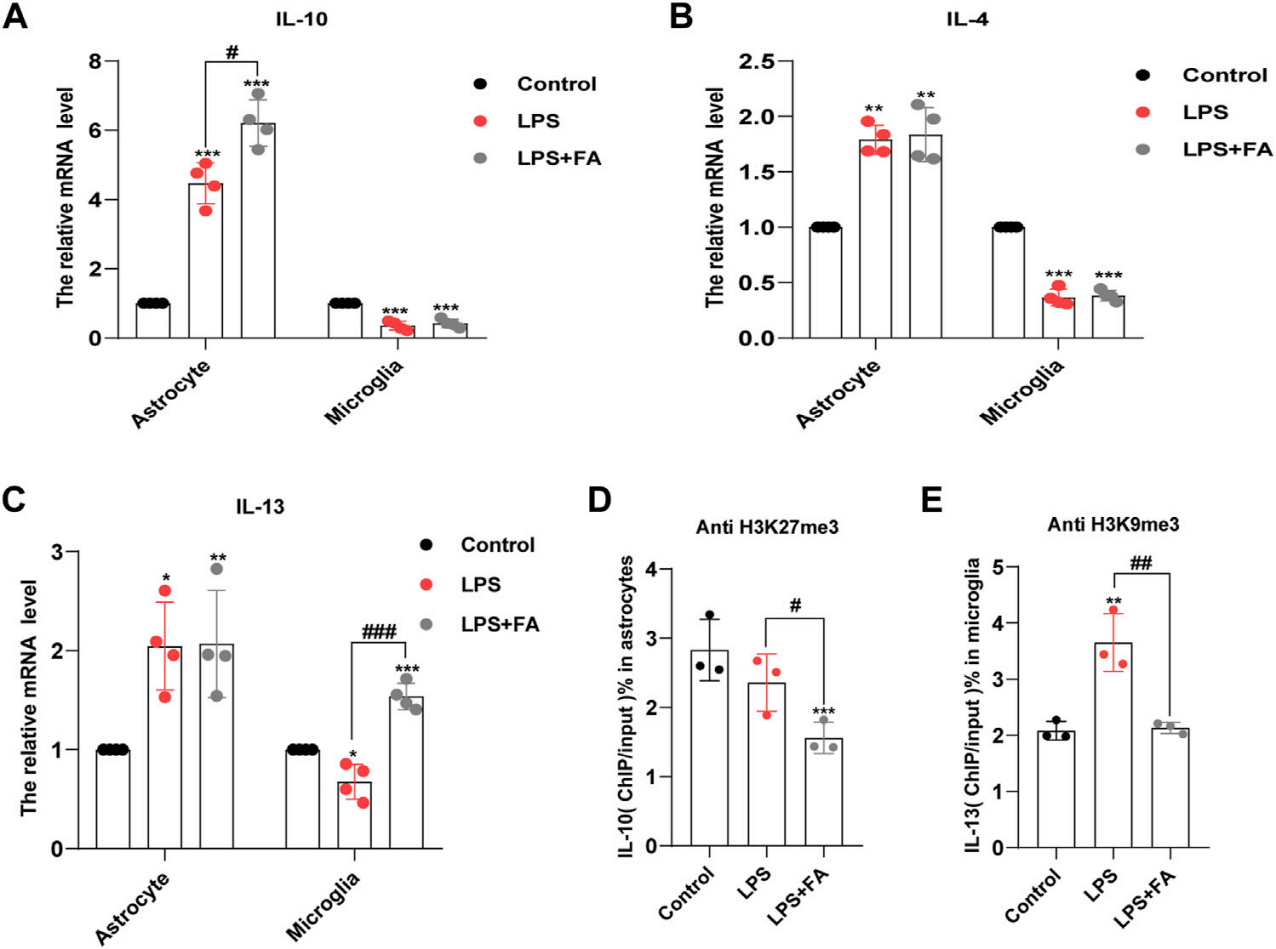
FA increased the binding of H3K27me3 with IL-10 promoter, and H3K9me3 with IL-13 promoter in astrocytes and microglia, respectively. **(A)** Quantification of the mRNA levels of IL-10 in primary astrocytes and microglia. FA had an effect on the expression levels of IL-10 only in astrocytes. **(B)** Quantification of the mRNA levels of IL-4 in primary astrocytes and microglia. **(C)** Quantification of the mRNA levels of IL-13. FA had an effect on the expression levels of IL-13 only in microglia. **(D)** Primary astrocytes were immunoprecipitated with anti-H3K27me3 antibody, and the isolated DNA was analyzed by using IL-10 ChIP-qPCR primers. Compared with the LPS group, the level of H3K27me3 in the IL-10 promoter region decreased significantly in FA-treated astrocytes. **(E)** Microglia cells were immunoprecipitated with anti-H3K9me3 antibody, and the isolated DNA was analyzed by using IL-13 ChIP-qPCR primers. Compared with the LPS group, the level of H3K9me3 in the IL-13 promoter regions decreased significantly in FA-treated microglia. Rabbit IgG was used as a negative control. DNA from each ChIP-qPCR sample was normalized by the corresponding input sample. Data: mean ± SEM; *p < 0.05, **p < 0.01, ***p < 0.001 versus the control group; ^#^ *p* < 0.05, ^##^ *p* < 0.01, ^###^ *p* < 0.001 versus the PIA group; *n* = 3–4 per group (one-way ANOVA).

H3K27me3 and H3K9me3 play a role in reducing gene expression. Thus, we predicted that FA might play an anti-inflammatory role by reducing H3K27me3 and H3k9me3, as well as by enhancing the transcriptional expression of anti-inflammatory factors, such as IL-10, IL-4, and IL-13. Therefore, we determined the expression levels of these anti-inflammatory factors by qPCR. The results showed that in astrocytes, the transcription levels of IL-10 (*p* = 0.0082) were significantly increased in the FA treatment group compared with that of the LPS group (Figure 7A), while there was no significant difference in the transcription levels of IL-4 (*p* = 0.7500) and IL-13 (*p* = 0.9494; Figures 7B,C). However, in microglia, the transcription levels of IL-13 increased significantly in the FA treatment group compared with that of the LPS group (*p* = 0.0002), while there was no significant difference in the transcription levels of IL-10 (*p* = 0.4840) and IL-4 (*p* = 0.7104) (Figures 7A–C). Next, we investigated the binding of H3K27me3 to the promoter of IL-10 in astrocytes and that of H3K9me3 to the promoter of IL-13 in microglia. ChIP-qPCR analyses showed that the levels of H3K27me3 were inhibited at the promoter of IL-10 in the FA treatment group (*p* = 0.0432) compared with that of the LPS group, indicating an upregulation of IL-10 in astrocytes (Figure 7D). In microglia, ChIP-qPCR results showed that the levels of H3K9me3 at the IL-13 promoter significantly increased after LPS stimulation (*p* = 0.0072). However, FA also attenuated this binding (*p* = 0.0073; Figure 7E). The above results showed that FA plays an anti-inflammatory role by regulating the expression of different anti-inflammatory factors in astrocytes and microglia through H3K27me3 and H3K9me3 modifications, respectively.

**FIGURE 8:**
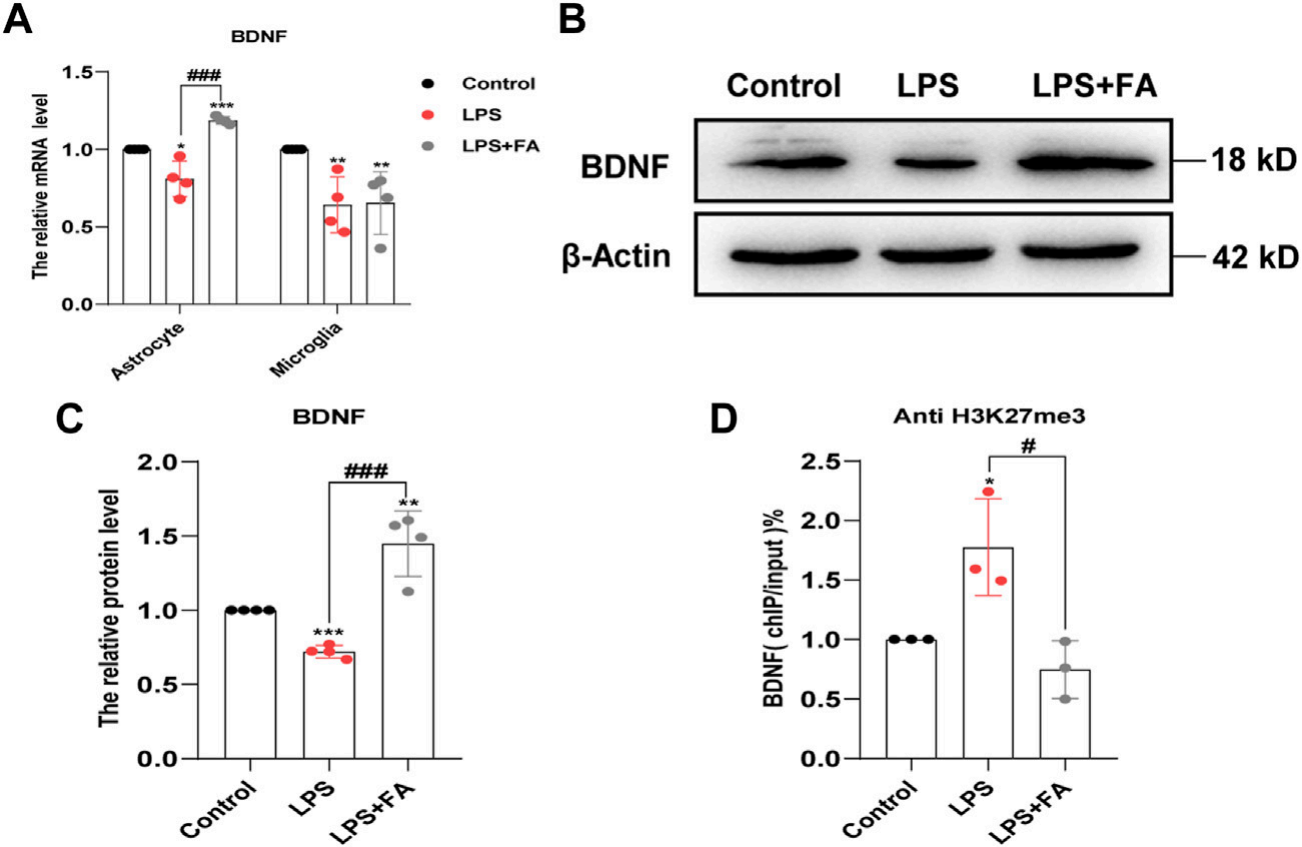
FA regulated the expression of BDNF in astrocytes. **(A)** Quantification of the mRNA levels of BDNF in primary astrocytes and microglia. FA had an effect on the BDNF levels only in astrocytes. **(B,C)** Representative images and quantification of Western blotting analysis of the BDNF expression in astrocytes. **(D)** Primary astrocytes were immunoprecipitated with anti-H3K27me3 antibody, and the isolated DNA was analyzed by using BDNF ChIP-qPCR primers. Rabbit IgG was used as a negative control. DNA from each ChIP-qPCR sample was normalized by the corresponding input sample. Compared with the LPS group, the level of H3K27me3 in the BDNF promoter region decreased significantly in FA-treated astrocytes. Data: mean ± SEM; *p < 0.05, **p < 0.01, ***p < 0.001 versus the control group; ^#^ *p* < 0.05, ^###^ *p* < 0.001 versus the PIA group; *n* = 3–4 per group (one-way ANOVA).

Brain-derived neurotrophic factor (BDNF) plays an important role in the pathogenesis of depression (Hill et al., 2015). We further examined whether FA plays an antidepressant role by regulating BDNF expression. qPCR and Western blotting results showed that FA could reverse the decrease in BDNF expression after LPS treatment in astrocytes (*p* = 0.0007), but not in microglia (Figures 8A–C). The ChIP-qPCR results further showed that the levels of H3K27me3 at the BDNF promoter were significantly increased in LPS-treated astrocytes, while FA attenuated the enrichment change (*p* = 0.0198; Figure 8D). Therefore, FA could also regulate BDNF expression through H3K27me3 modification in astrocytes, and it plays an antidepressant role in the PIA model.

### 3.5 Folic Acid Inhibited the Expression of EZH2 in Astrocytes and Increased the Expression of KDM4A in Microglia

**FIGURE 9:**
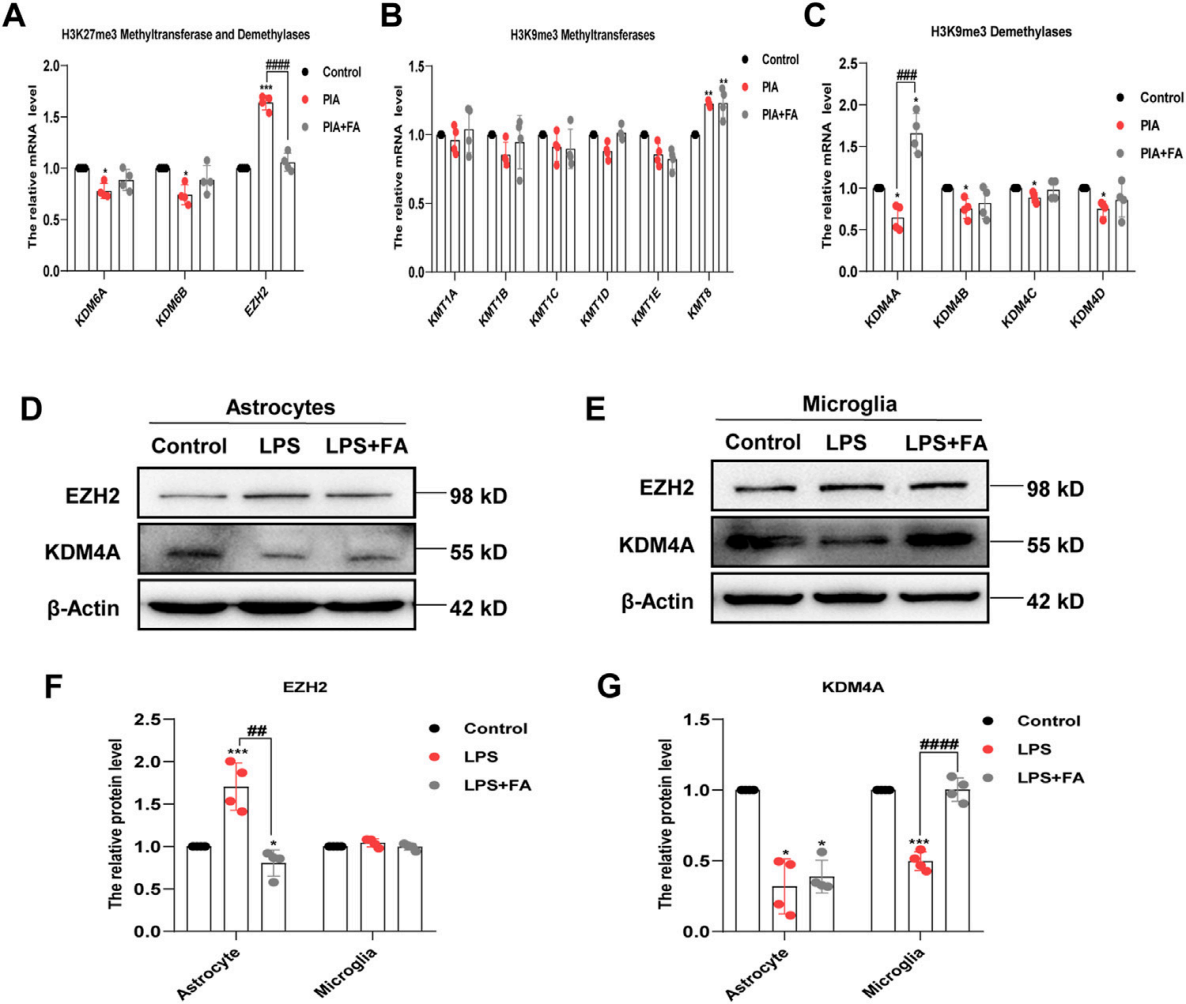
FA regulated different histone methylation modified enzymes in astrocytes and microglia. **(A)** Quantitative RT-PCR showed the expression levels of histone H3K27me3 methyltransferase and demethylases. Compared with the PIA group, the mRNA level of EZH2 in the FA-treated group decreased significantly. **(B)** Quantitative RT-PCR showed the expression levels of histone H3K9me3 methyltransferases. Compared with the PIA group, there was no significant change in the mRNA level of histone H3K9me3 methyltransferases after FA treatment. **(C)** Quantitative RT-PCR analysis showed the expression levels of histone H3K9me3 demethylases. Compared with the PIA group, the mRNA level of KDM4A in the FA-treated group increased significantly. **(D–G)** Representative images and quantification of Western blotting analysis of the expression levels of EZH2 and KDM4A in primary astrocytes and microglia. Data: mean ± SEM; *p < 0.05, **p < 0.01, ***p < 0.001 versus the control group; ^##^ *p* < 0.01, ^###^ *p* < 0.001, ^####^ *p* < 0.0001 versus the PIA group; *n* = 4 per group (one-way ANOVA).

Histone methylation is controlled by histone methyltransferases and histone demethylases. We further detected the changes in related methylases/demethylases that regulate histone H3K27me3/H3K9me3 modification. The results showed that the mRNA levels of H3K27me3 demethylases KDM6A and KDM6B were decreased in the PIA model, but there was no significant change in the FA treatment group. However, FA treatment reversed the increased expression of H3K27me3 methyltransferase EZH2 induced by PIA *in vivo* (*p* < 0.0001; Figure 9A). The mRNA level of H3K9me3 demethylase KDM4A was significantly decreased by PIA treatment, which could be reversed by FA treatment (*p* = 0.0004; Figures 9B,C).

We further identified changes in the related enzymes in primary cultured astrocytes and microglia. Western blotting showed that the protein level of EZH2 was only significantly decreased in astrocytes in the FA treatment group compared with that in the LPS group (*p* = 0.0013), but the change in KDM4A was not significant (*p* = 0.5652). In microglia, compared with the LPS group, the FA treatment group demonstrated reversal of the increased expression of KDM4A caused by LPS treatment (*p* < 0.0001), but had no significant effect on the level of EZH2 (*p* = 0.1736; Figures 9D–G). Overall, we conclude that FA may regulate IL-10 and BDNF expression in astrocytes through EZH2-mediated regulation of H3K27me3, as well as regulate IL-13 expression in microglia through KDM4A-mediated regulation of H3K9me3.

**FIGURE 10:**
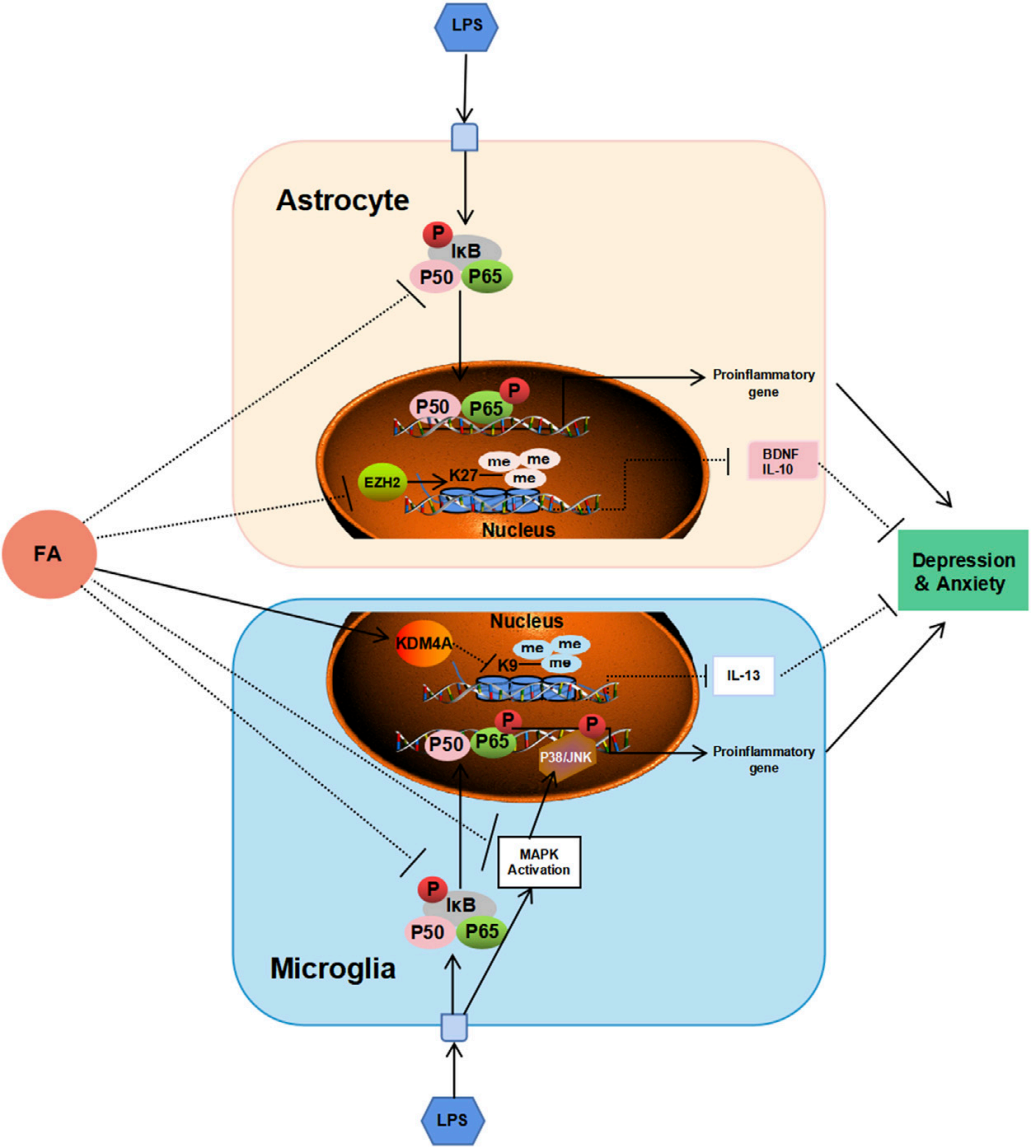
Schematic diagram shows the possible mechanism of FA against depression and anxiety in astrocytes and microglia. FA could improve neonatal encephalitis and PIA-induced neuropsychiatric disorders through different regulatory mechanisms in different glial cells. FA prevents inflammatory events by interfering with NF-κB signaling pathway in astrocytes, and both the NF-κB signaling pathway and P38/MAPK signaling pathway in microglia. In addition, FA plays important roles in PIA models through EZH2/H3K27me3-mediated IL-10 and BDNF in astrocytes, and KDM4A/H3K9me3-mediated IL-13 in microglia, respectively.

Taken together, we discovered the role of FA in astrocyte and microglial activation in PIA-induced depression and anxiety, indicating that it could serve as a potential therapeutic candidate for the prevention and treatment of depression and anxiety (Figure 10). At the same time, we found that the therapeutic and biological effects of FA on different glial cells were different.

## 4 Discussion

FA plays various important roles in newborns, including preventing neural tube malformation. It also affects a variety of physiological functions (Blencowe et al., 2010; Murray et al., 2018). In addition, FA, as a methyl donor for carbon metabolism, plays an important role in cell proliferation, cell differentiation, and gene transcription regulation by affecting the methylation of histones, DNA and RNA (Leung et al., 2013). Mounting evidence has shown that FA is of great pathophysiological relevance in the neuroinflammatory response (Zhang et al., 2008; Feng et al., 2011; Cianciulli et al., 2016; Samblas et al., 2018). However, the roles and mechanisms of FA in neonatal encephalitis and adult behavior disorders induced by PIA remain unclear. Our study showed, for the first time, that FA has anti-depression and anti-anxiety potential by inhibiting inflammatory reactions in mouse brain microglia and astrocytes, which is caused by postnatal period inflammatory events. Importantly, we found that the mechanisms by which FA inhibits inflammatory signaling events are different in astrocytes and microglia, and that the regulation of the expression of downstream target genes is through different epigenetic mechanisms.

Current animal models based on induction with LPS administration are useful for screening candidate anti-depression and anti-anxiety drugs, as well as for investigating disease pathogenesis (Li et al., 2017; Arioz et al., 2019; Ali et al., 2020a; Li et al., 2021a). As a lipophilic molecule, LPS can pass through a healthy or damaged blood–brain barrier (BBB) into the brain. Systemic injection of LPS can induce depressive-like behavior characterized by behavioral changes similar to symptoms of acute systemic inflammation or infection, leading to exacerbations of depression (Maes et al., 2012). In our study, neonatal mice received LPS on postnatal day 3 to generate the PIA model. Validated and standardized behavioral tests quantitatively confirmed that FA could improve neonatal inflammation–induced behavioral abnormalities in adulthood, which is consistent with the results of previous studies that FA could ameliorate depressive-like behavior in the model of chronic unpredictable mild stress or restraint stress (Zhou et al., 2020). However, it is unclear whether FA plays a role in the PIA model. We found for the first time that early treatment of FA could improve neonatal encephalitis and behavioral abnormalities in adulthood, and FA has high safety in neonatal medication. In addition, neuroinflammation induced by glial cells’ activation can cause neuronal cell damage (Hopkins and Rothwell, 1995; Qin et al., 2004). Neuronal damage is an important mechanism underlying neuropsychiatric disorders such as depression and anxiety (Zhou et al., 2014; Tunc-Ozcan et al., 2019; Xu et al., 2019). In the future, this will be a key point of our research.

FA can inhibit not only microglia activation but also astrocyte activation, and regulate different inflammatory signaling pathways. A recent study showed that FA exerts anti-inflammatory effects *in vitro* (Liu et al., 2011). However, whether the effects of FA in the PIA model are related to glial cell activation *in vivo* remains unknown. Our present observations showed that FA has an anti-inflammatory effect on the PIA model and can inhibit the response not only in microglia but also in astrocytes in a model of inflammation-related depression in neonatal mice. Astrocytes, which make up the majority of glial cells, also play an important role in neuroinflammation (Colombo and Farina, 2016). To our knowledge, this is the first study to show that FA can inhibit astrocyte activation in the neonatal brain using the PIA model. Microglia and astrocyte inflammatory responses play an important role in depression (Yirmiya et al., 2015; Kaufmann and Menard, 2018). Therefore, FA improves behavioral abnormalities in the PIA model by inhibiting glial cell activation and inflammatory responses. We next detected multiple signaling pathways affecting inflammation in glial cells, such as NF-κB and MAPK. The MAPK signaling pathway has been reported to be involved in cell proliferation, differentiation, apoptosis, and inflammation (Chu et al., 2019; Papaconstantinou, 2019). The predominant effect of the MAPK signaling pathway is the activation of three distinct cascades: the extracellular signal-related kinases, c-Jun N-terminal kinases, and p38 pathways (Sun et al., 2015). The MAPK signaling pathway plays a vital role in inflammation (Tan et al., 2019). The NF-κB signaling pathway is a classical signaling pathway associated with inflammation, depression, and anxiety (Zhang et al., 2018). Although previous studies have shown that FA could affect the MAPK signaling pathway and NF-κB signaling pathway activation in microglia (Cianciulli et al., 2016), its effect and mechanism on astrocytes are unknown. We found that FA inhibited the NF-κB signaling pathway in two types of glial cells. However, MAPK signaling was effective in microglia but not in astrocytes. Finally, the results indicate that FA has various anti-inflammatory effects in different glial cells.

As a carbon unit donor, FA affects epigenetic modification and is essential in biochemical processes (Li et al., 2015). Previous studies have shown that epigenetic mechanisms are important in depression (Ernst et al., 2009; Penner-Goeke and Binder, 2019). In arsenic-induced heart abnormalities in fetal rats, FA could play a protective role by lowering the acetylation levels of histone H3K9 (Na et al., 2020). However, in our study, FA had no significant effect on the LPS-induced decrease in histone acetylation levels. A previous study showed that FA could inhibit H3K27me3 expression in neural stem cells with neural tube defects (Zhai et al., 2016b). Therefore, we tested the effect of FA on histone methylation in LPS-stimulated primary microglia and astrocytes. Interestingly, our results revealed that the effect of FA on H3K27me3 was observed only in LPS-treated astrocytes, whereas FA mainly reduced H3K9me3 expression in LPS-induced microglia. IL-10 is considered a prototypical anti-inflammatory cytokine that contributes to maintaining and re-establishing immune homeostasis and is produced in glial cells (Chistyakov et al., 2018; Bedke et al., 2019). In astrocytes, FA treatment reduced the “repressive” mark H3K27me3 enrichment at the IL-10 promoter compared to the LPS group, which was consistent with the increased IL-10 expression. IL-13, a vital anti-inflammatory factor, stimulates microglia to adopt an M2 phenotype to resolve inflammation and promote tissue repair (Pandit et al., 2020). In microglia, ChIP-qPCR showed that FA specifically reduced the recruitment of “repressive” mark H3K9me3 to the IL-13 promoter and increased IL-13 production after LPS treatment. In addition, FA can selectively regulate BDNF expression by regulating H3K27me3 in astrocytes rather than microglia, which is important for the progression of depression. Although epigenetic regulation mechanisms mediated by FA have been previously reported, the specific regulatory differences in different cell types have not been analyzed in detail. EZH2, a histone methyltransferase, plays an essential role in the epigenetic maintenance of the H3K27me3 repressive chromatin mark (Yoo and Hennighausen, 2012). In a depression model induced by chronic unpredictable stress, EZH2 inhibitors improved depressive-like behaviors (Wang et al., 2020). In our study, FA decreased H3K27me3 expression in LPS-treated astrocytes through regulation of EZH2 methyltransferase, leading to a decrease in the histone methylation levels of BDNF and IL-10 promoter, which led to increased BDNF and IL-10 expression. However, in microglia, FA has no effect on EZH2 expression but increases KDM4A expression to prevent the recruitment of H3K9me3 in the IL-13 promoter to inhibit inflammatory processes in PIA-induced mood disorders.

Altogether, the present study demonstrated that FA treatment could inhibit microglial and astrocyte activation in the cortex and hippocampus, as well as ameliorate PIA-induced depressive- and anxiety-like behaviors. Using primary astrocytes and microglia cultured *in vitro* and combined with the PIA model, we found different mechanisms of FA in different cell types. Regarding inflammation-related MAPK and NF-κB signaling pathways, the anti-inflammatory mechanisms of FA in astrocytes and microglia were slightly different. Meanwhile, we found that FA can inhibit glial cell activation and pro-inflammatory factor expression, as well as increase anti-inflammatory factors by regulating EZH2-mediated H3K27me3 expression in astrocytes and KDM4A-mediated H3K9me3 expression in microglia. The present data shed new light on the anti-inflammatory mechanisms of FA in microglia and astrocyte activation associated with neonatal acute systemic inflammation–induced depression and anxiety, as well as providing further confirmation that FA may have therapeutic utility in depressive- and anxiety-like behaviors.

## Data Availability

The original contributions presented in the study are included in the article/Supplementary Material; further inquiries can be directed to the corresponding authors.
