# Supplementary material for: Folic Acid Attenuates Glial Activation in Neonatal Mice and Improves Adult Mood Disorders Through Epigenetic Regulation
Source: Front Pharmacol. 2022 Feb 7;13:818423. doi: 10.3389/fphar.2022.818423 (PMC8859176; doi:10.3389/fphar.2022.818423)

Figure2A GFAP **Control**

Cortex

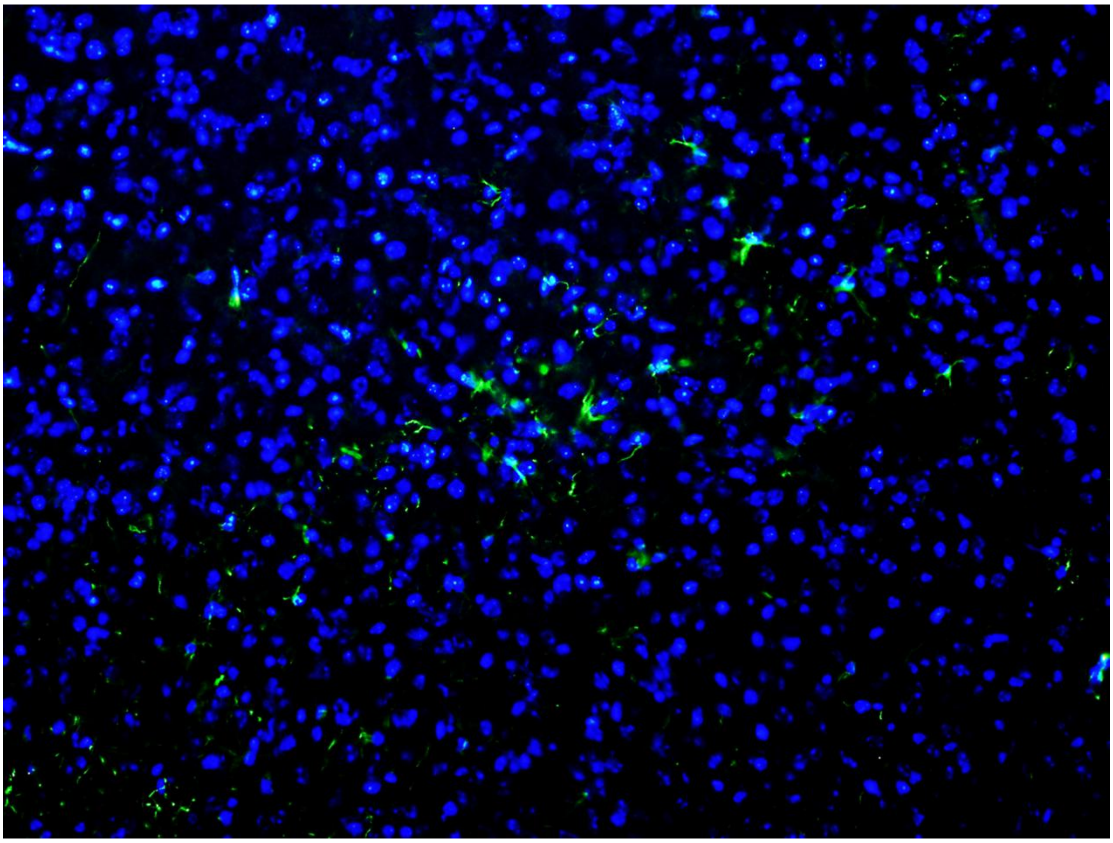

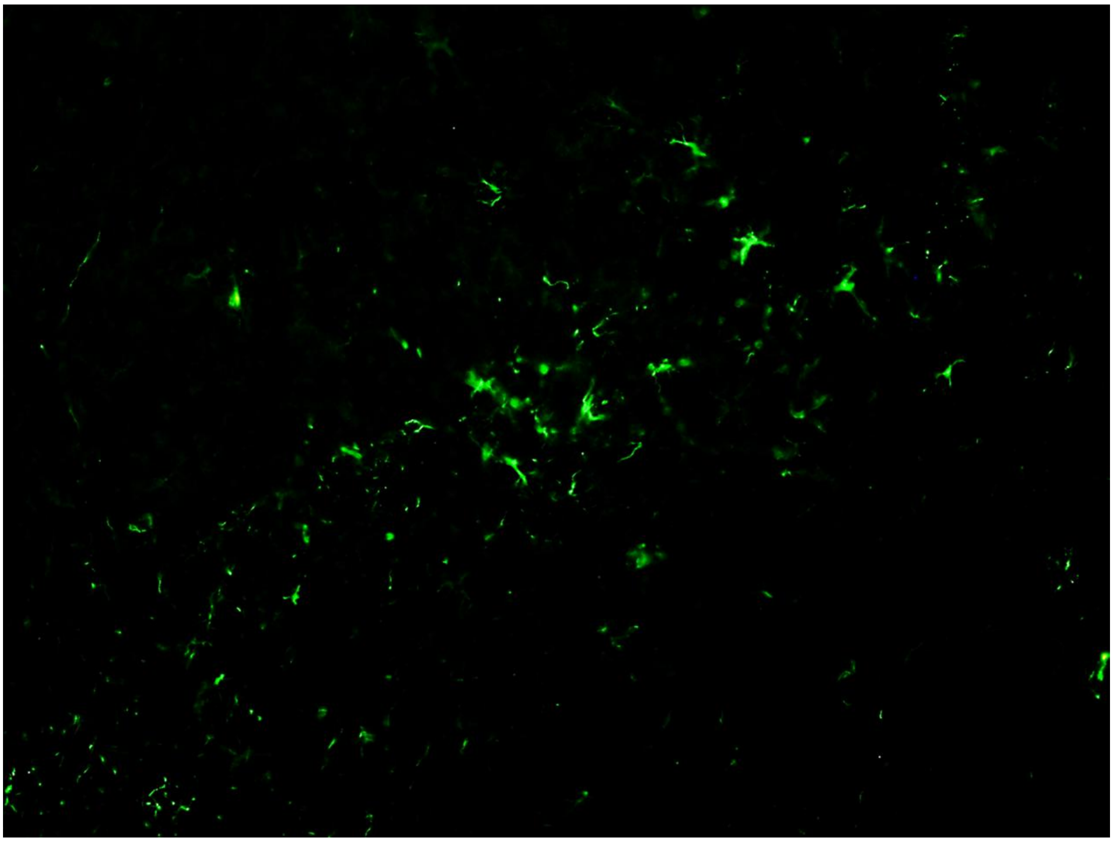

Hippocampus

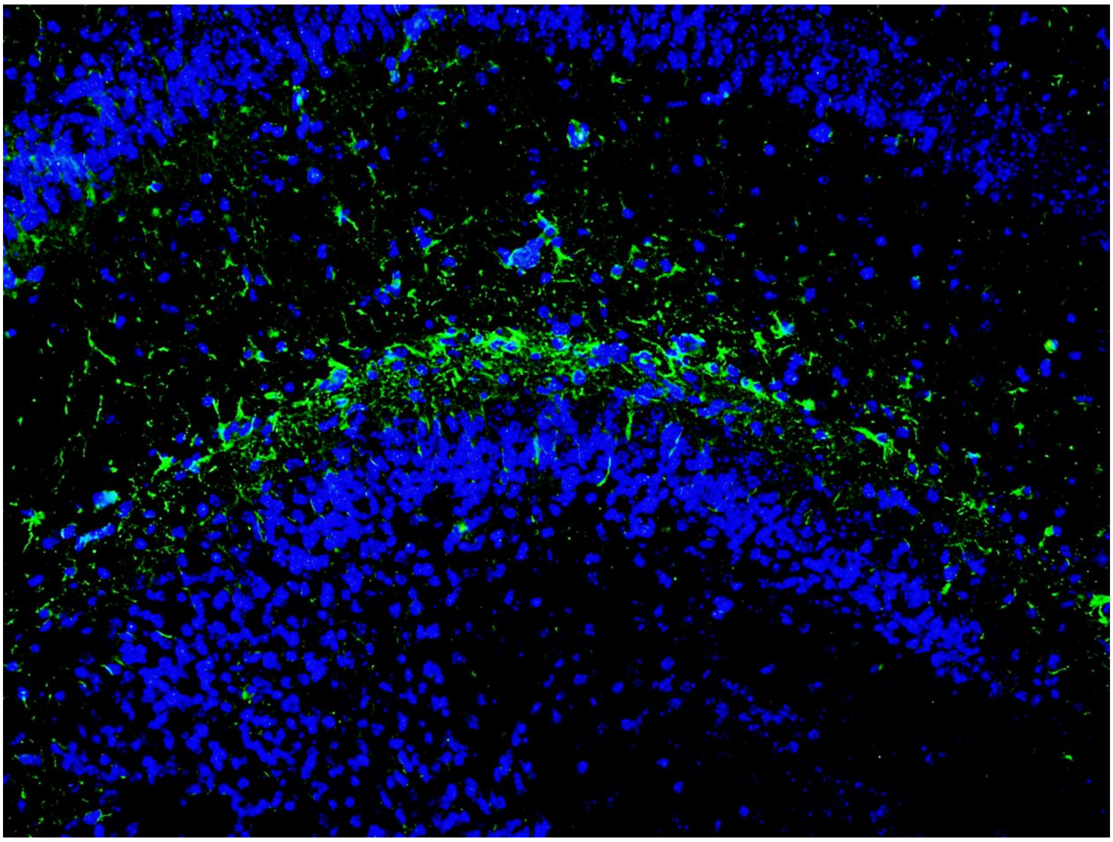

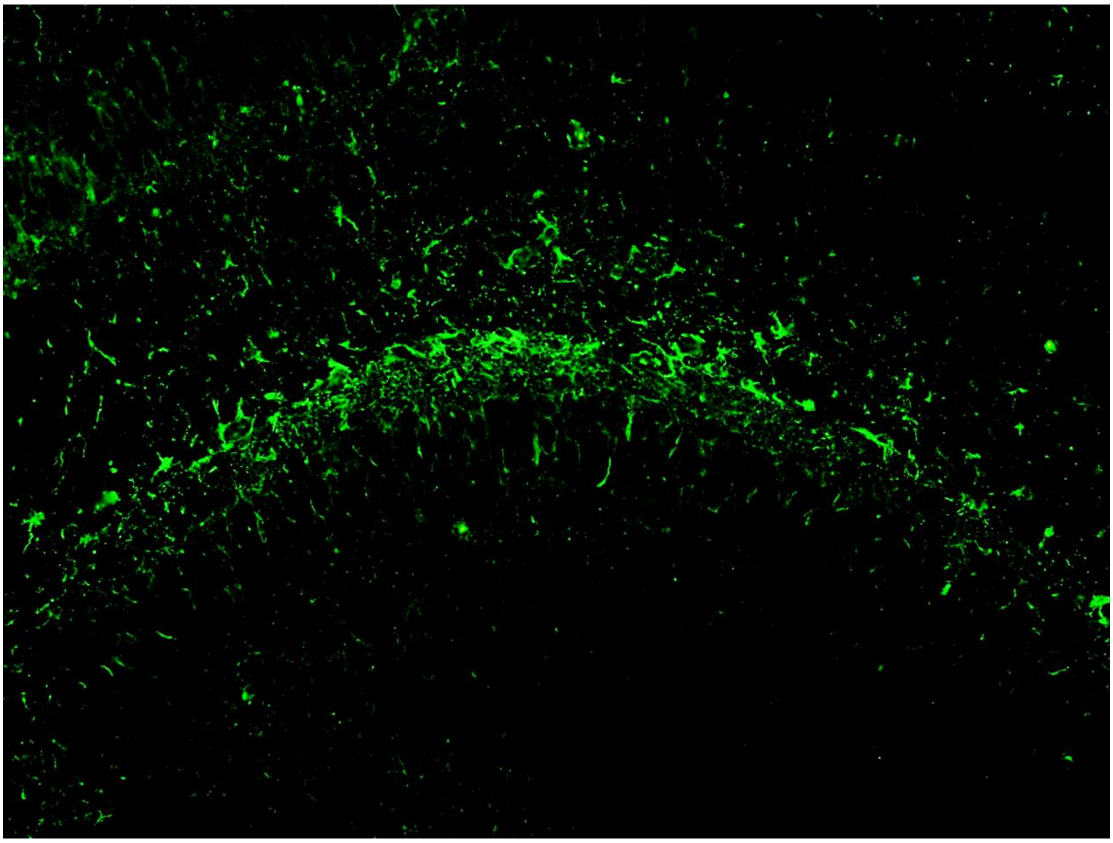

Figure2A GFAP **PIA**

Cortex

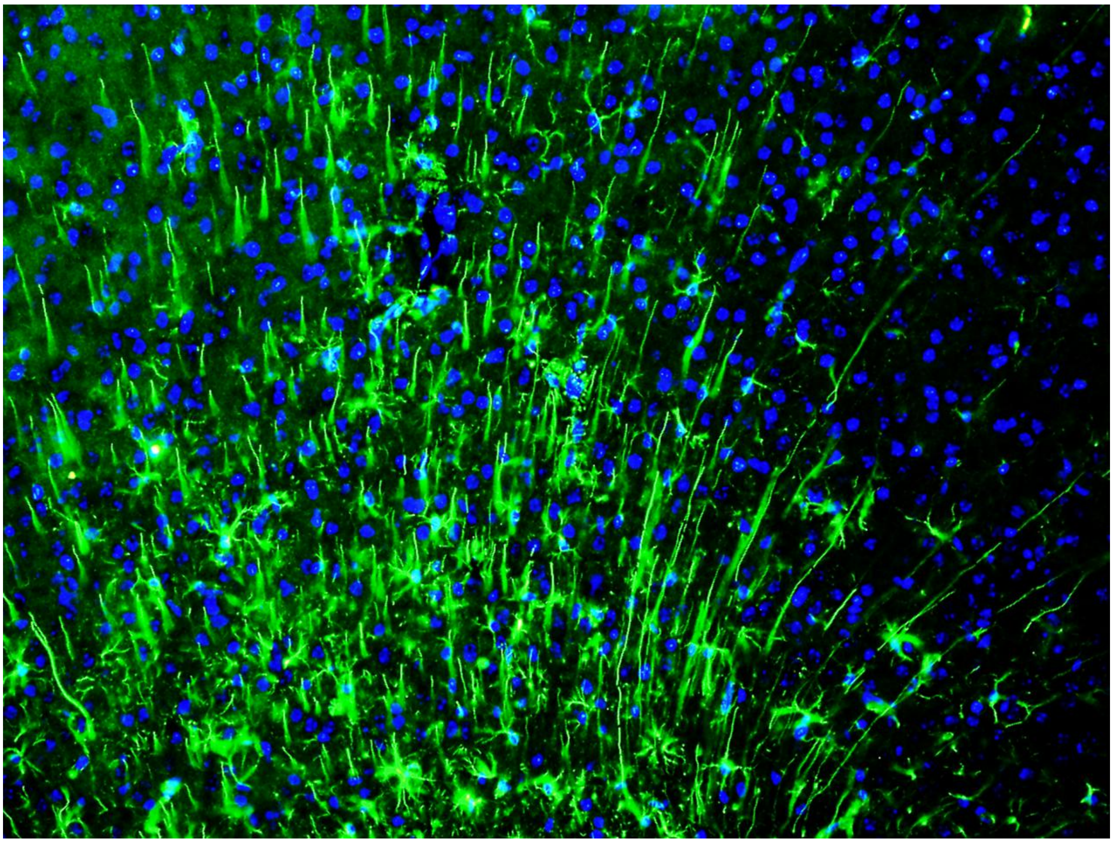

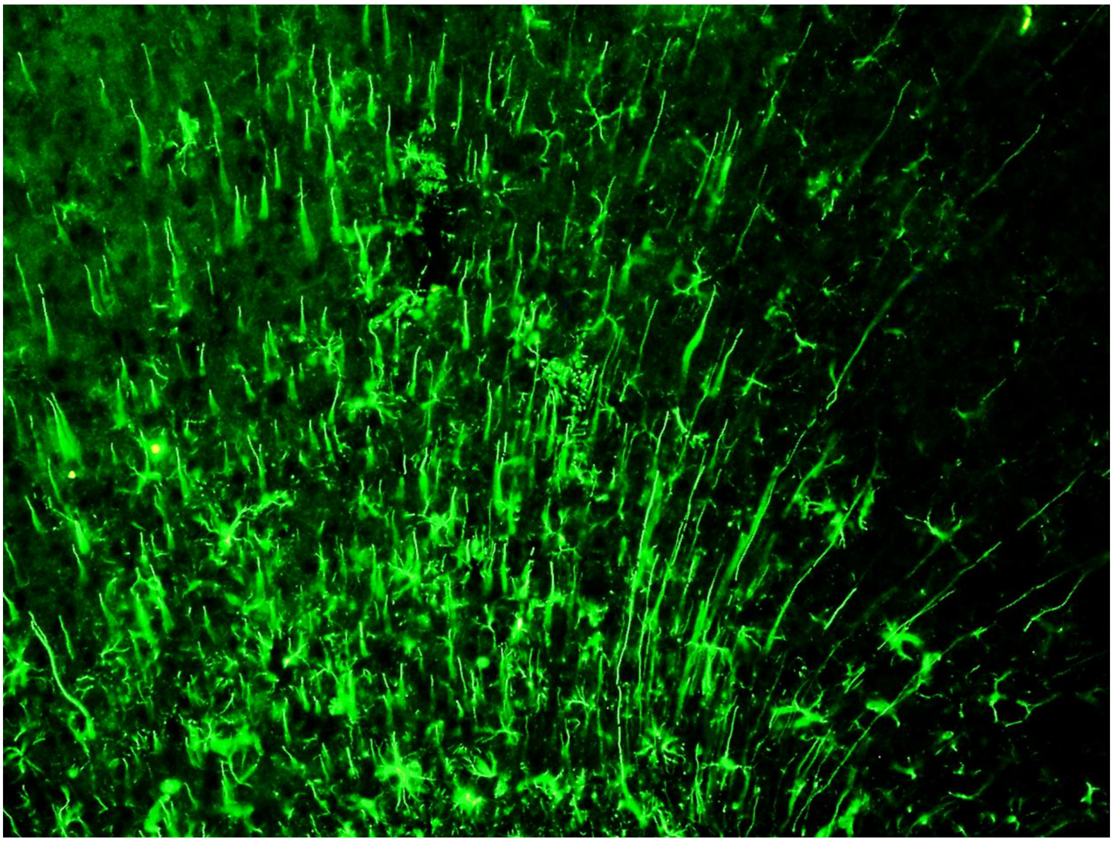

Hippocampus

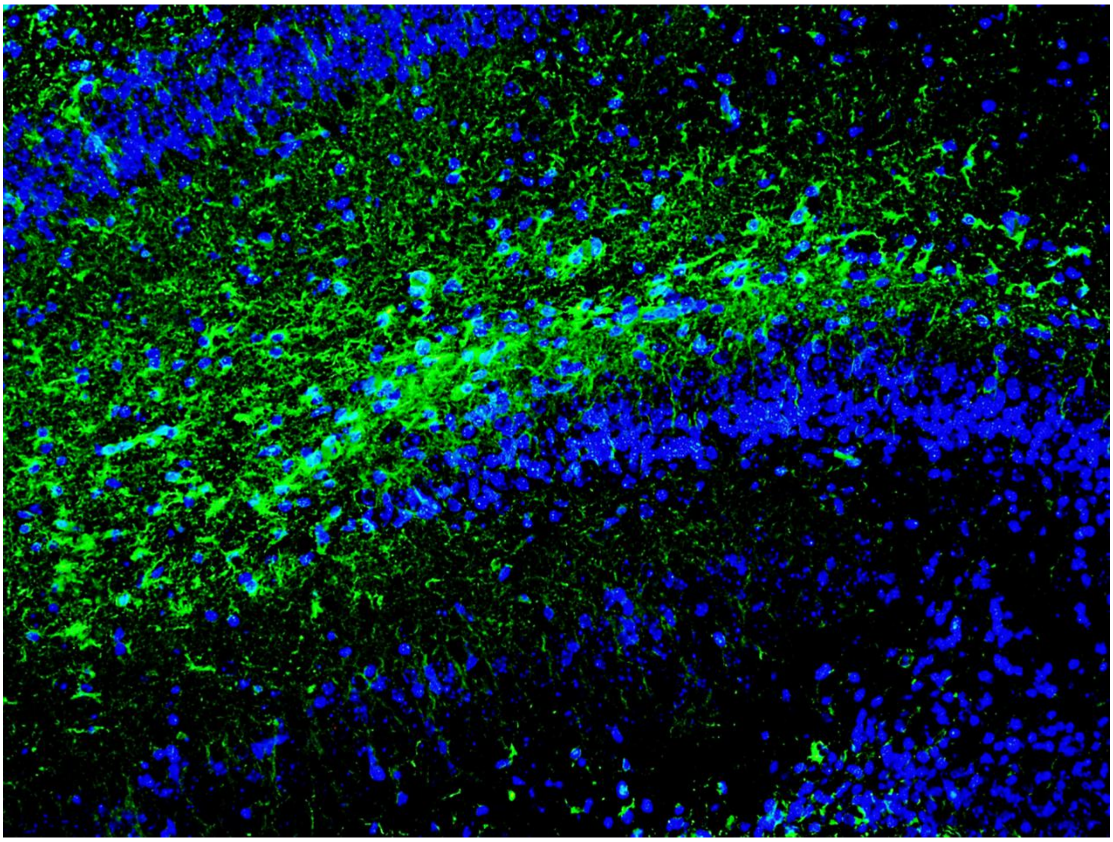

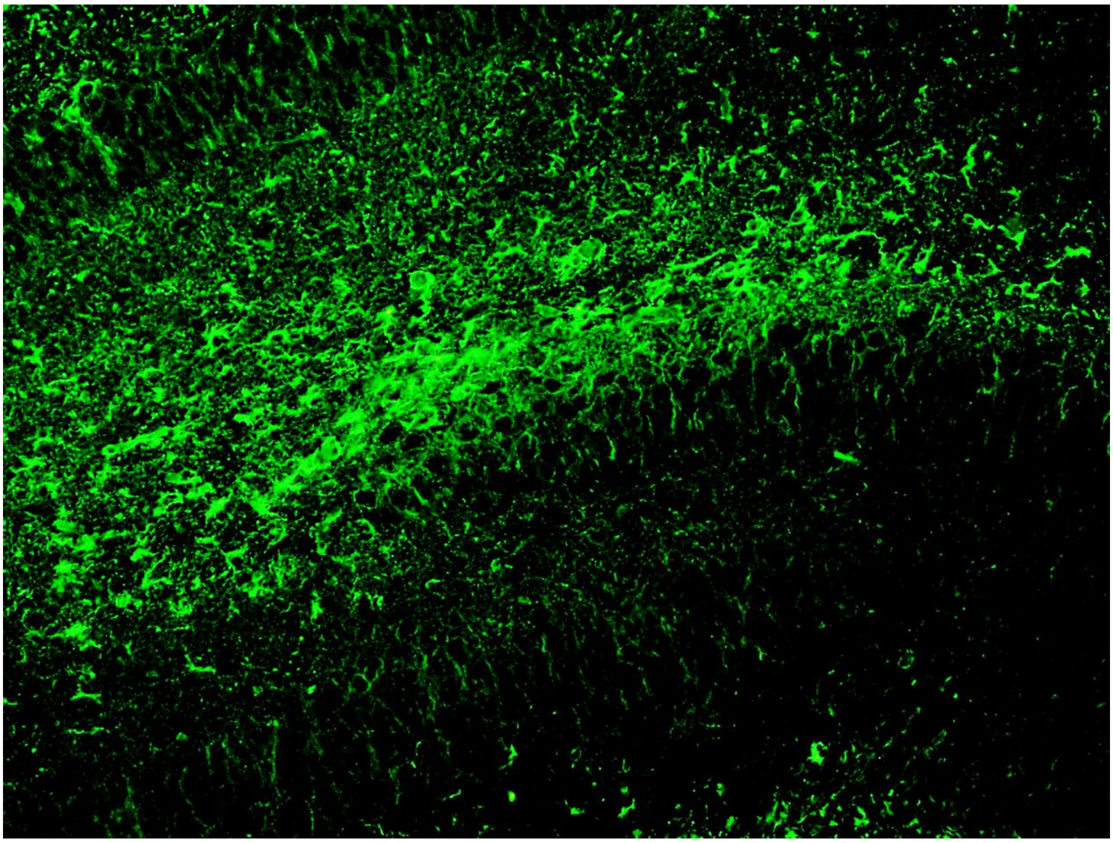

Figure2A GFAP **PIA+FA**

Cortex

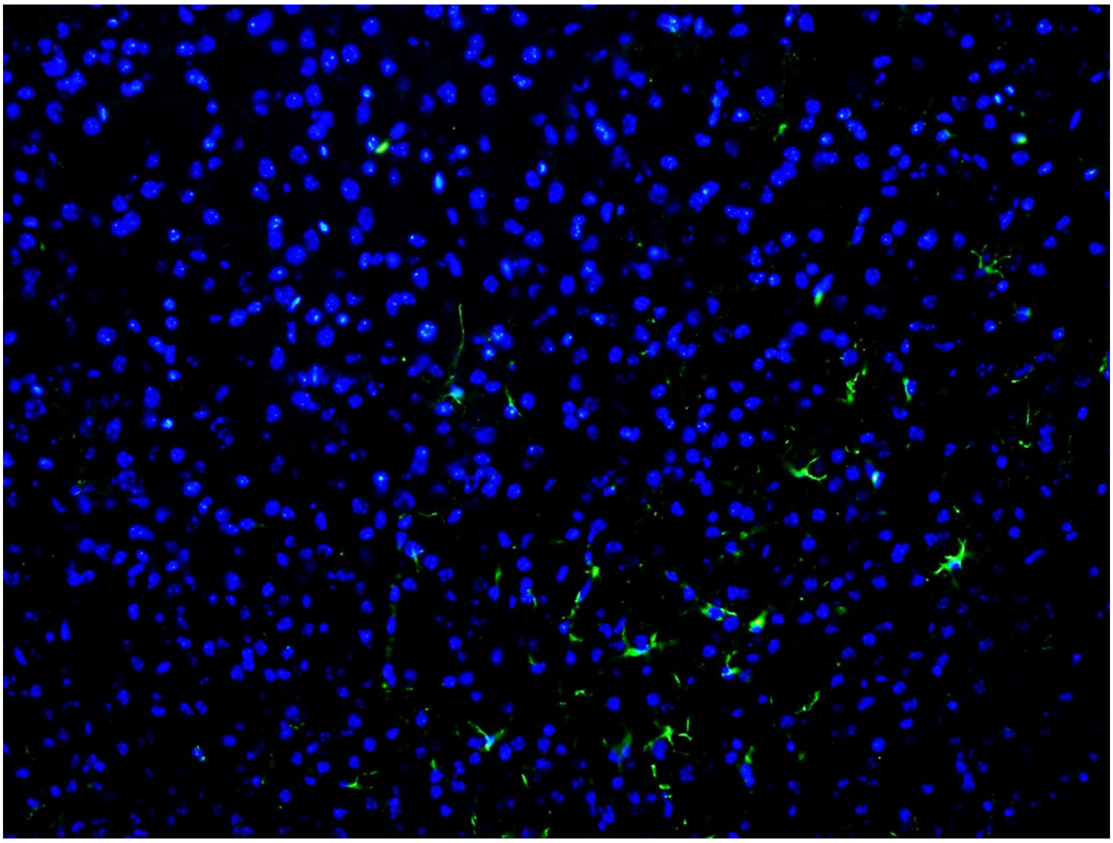

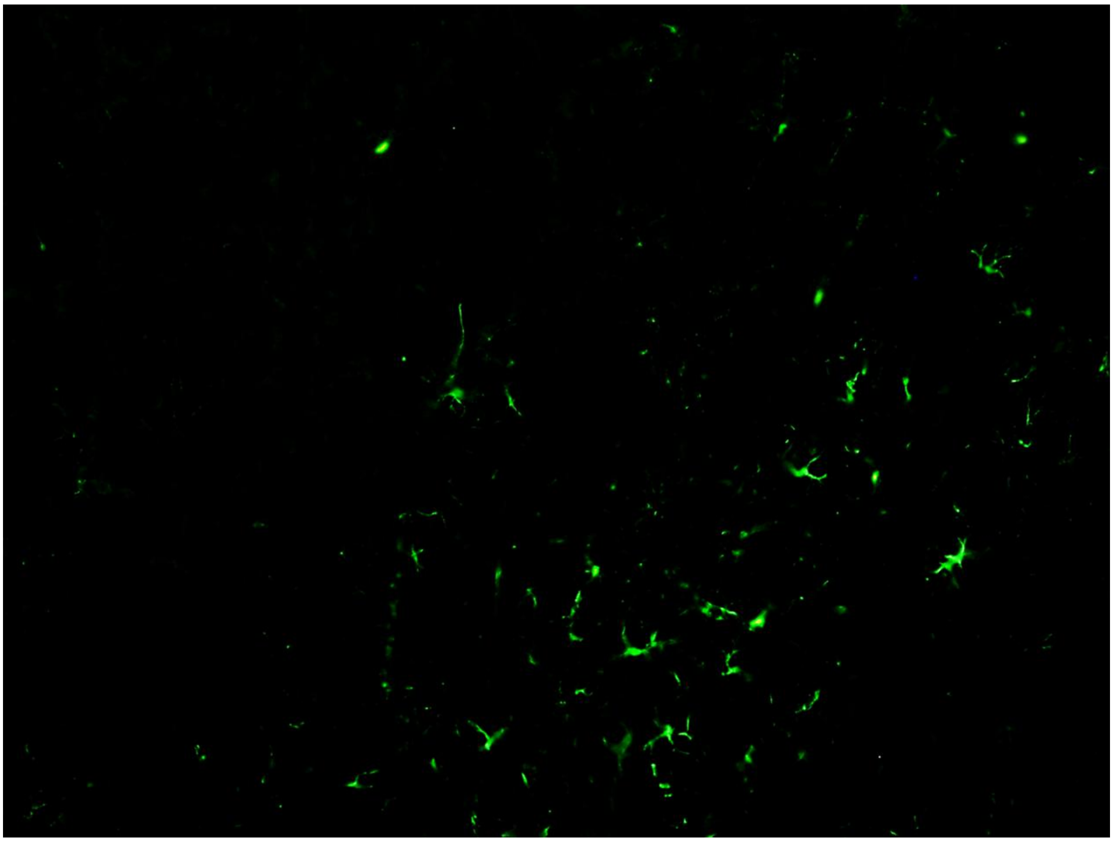

Hippocampus

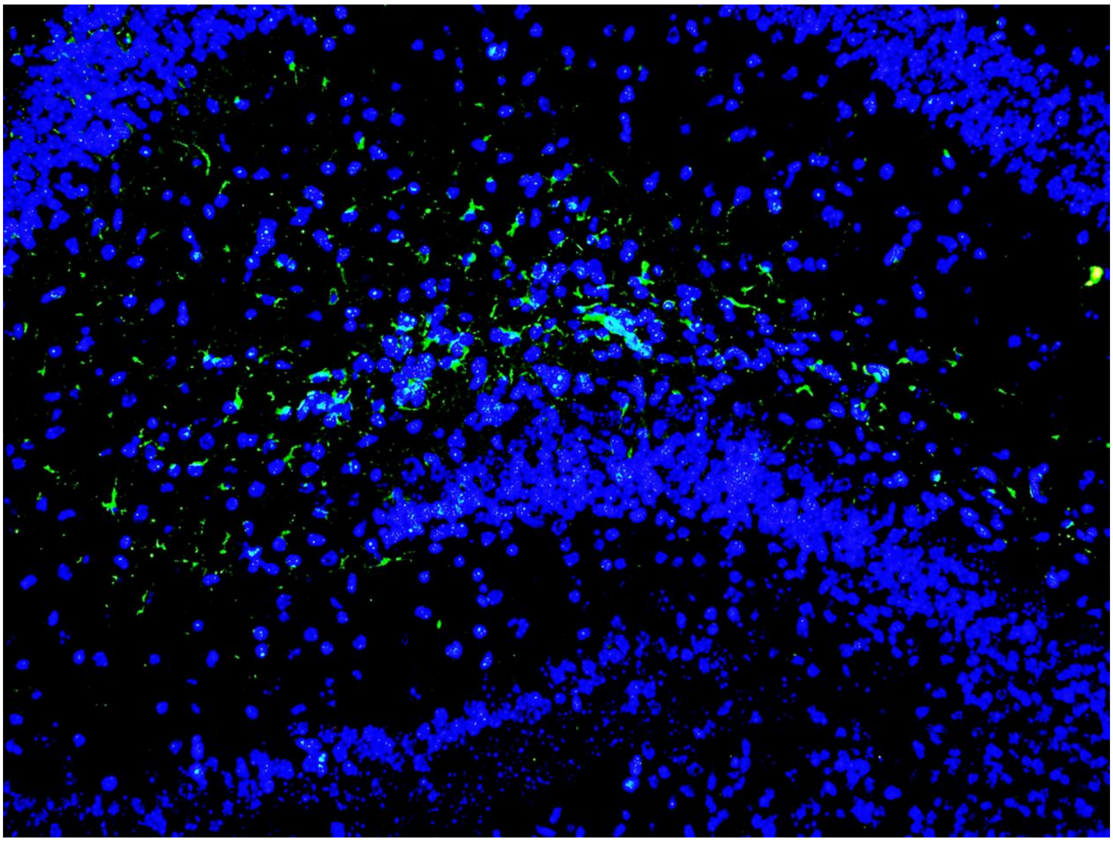

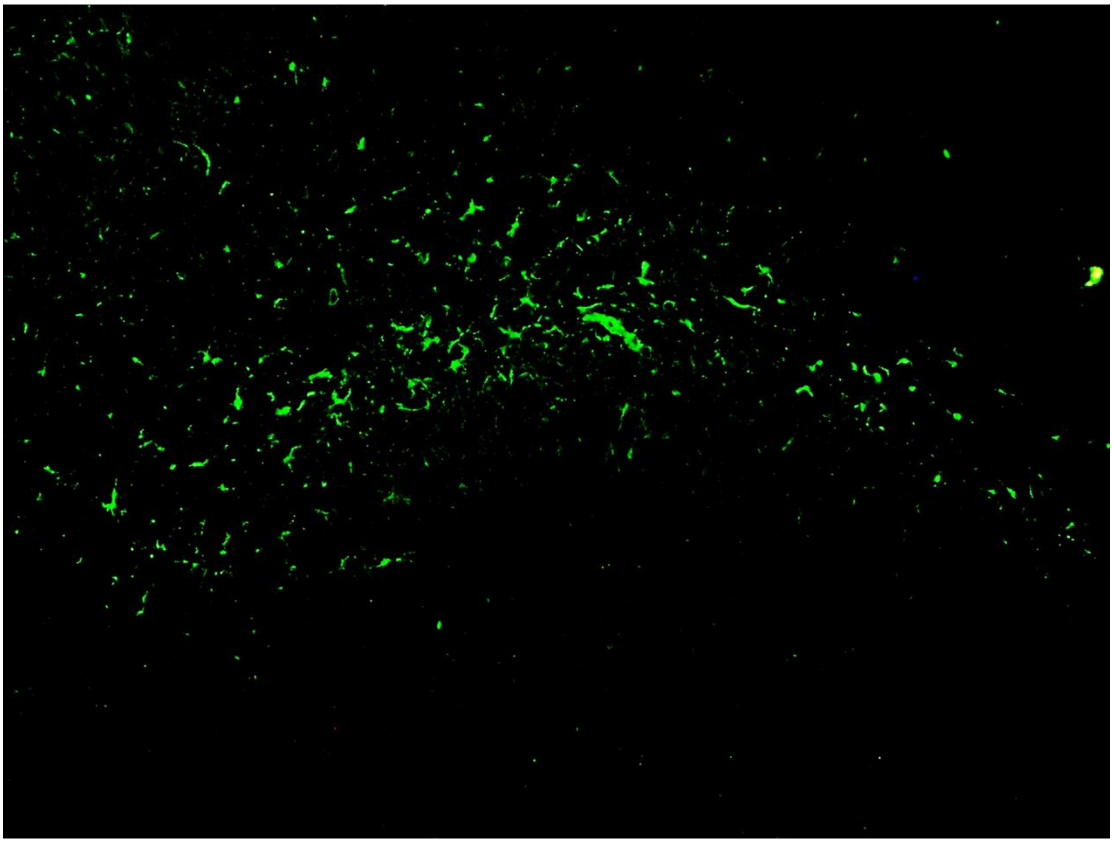

Figure2A Iba1 **Control**

Cortex

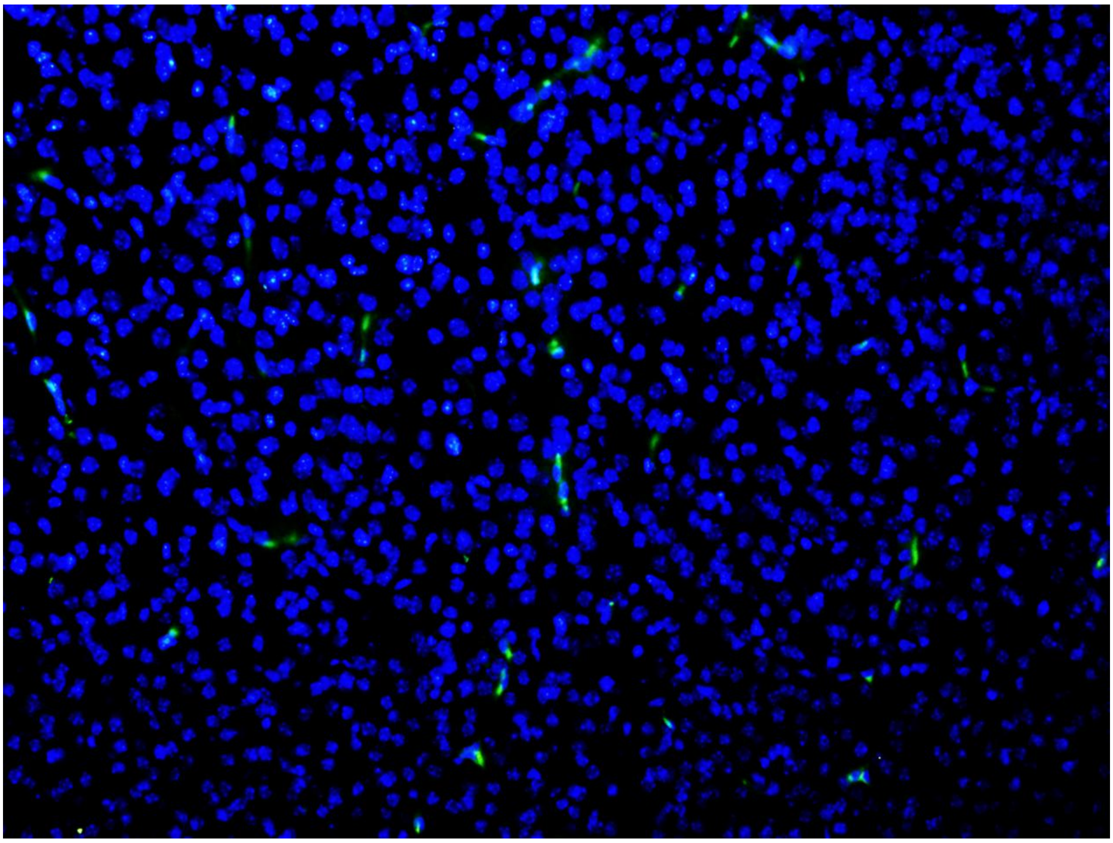

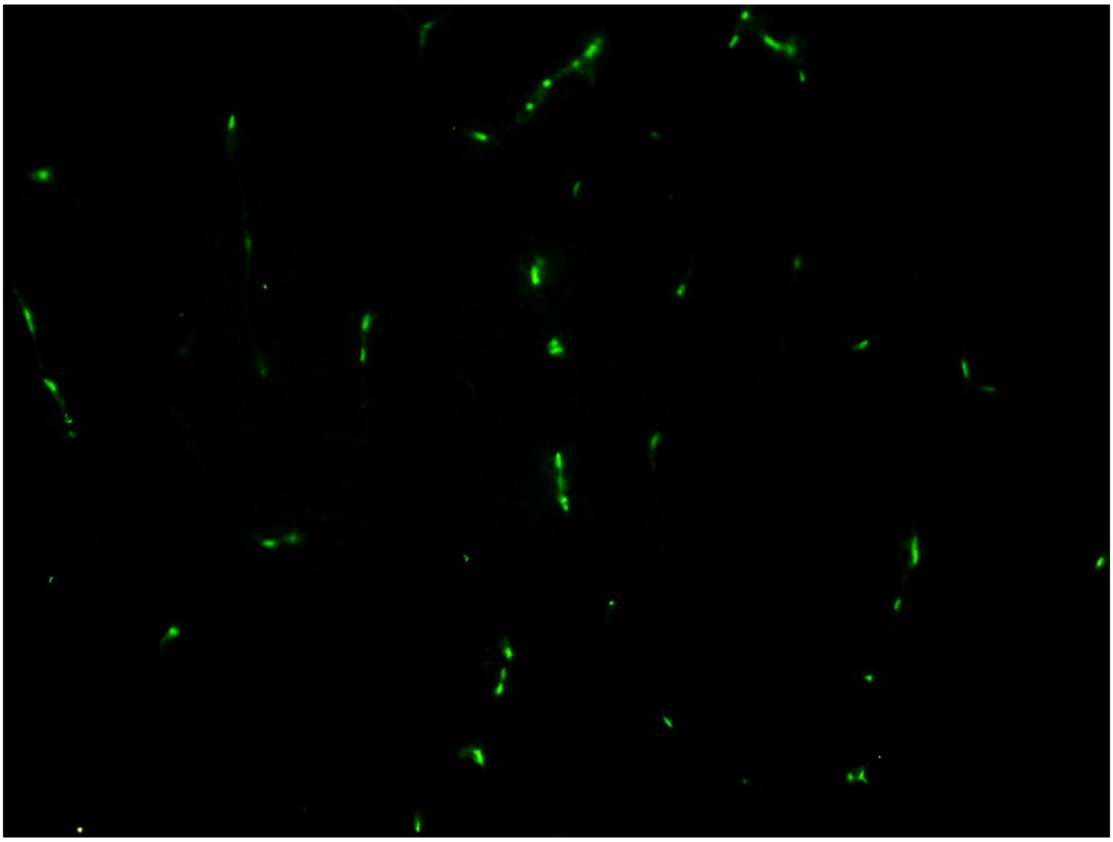

Hippocampus

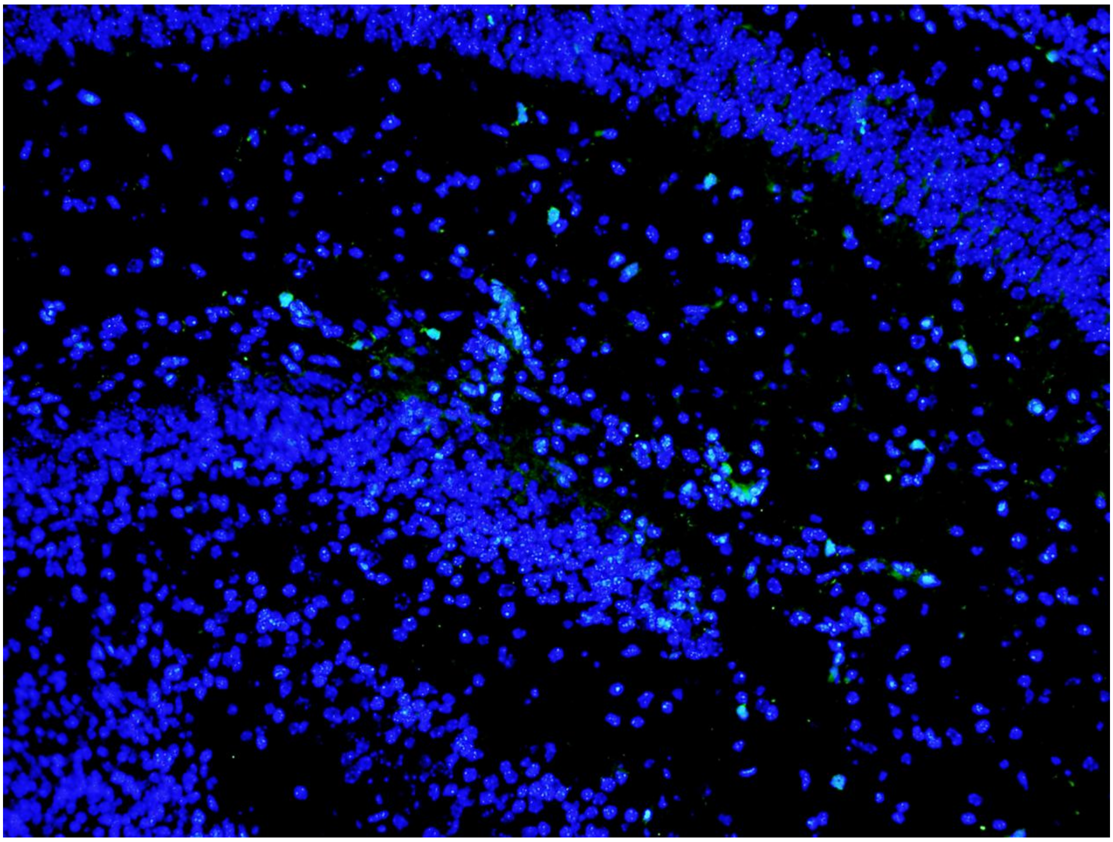

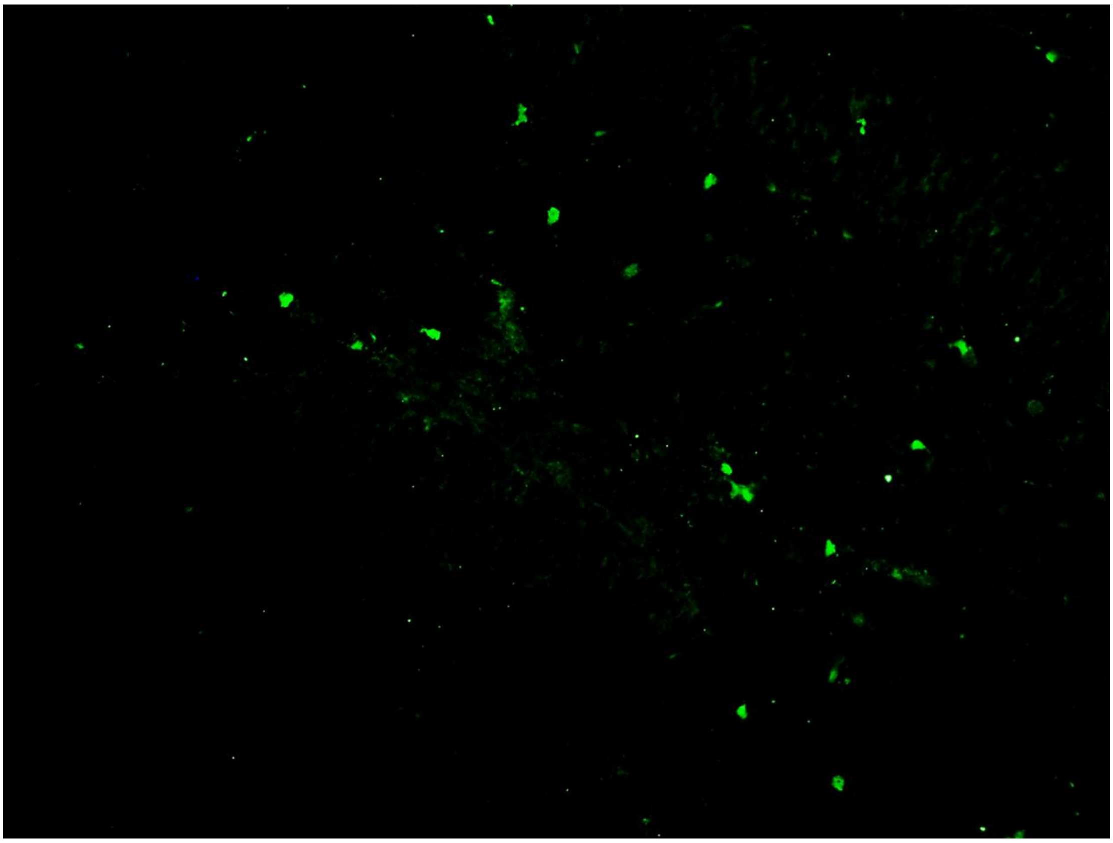

Figure2A Iba1 **PIA**

Cortex

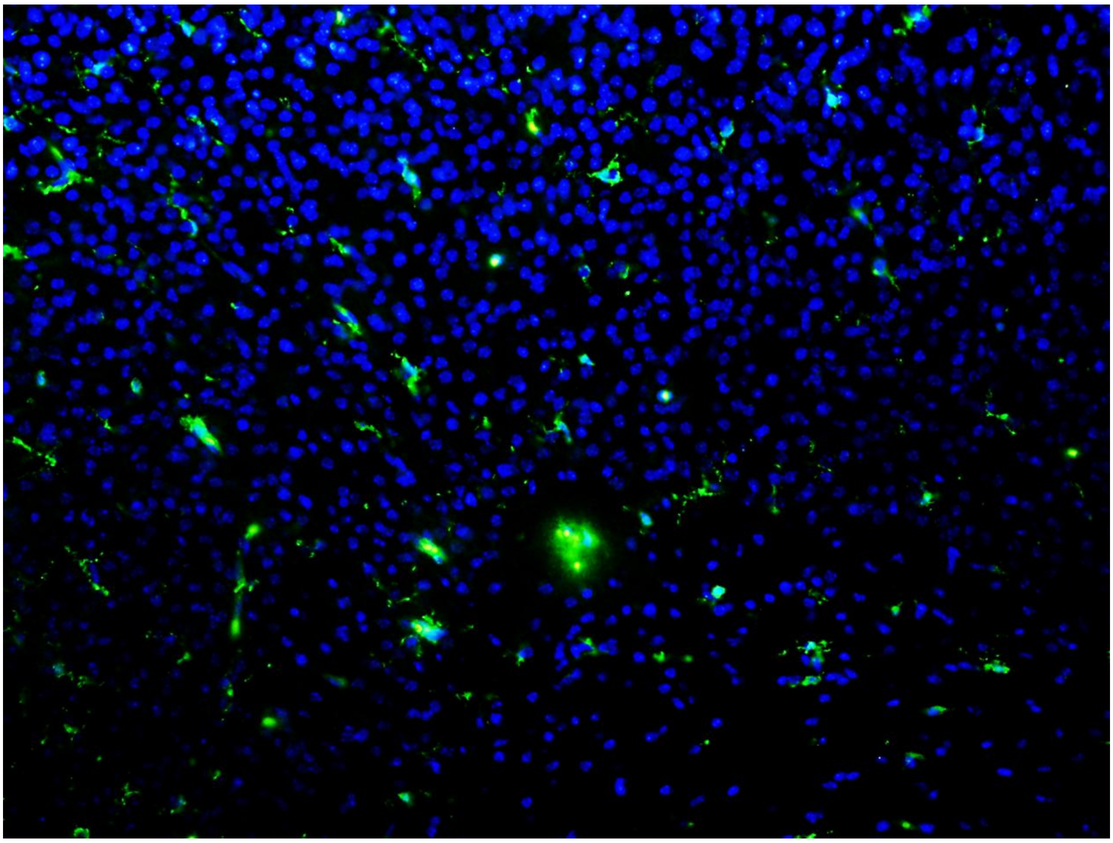

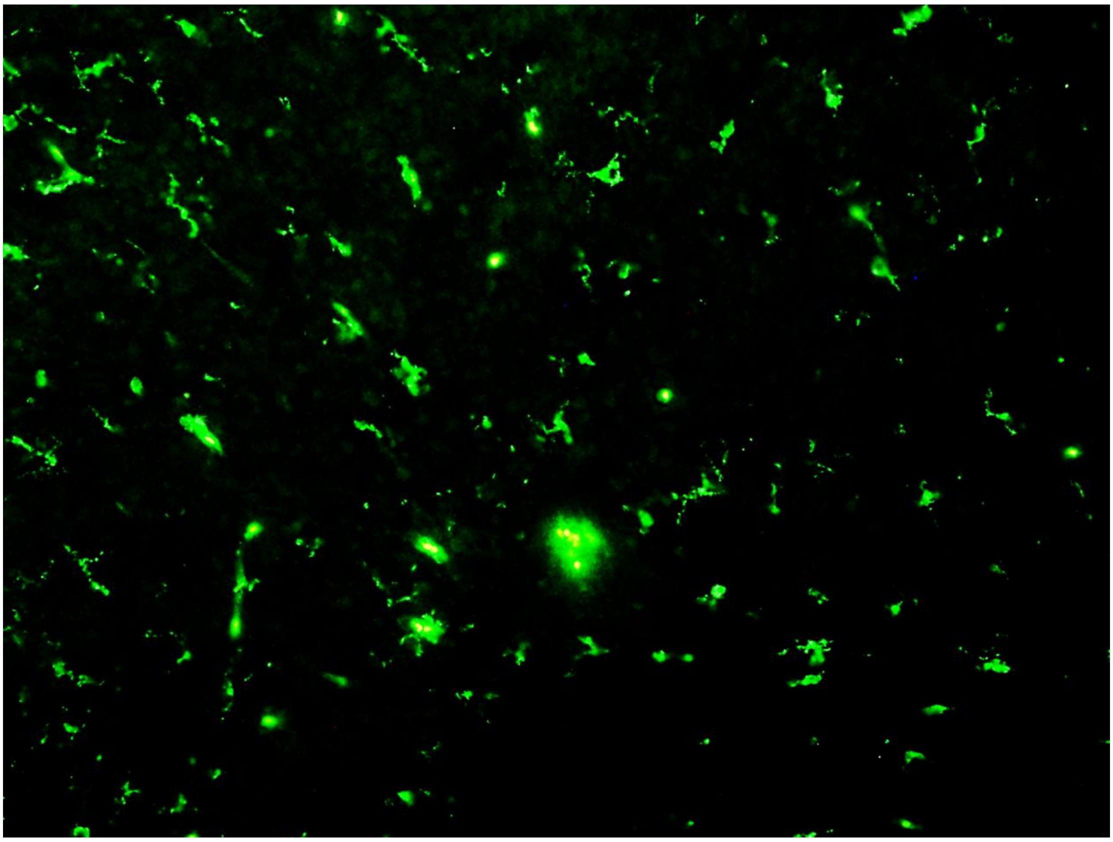

Hippocampus

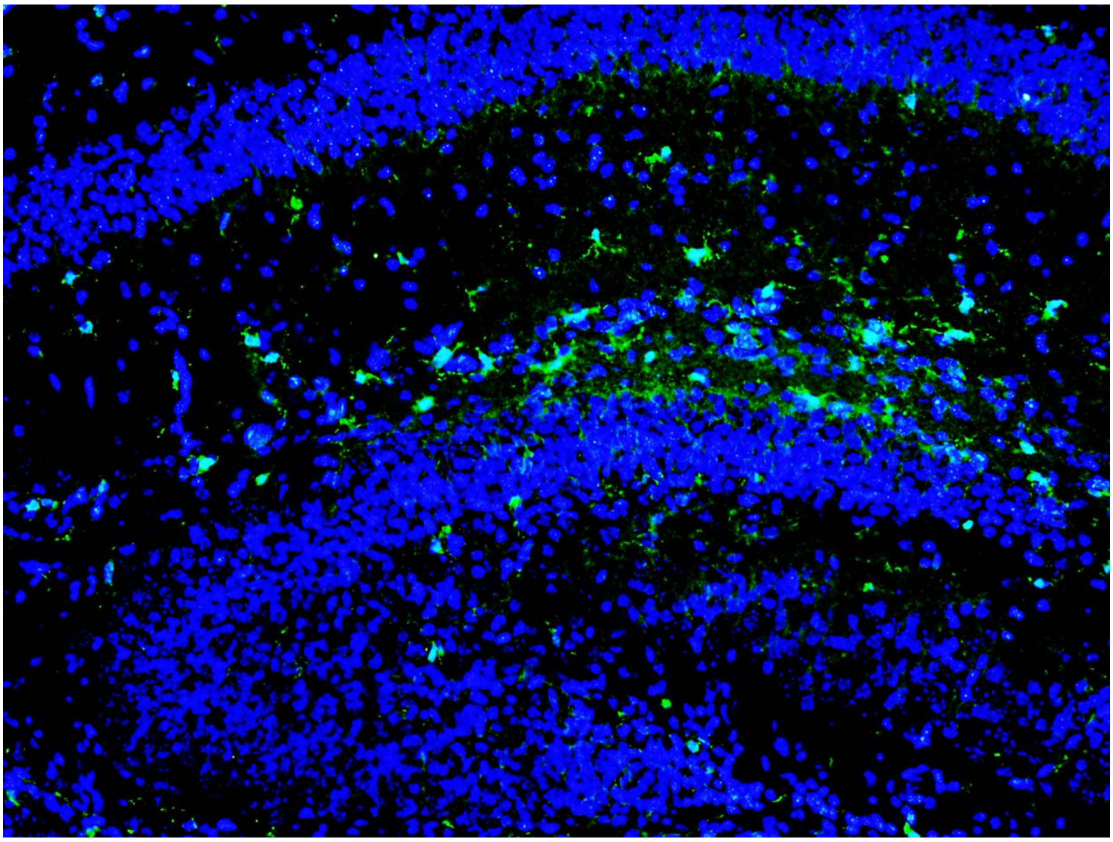

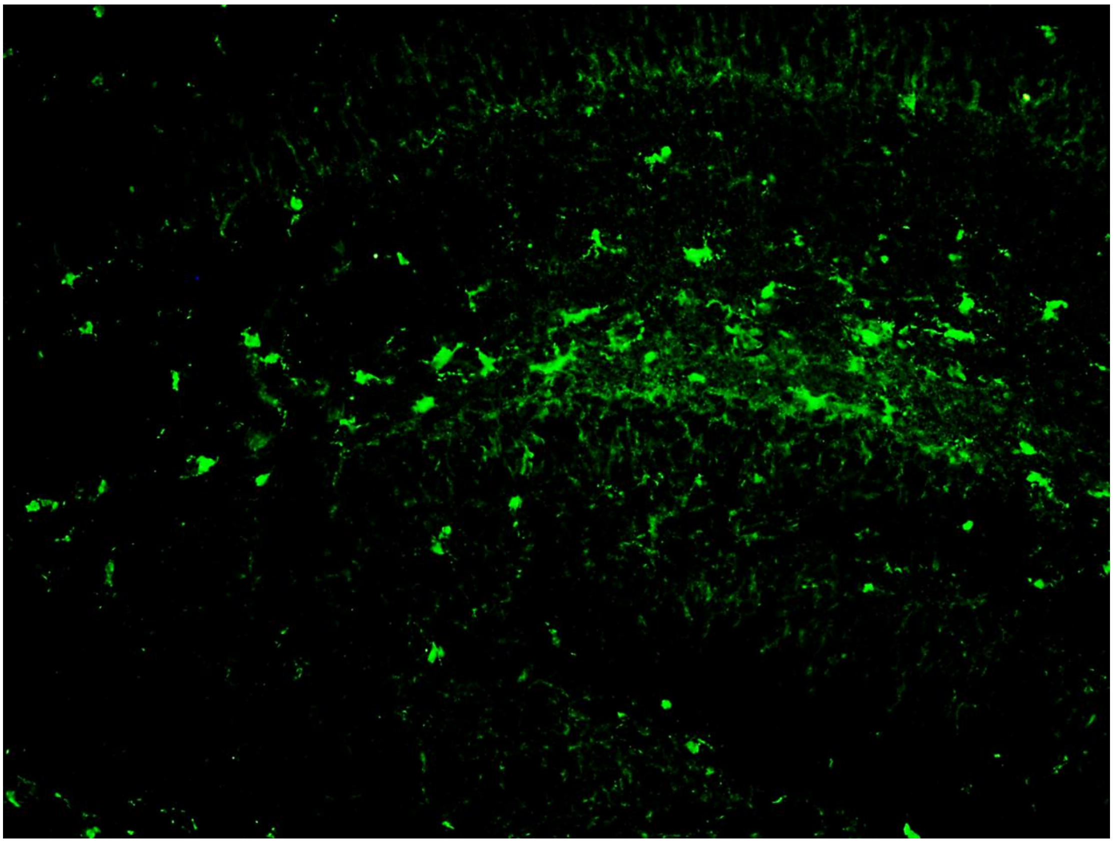

Figure2A Iba1 **PIA+FA**

Cortex

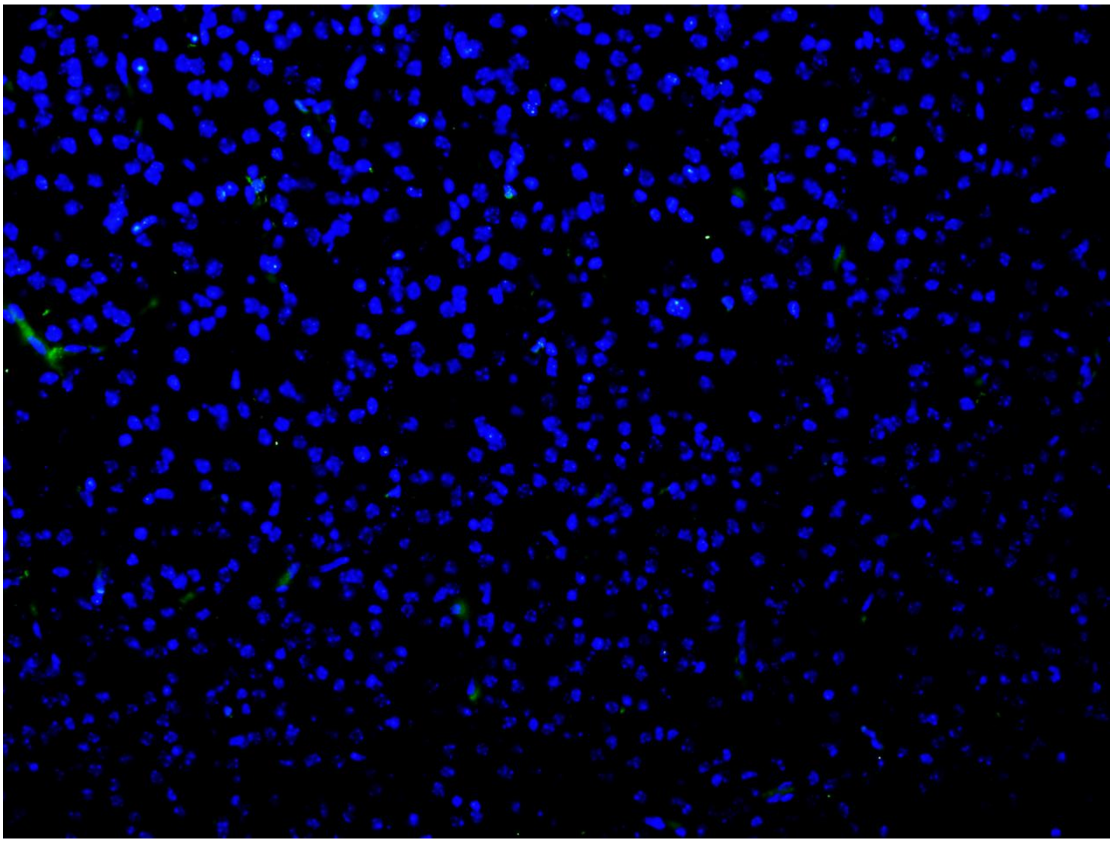

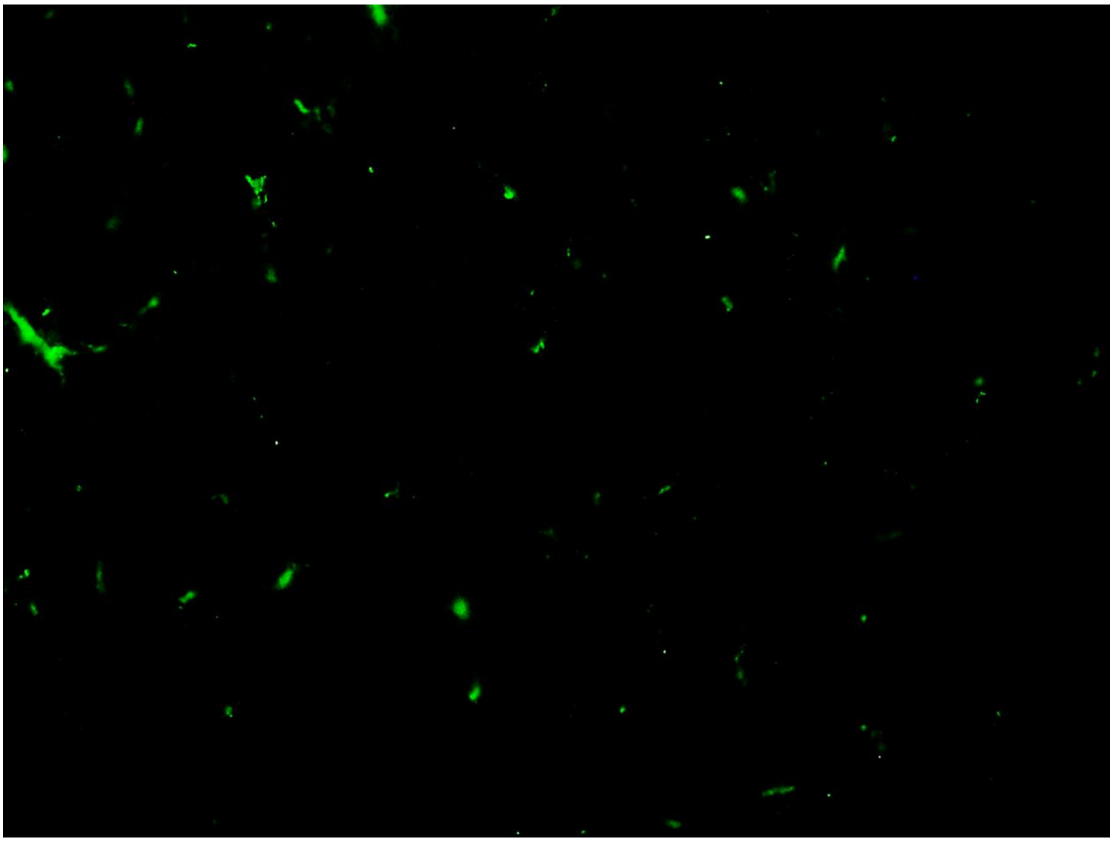

Hippocampus

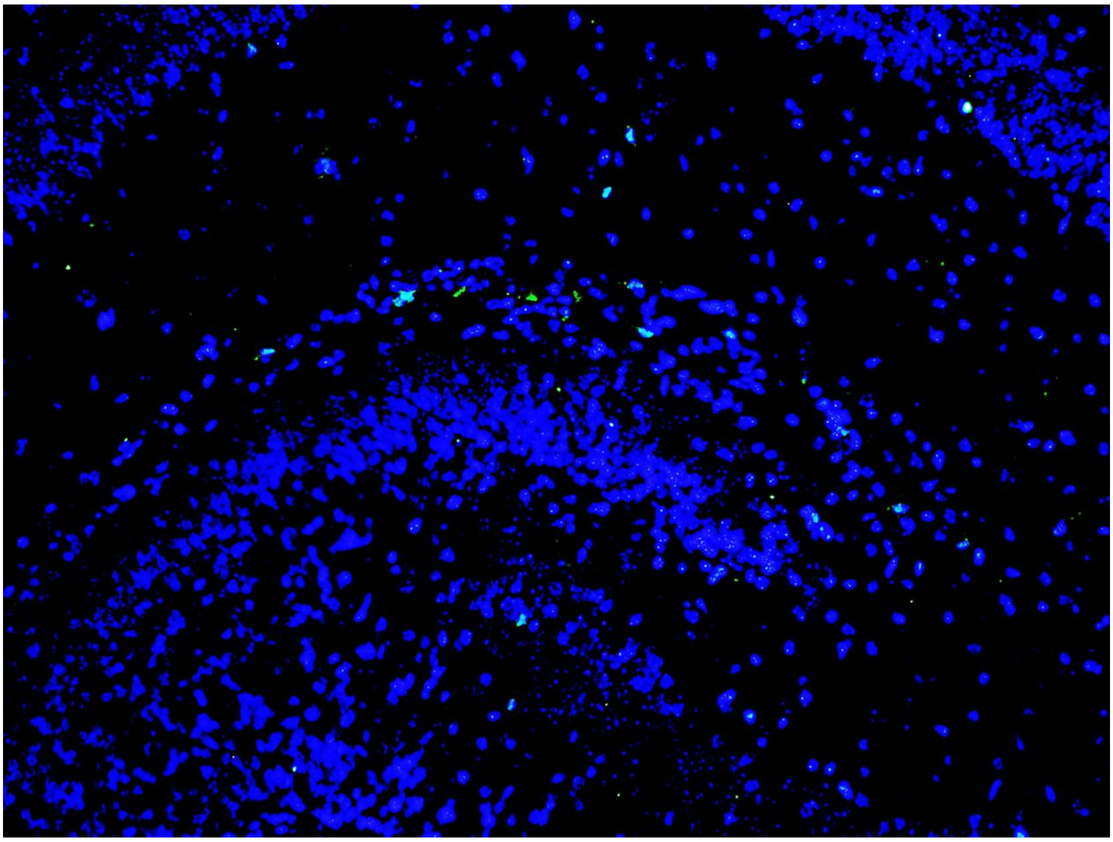

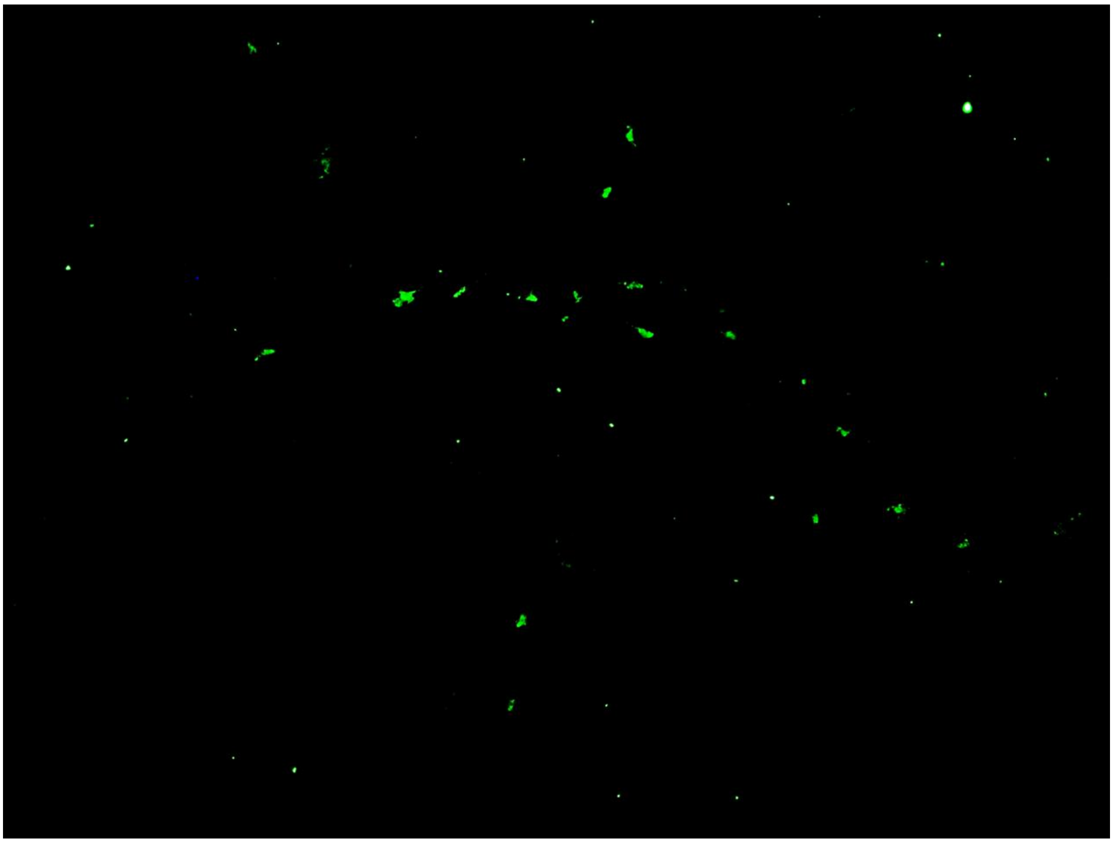

Figure3D **Control**

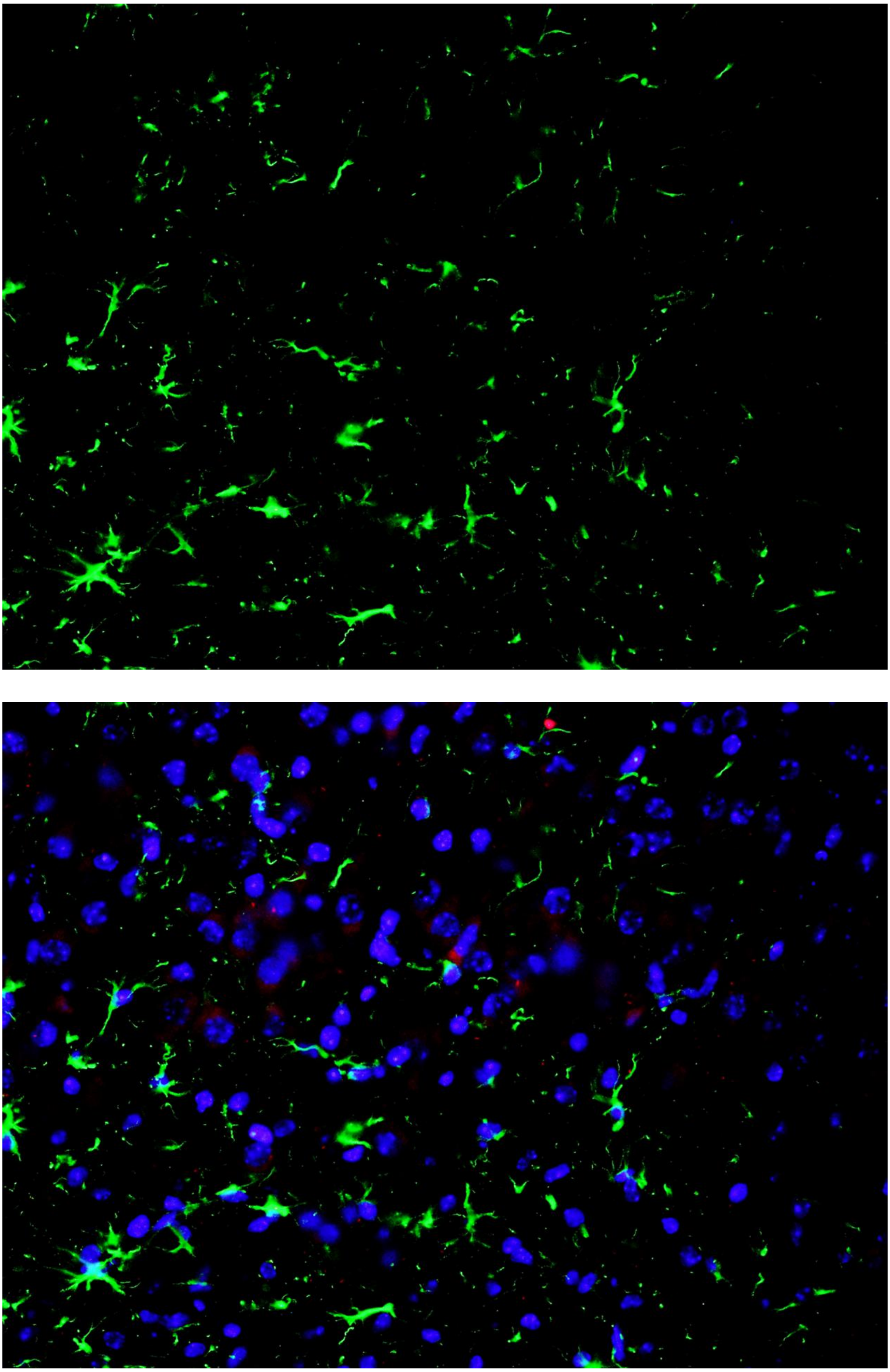

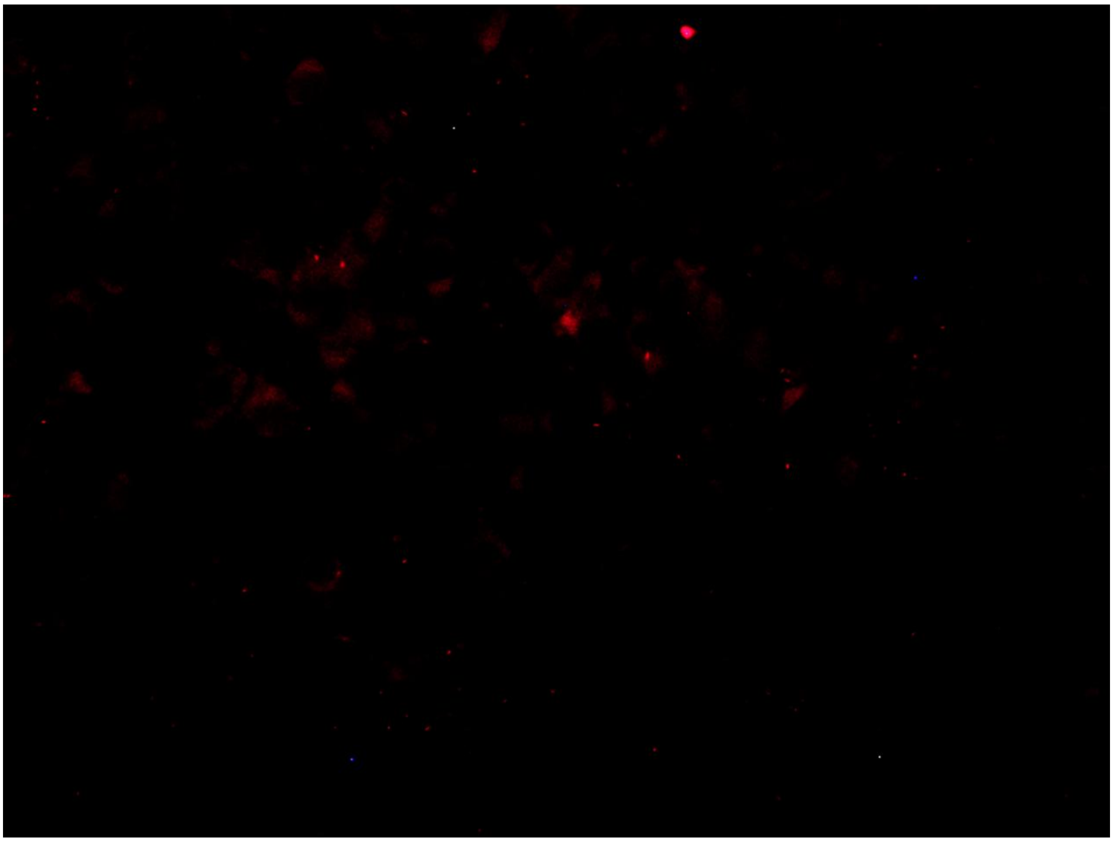

Figure3D **PIA**

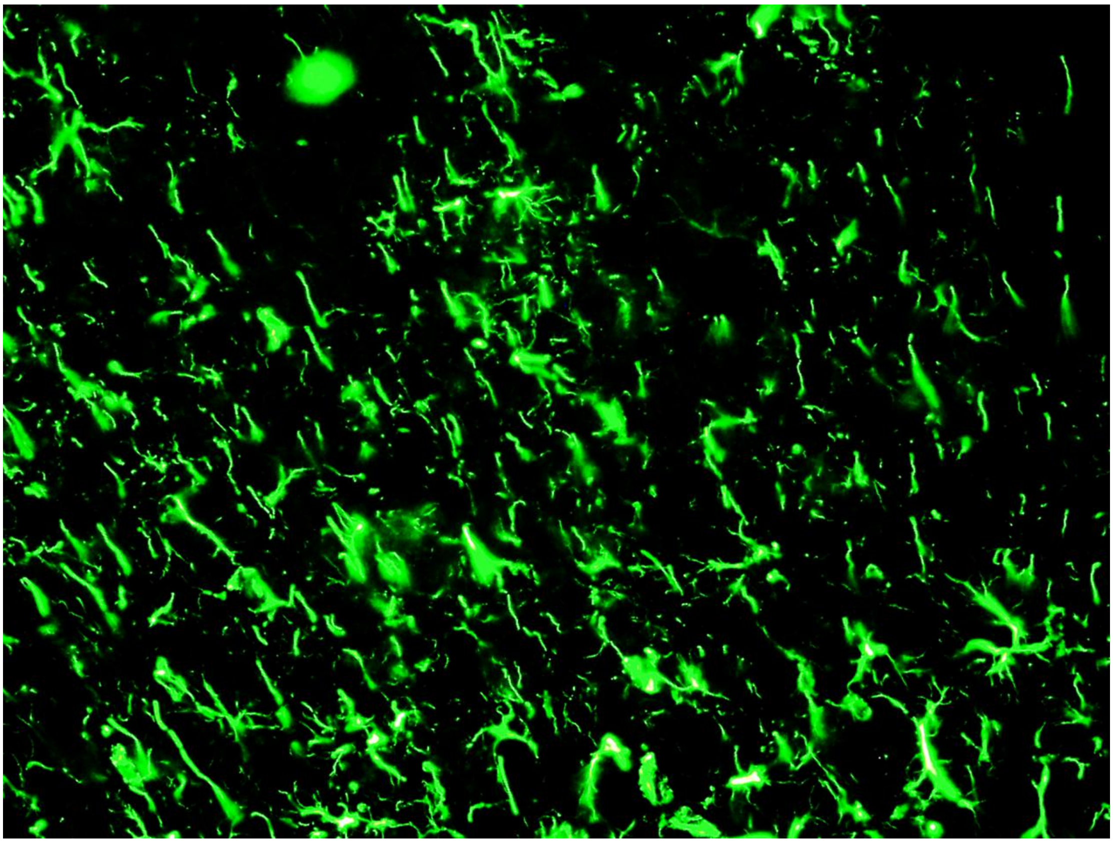

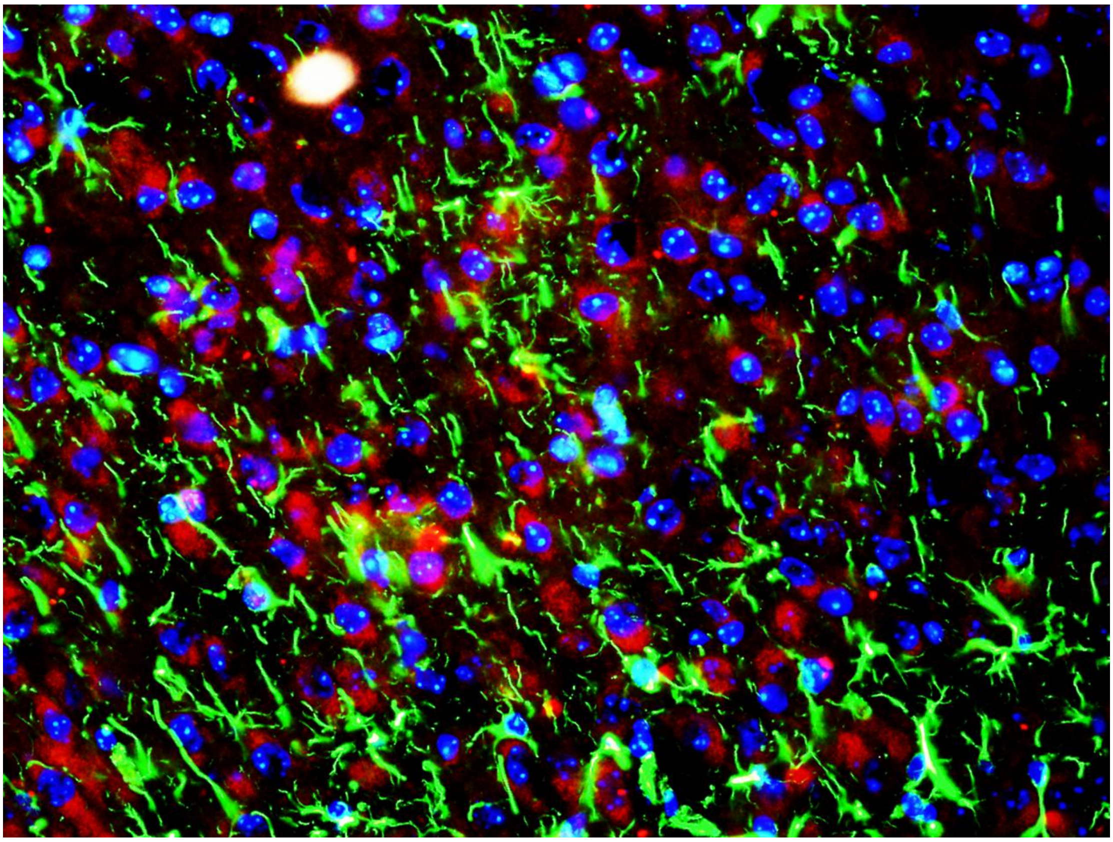

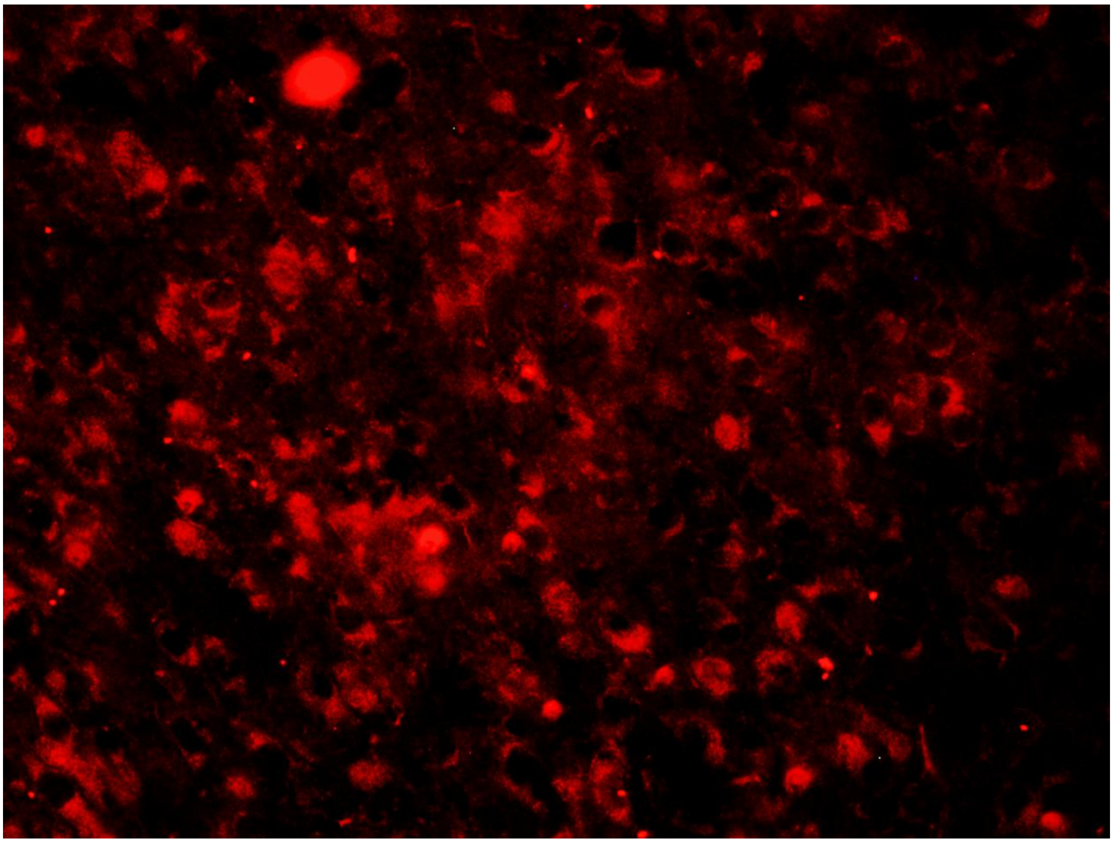

Figure3D **PIA+FA**

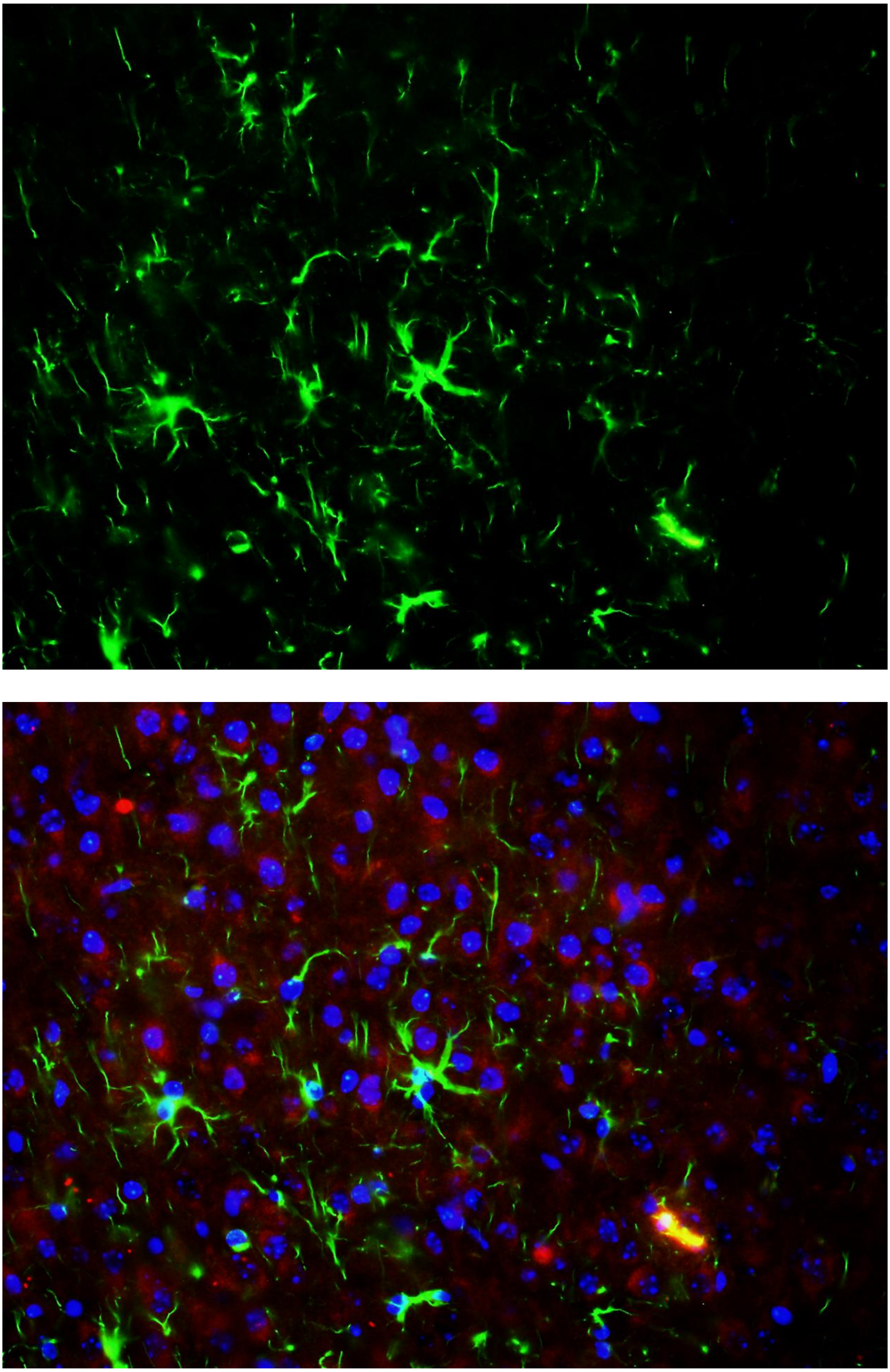

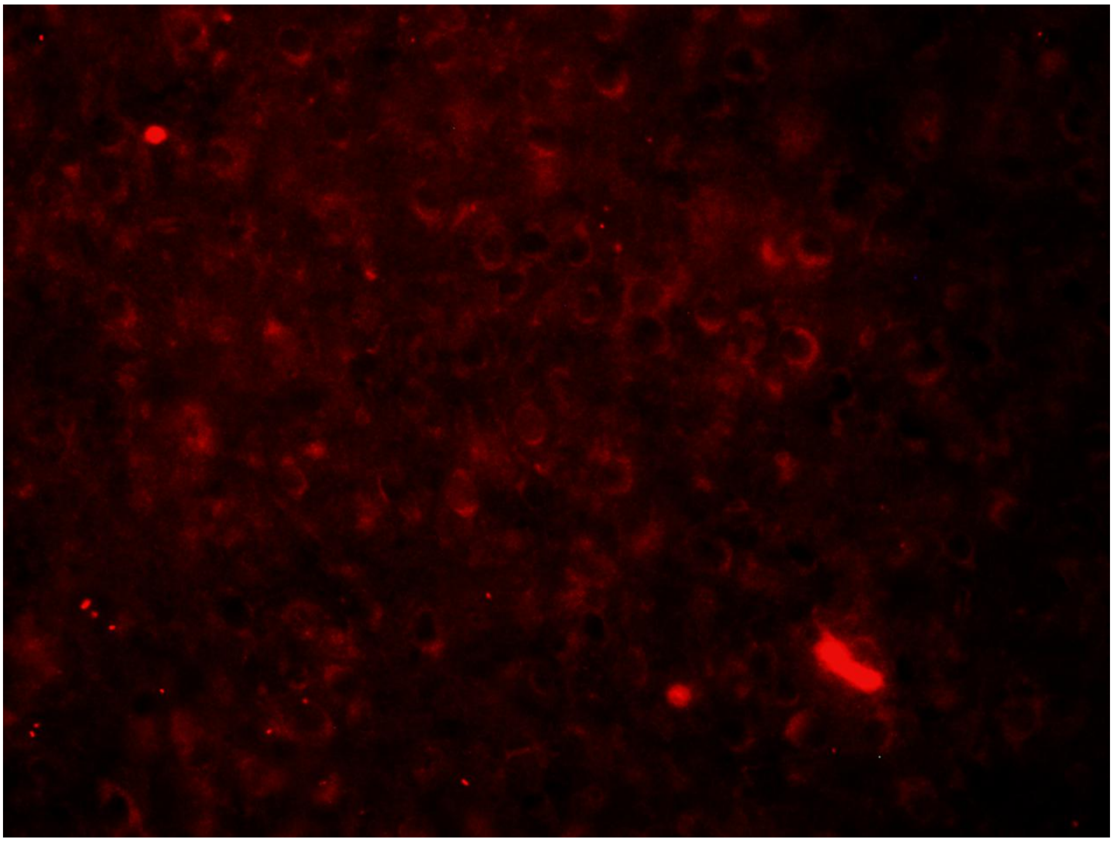

Figure3E **Control**

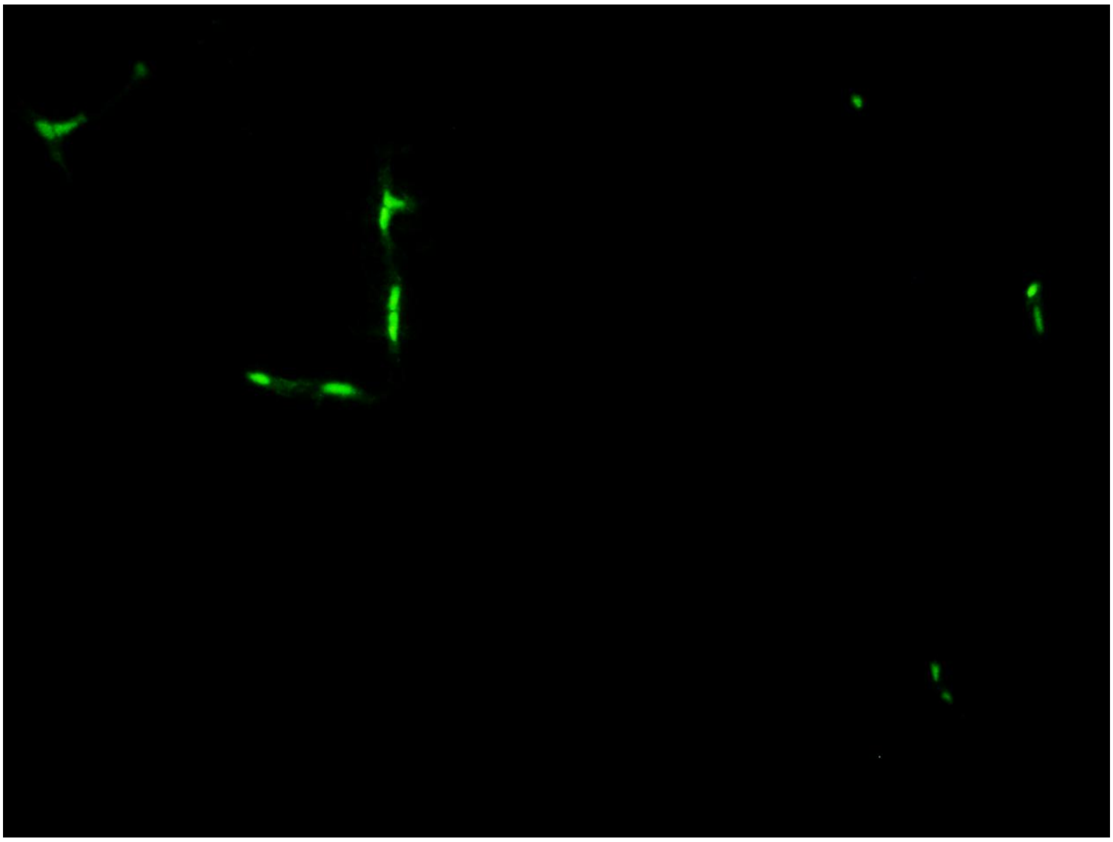

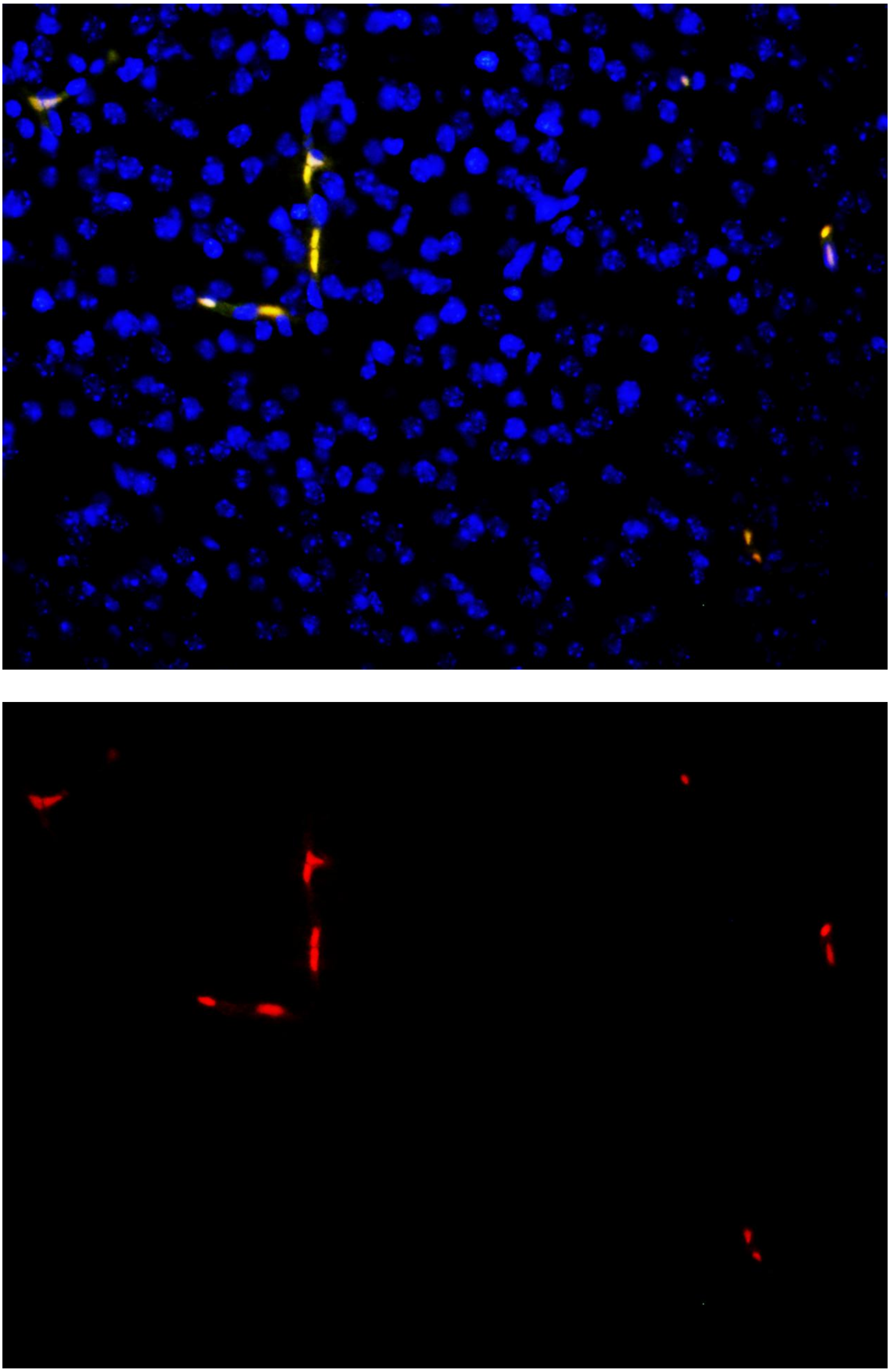

Figure3E **PIA**

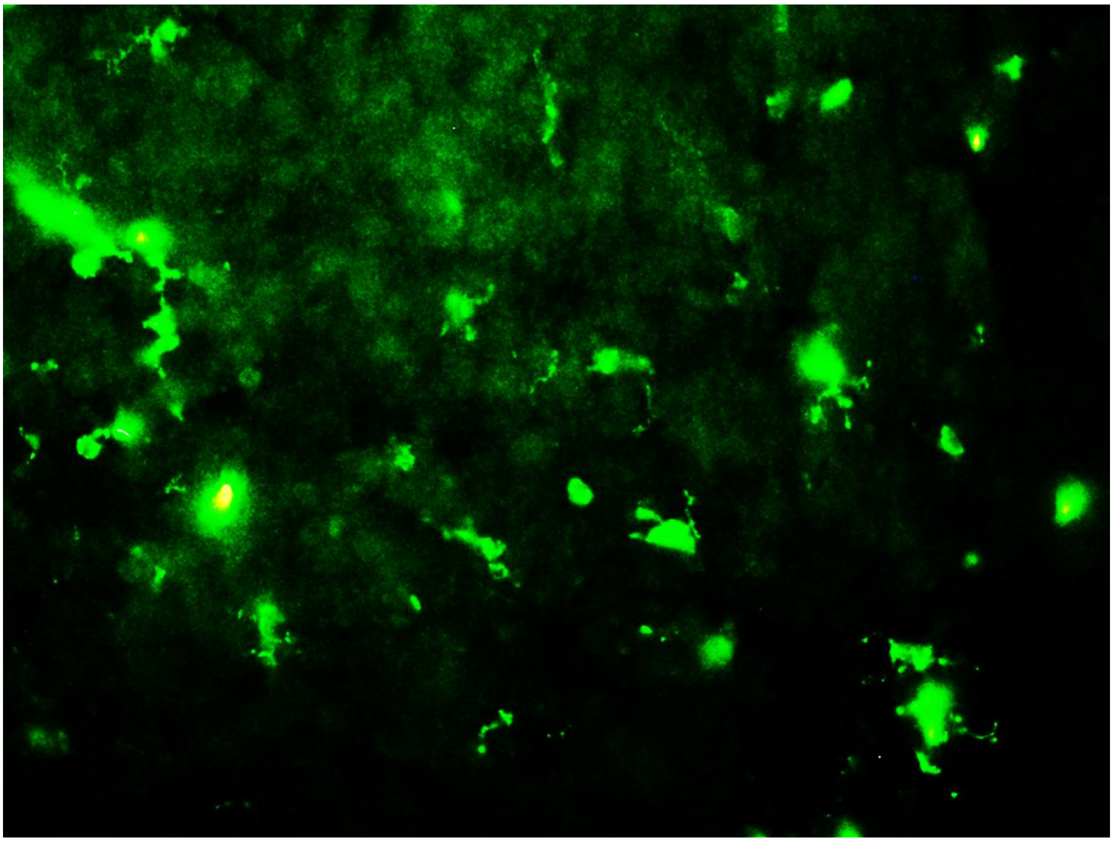

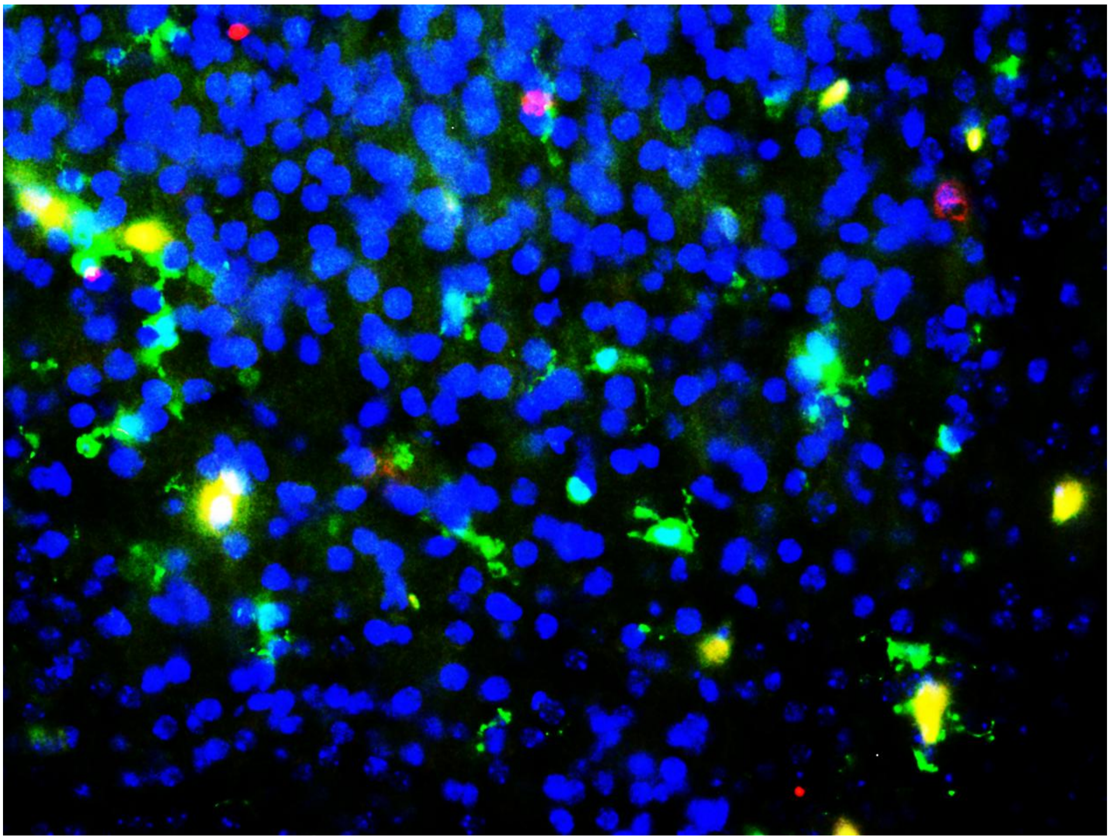

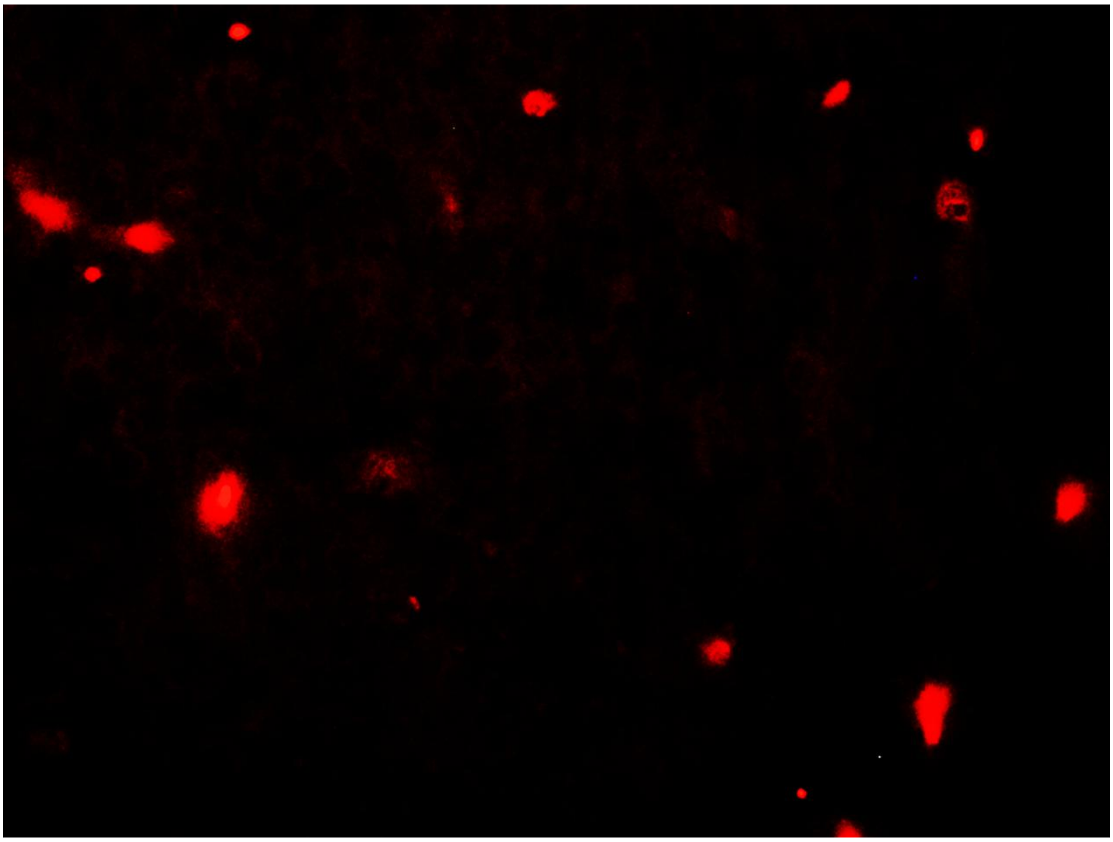

Figure3E **PIA+FA**

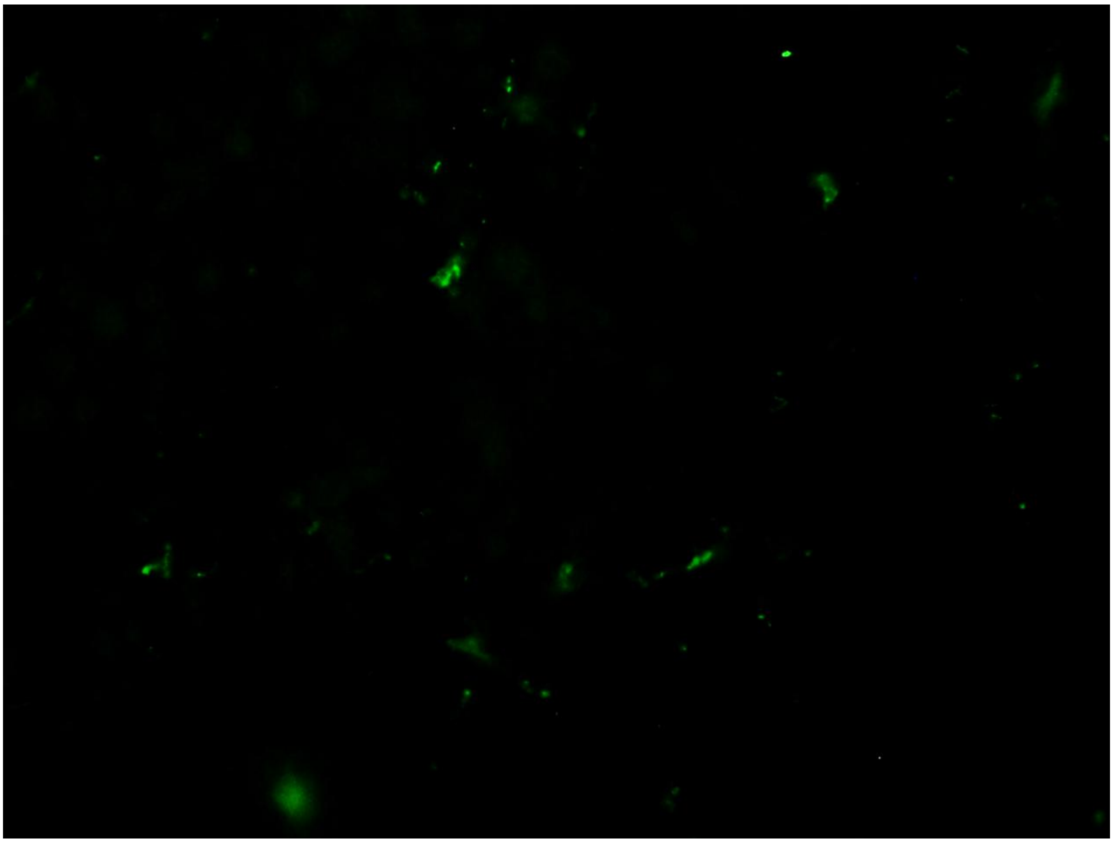

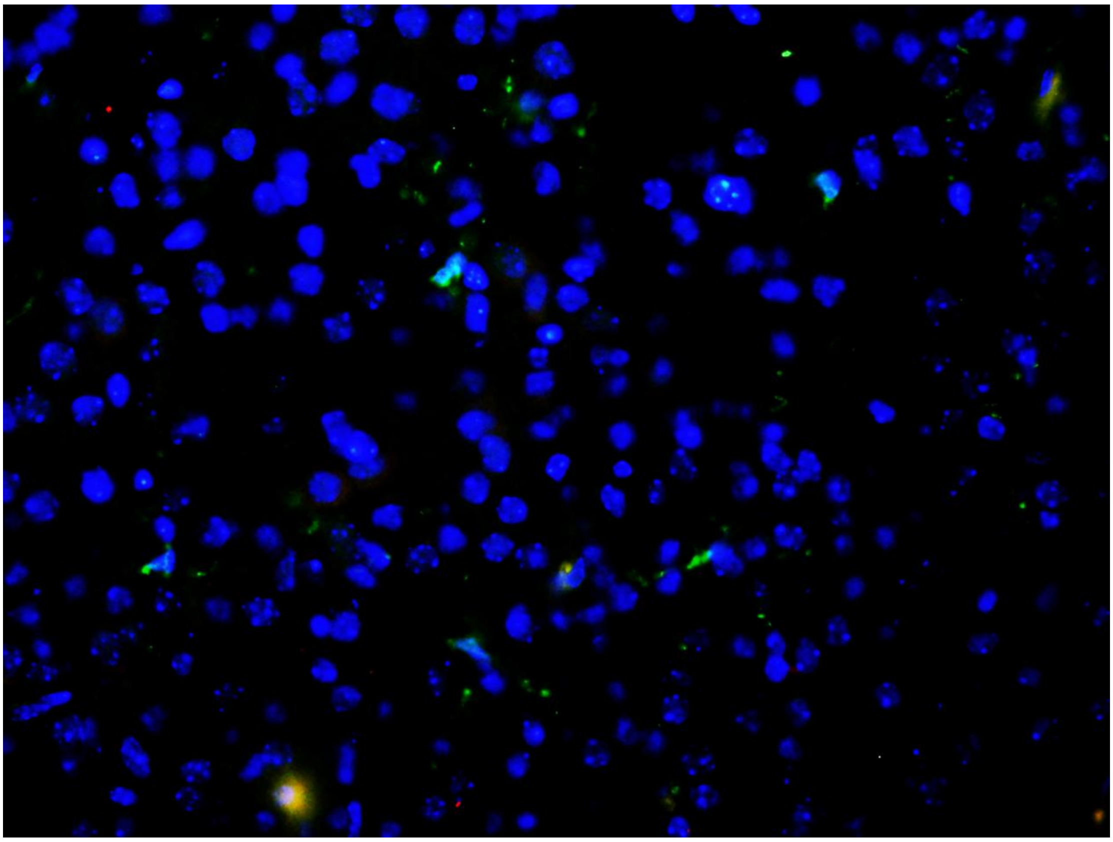

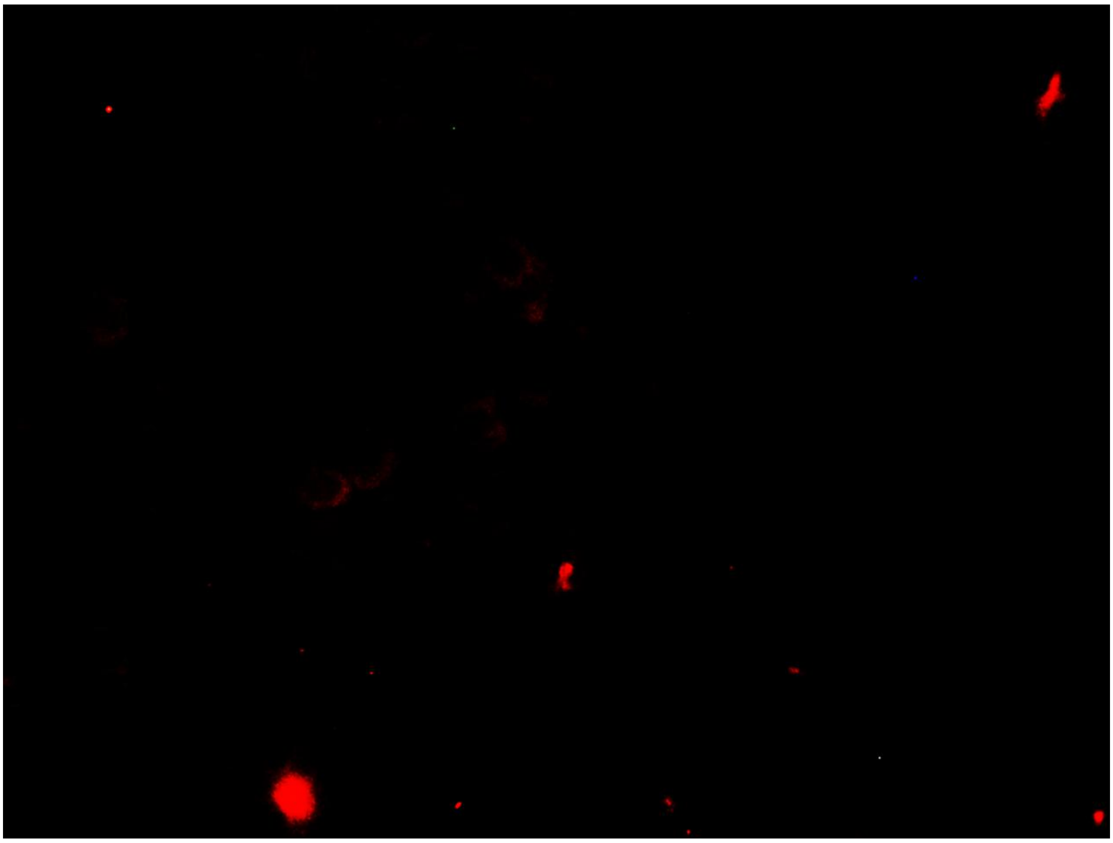

Figure4A **Astrocyte**

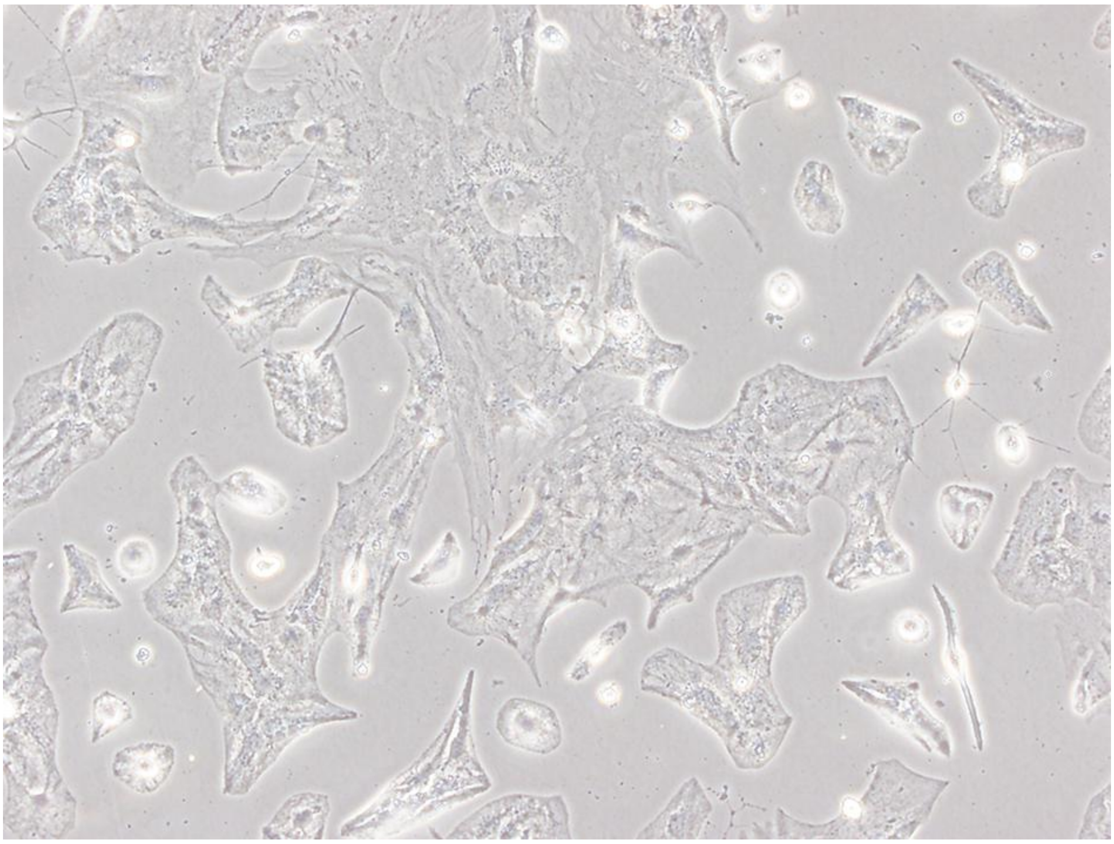

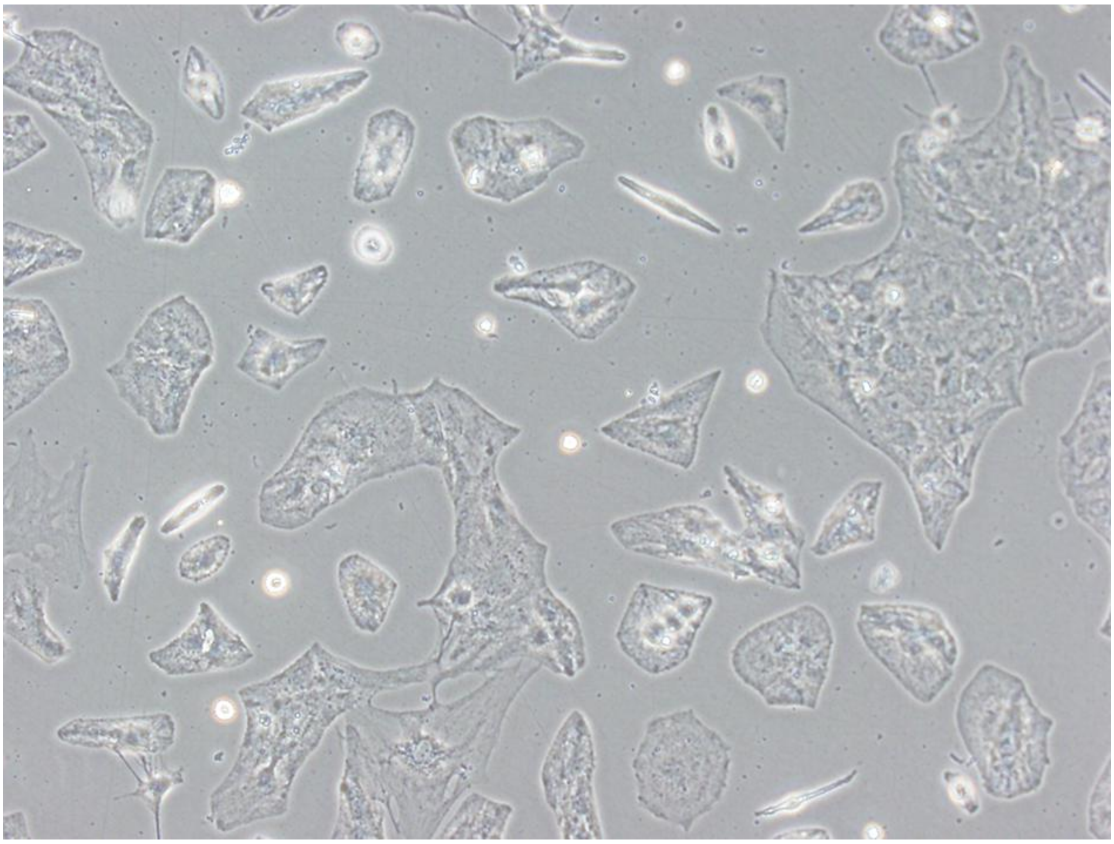

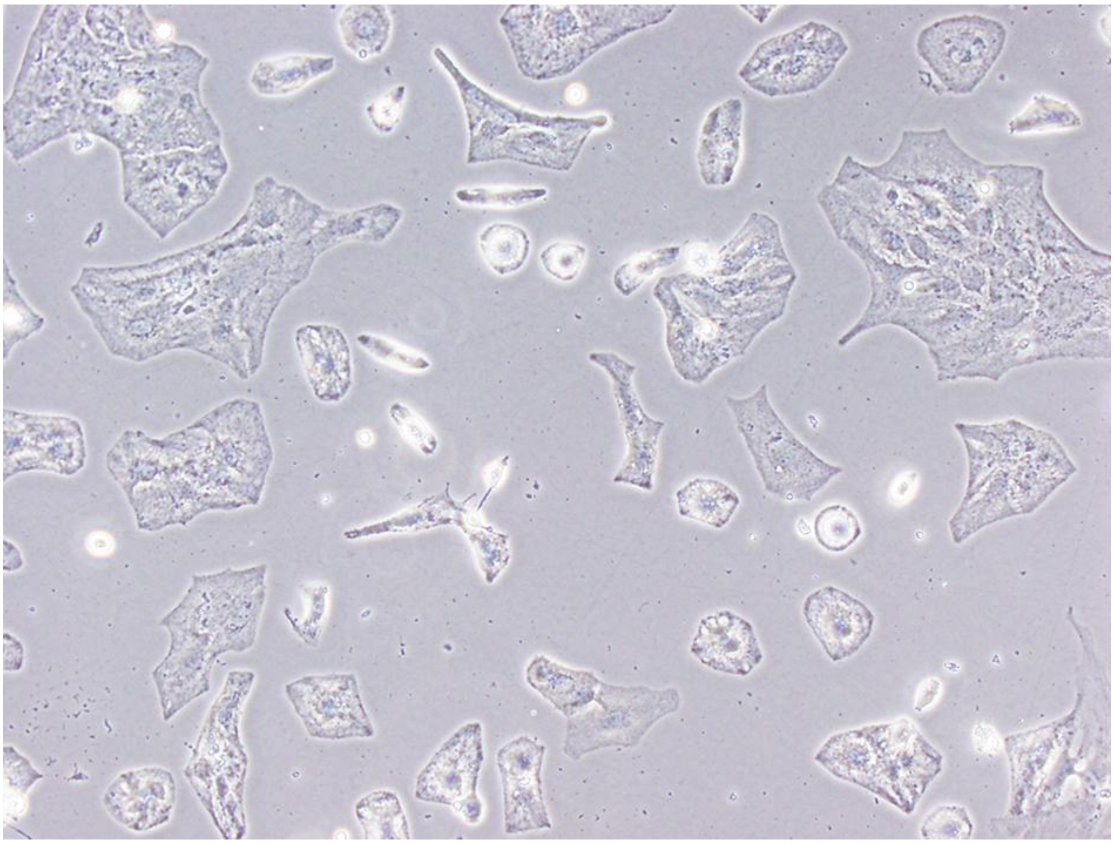

Figure4A **Microglia**

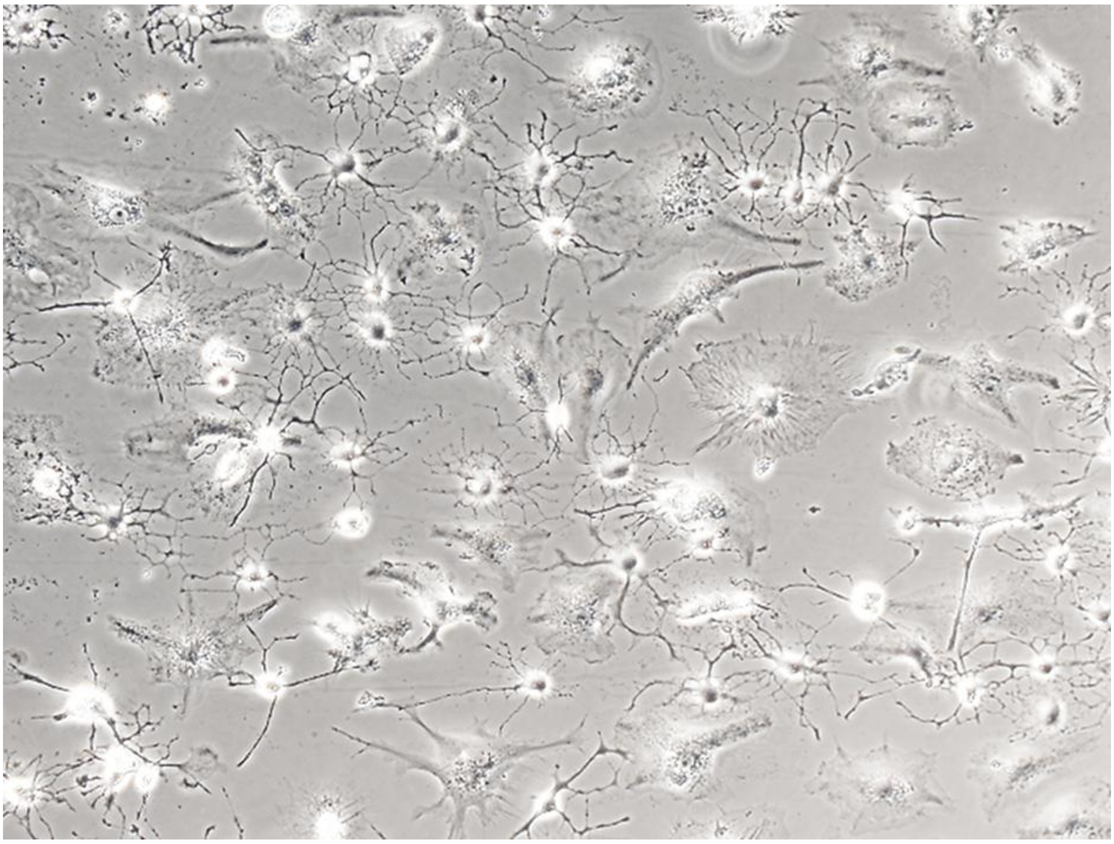

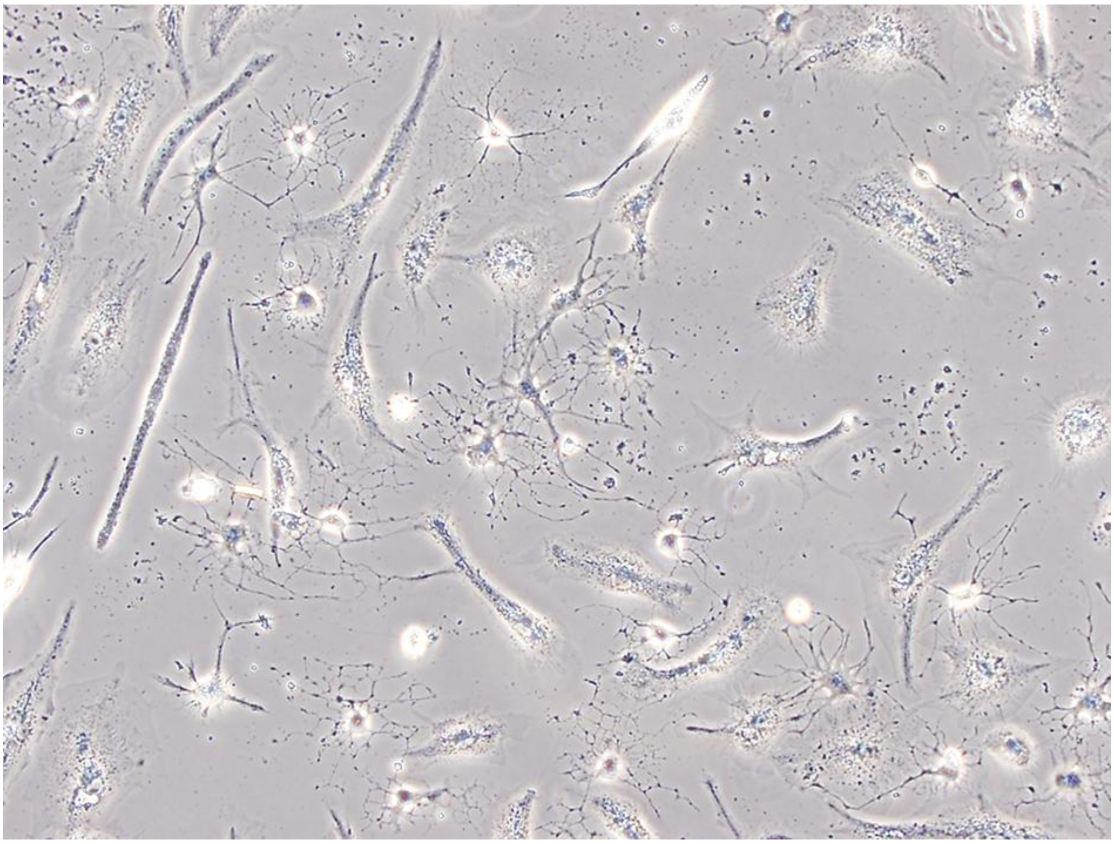

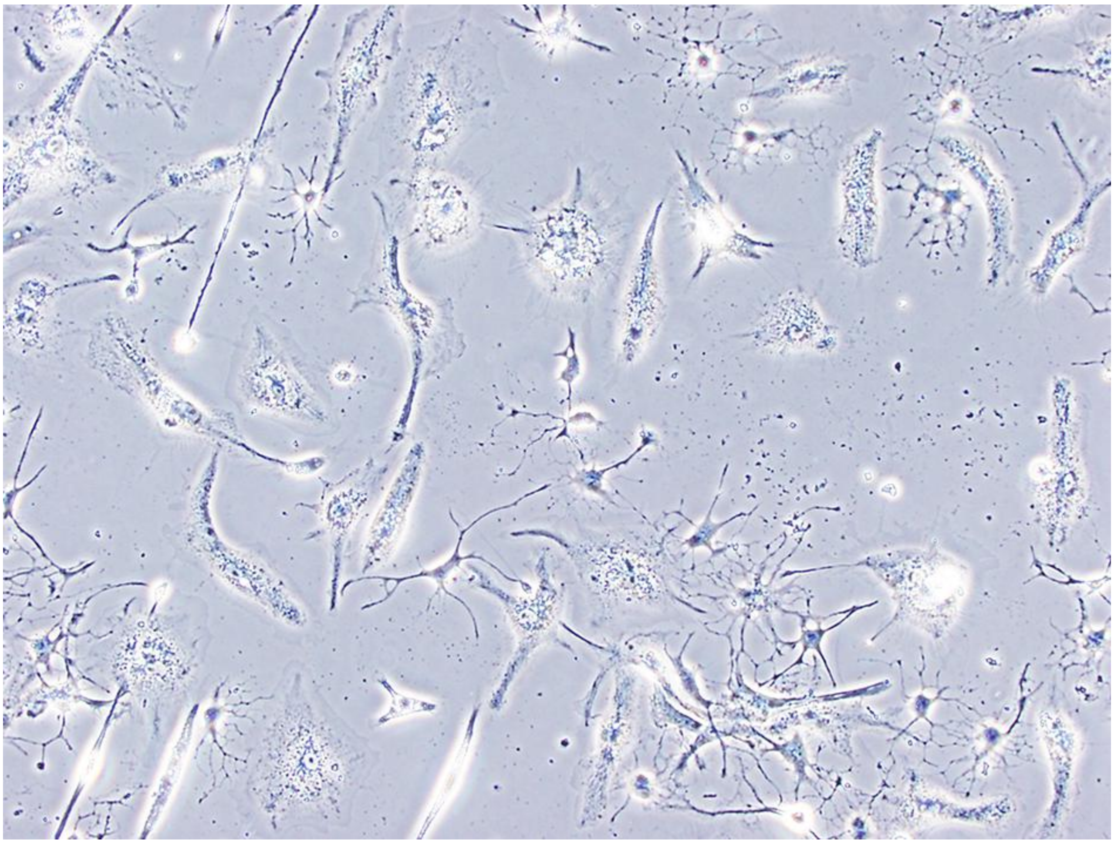

Figure4A GFAP

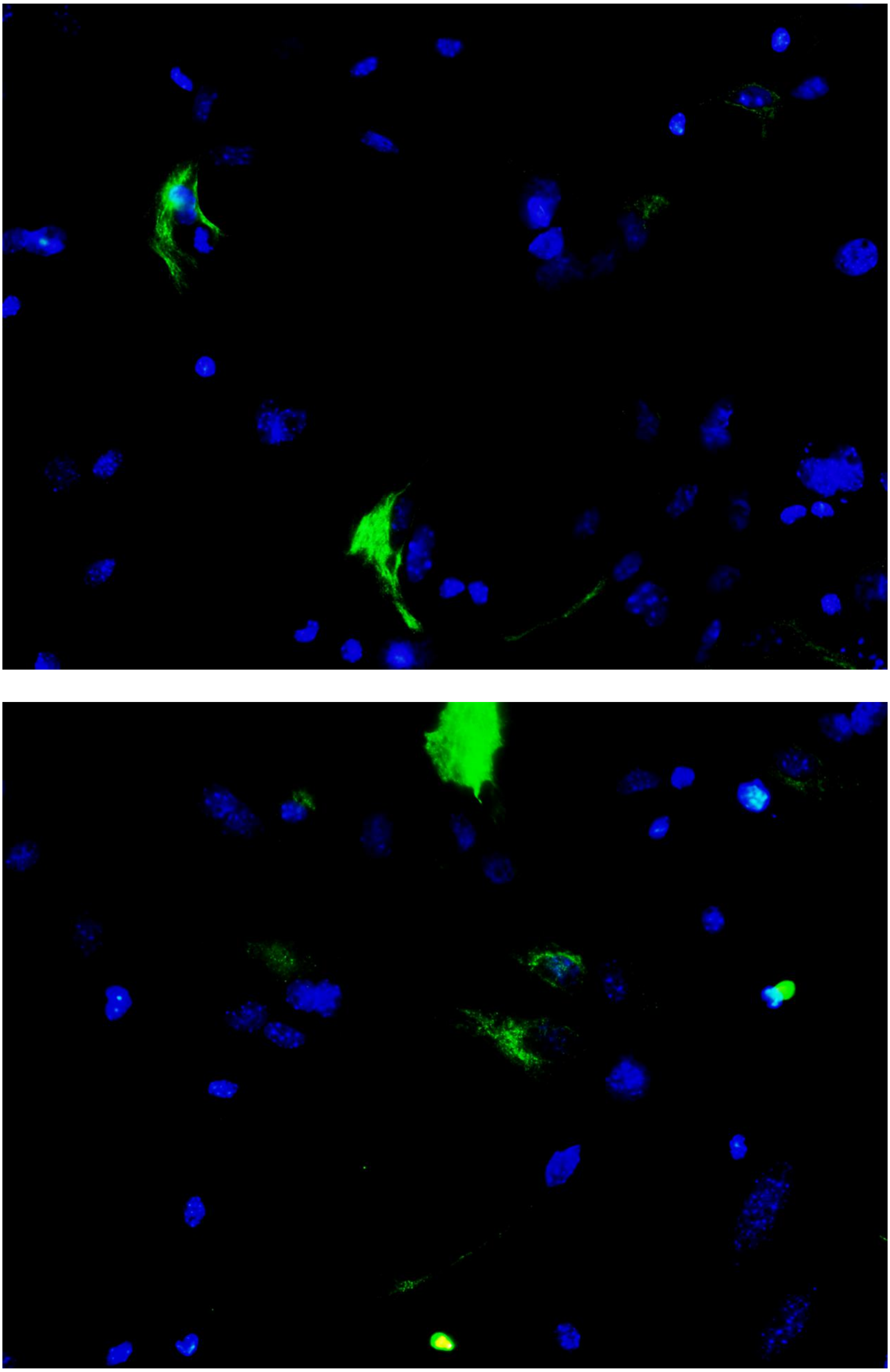

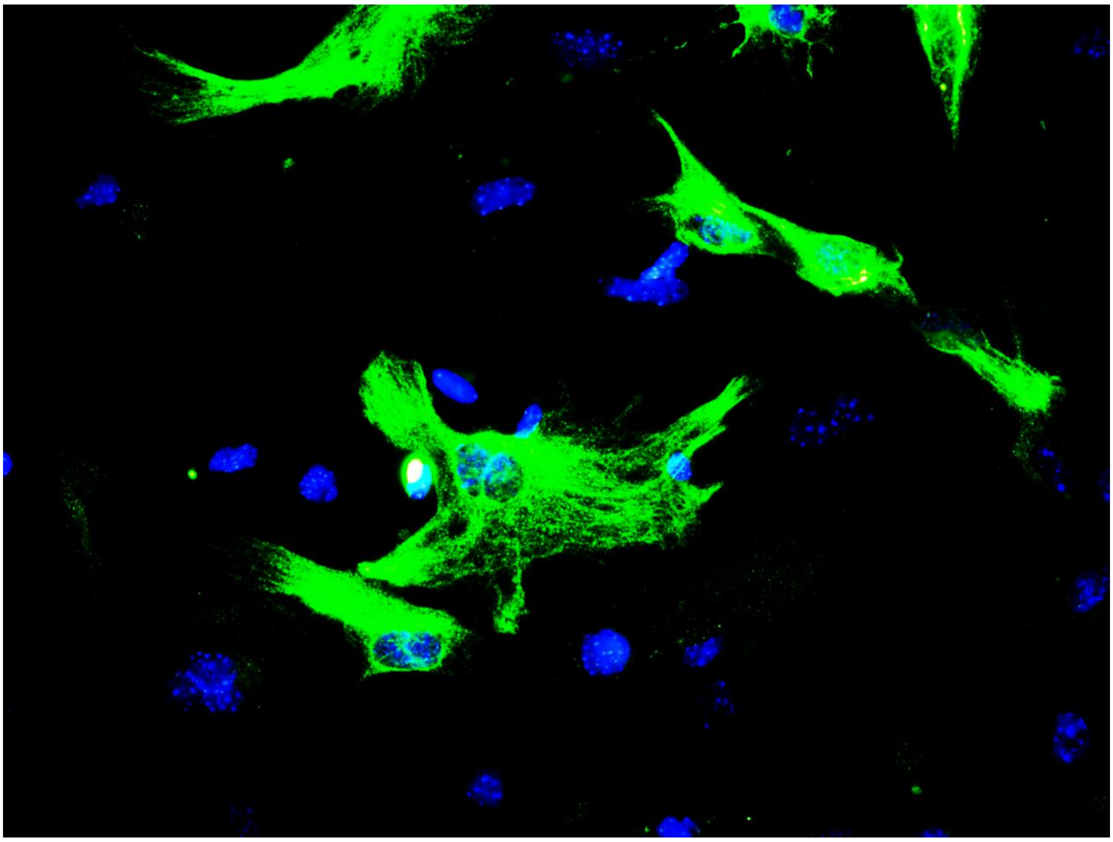

Figure4A Iba1

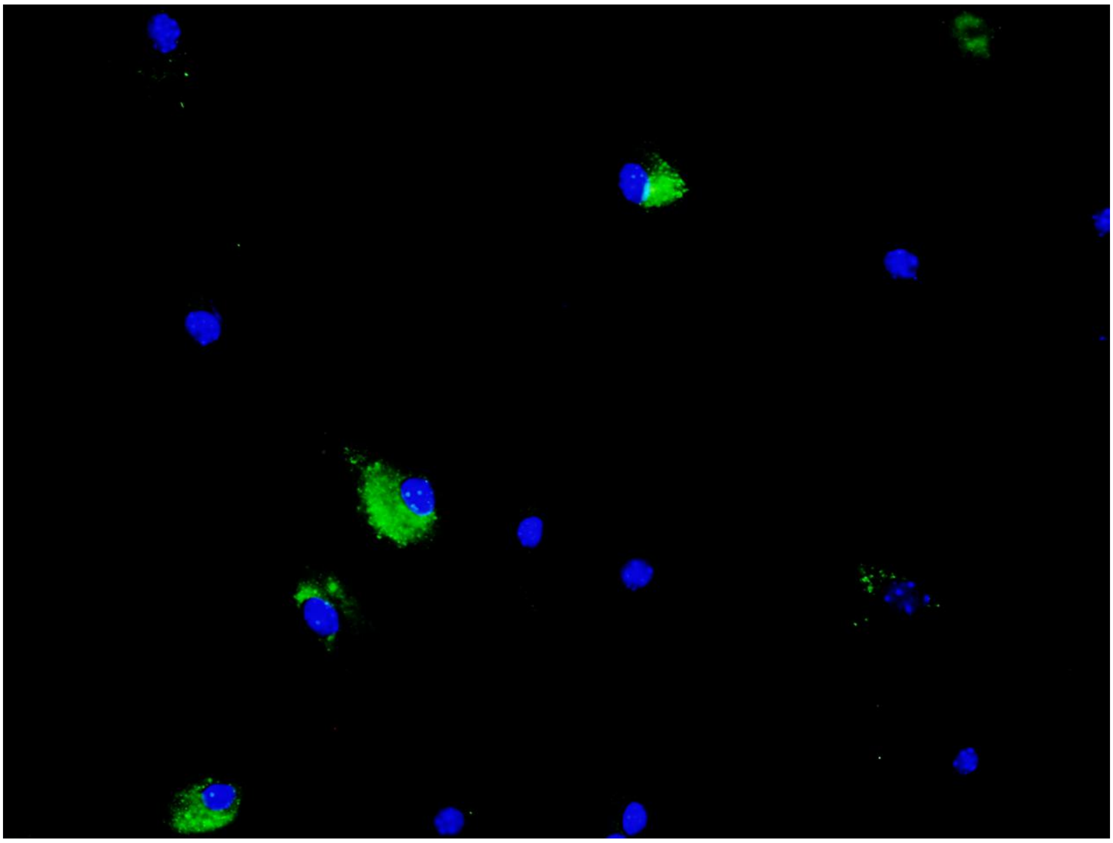

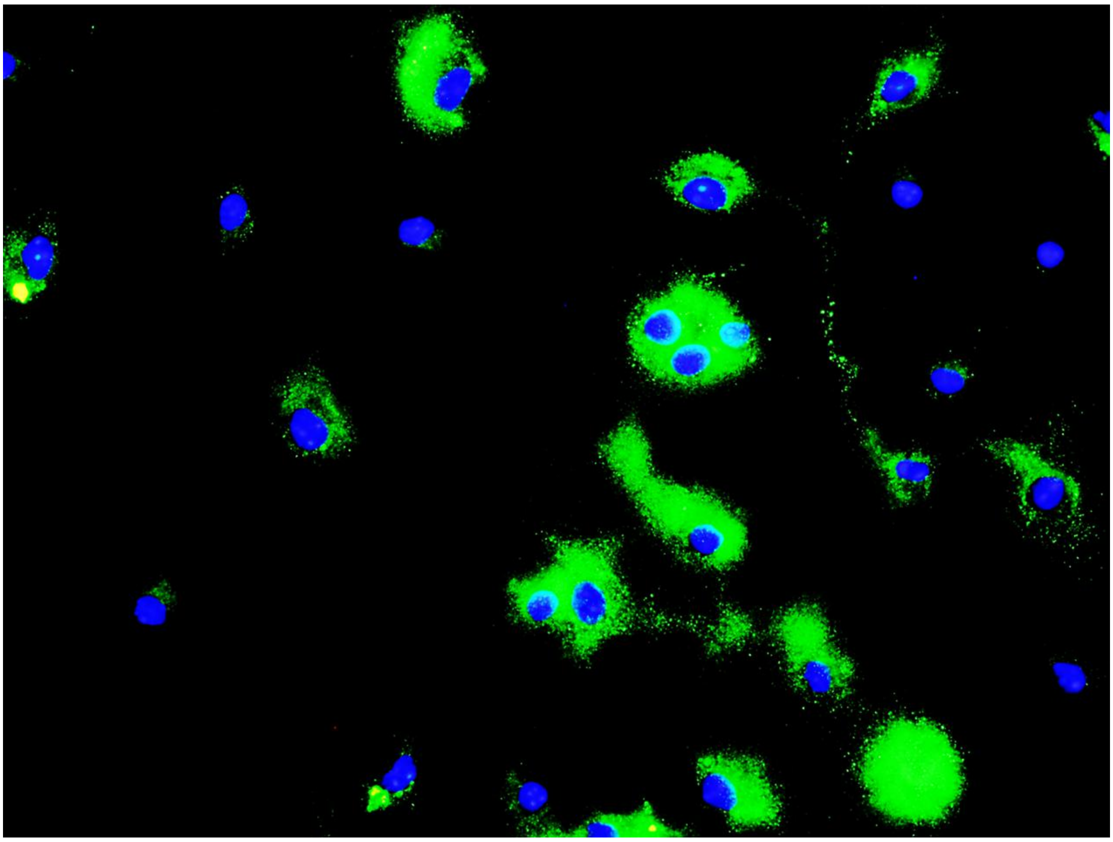

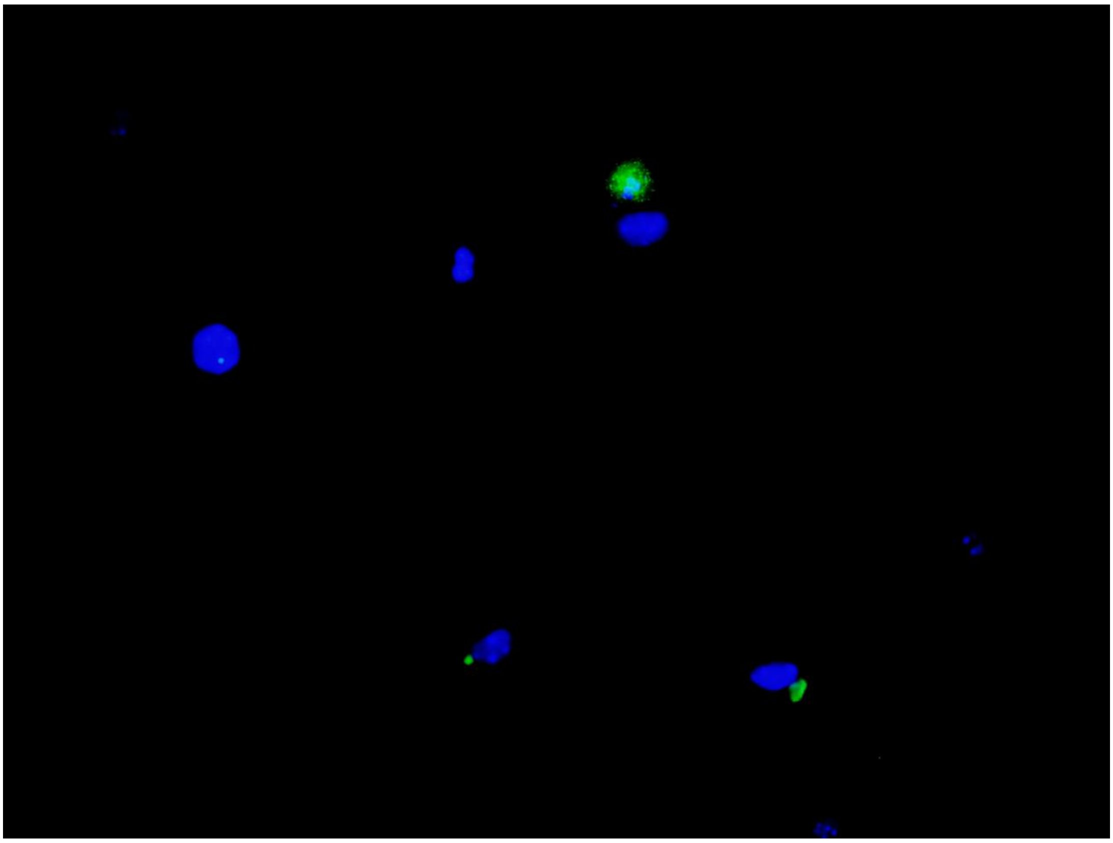

Figure6G **Control**

Cortex

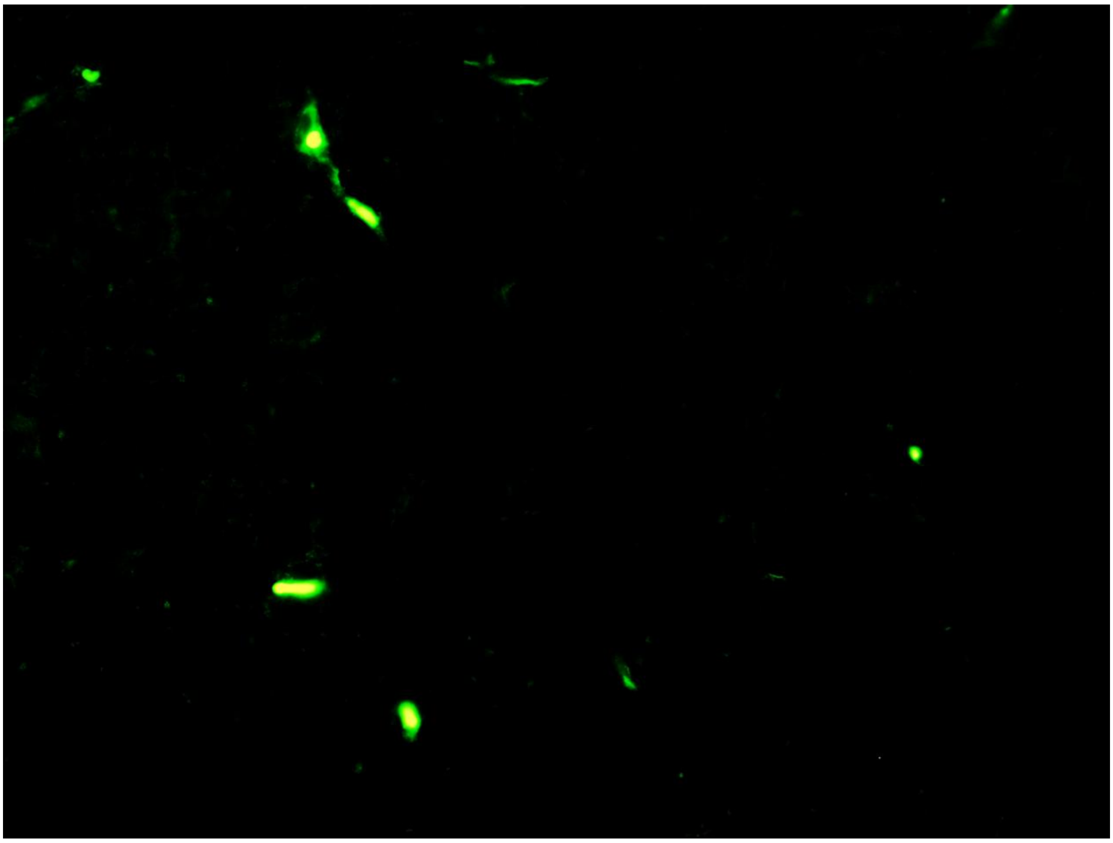

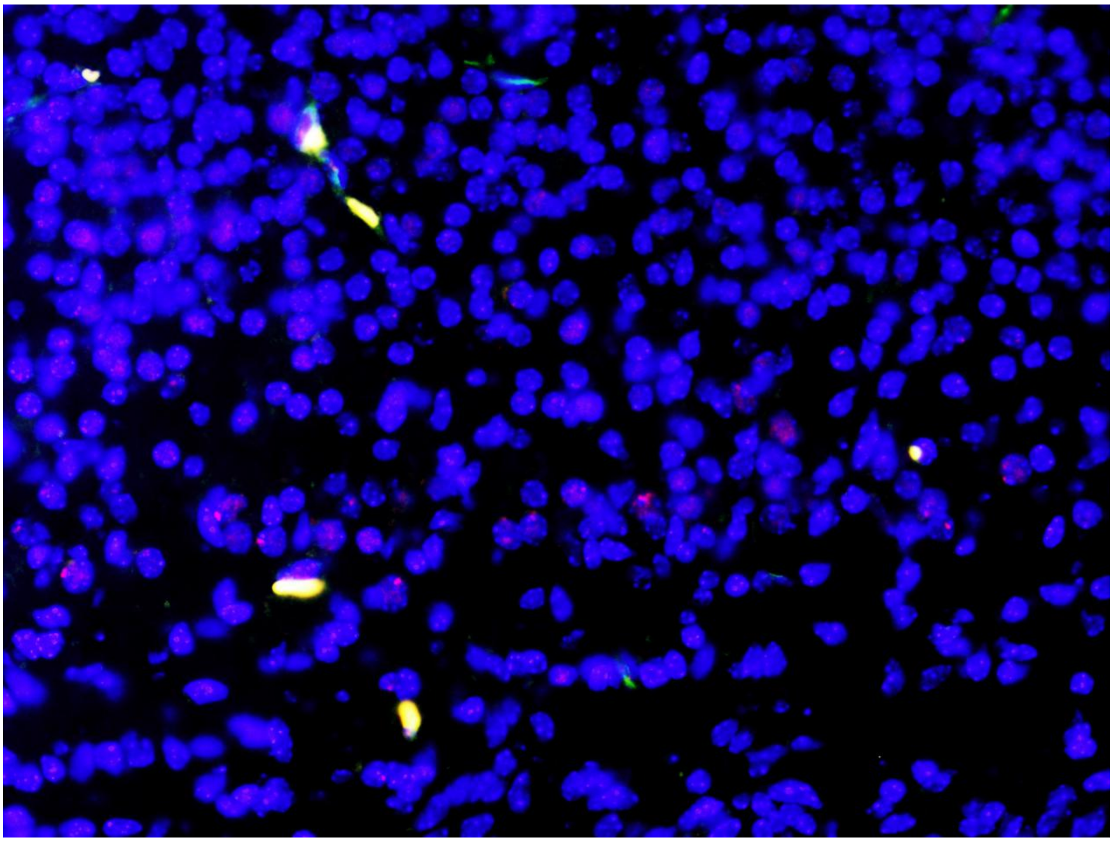

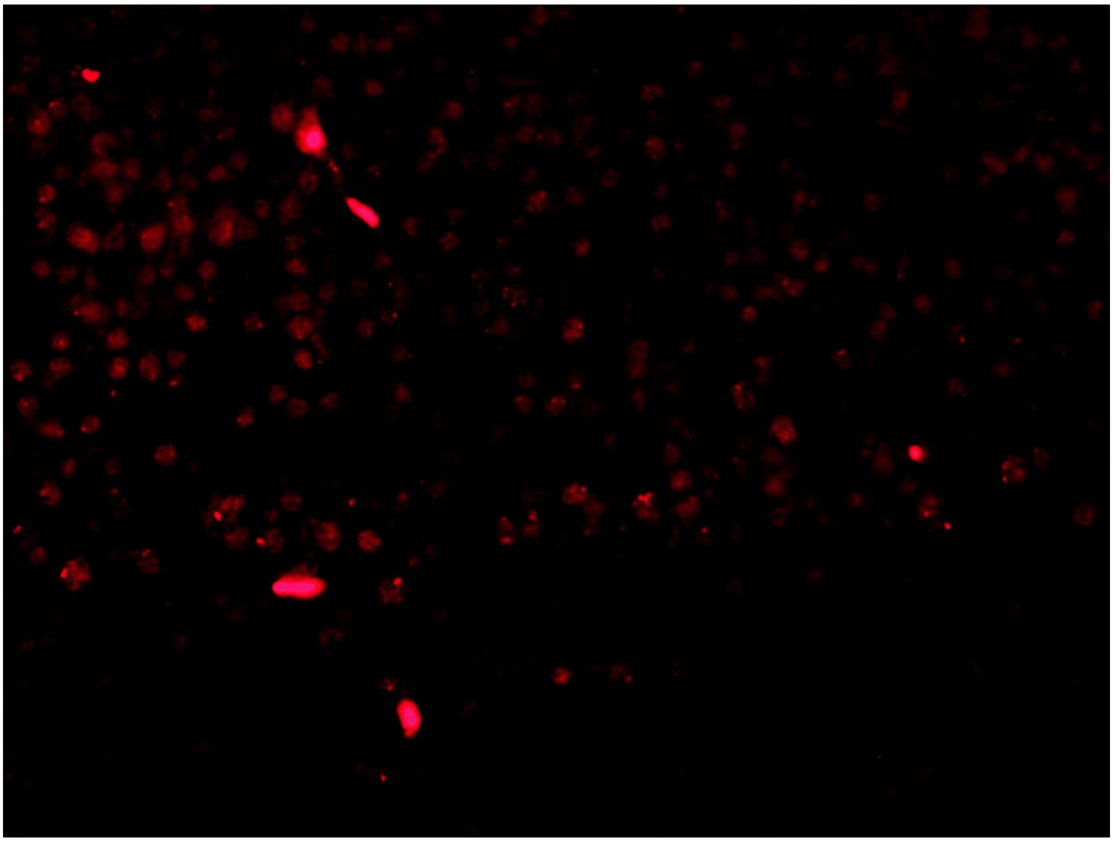

Hippocampus

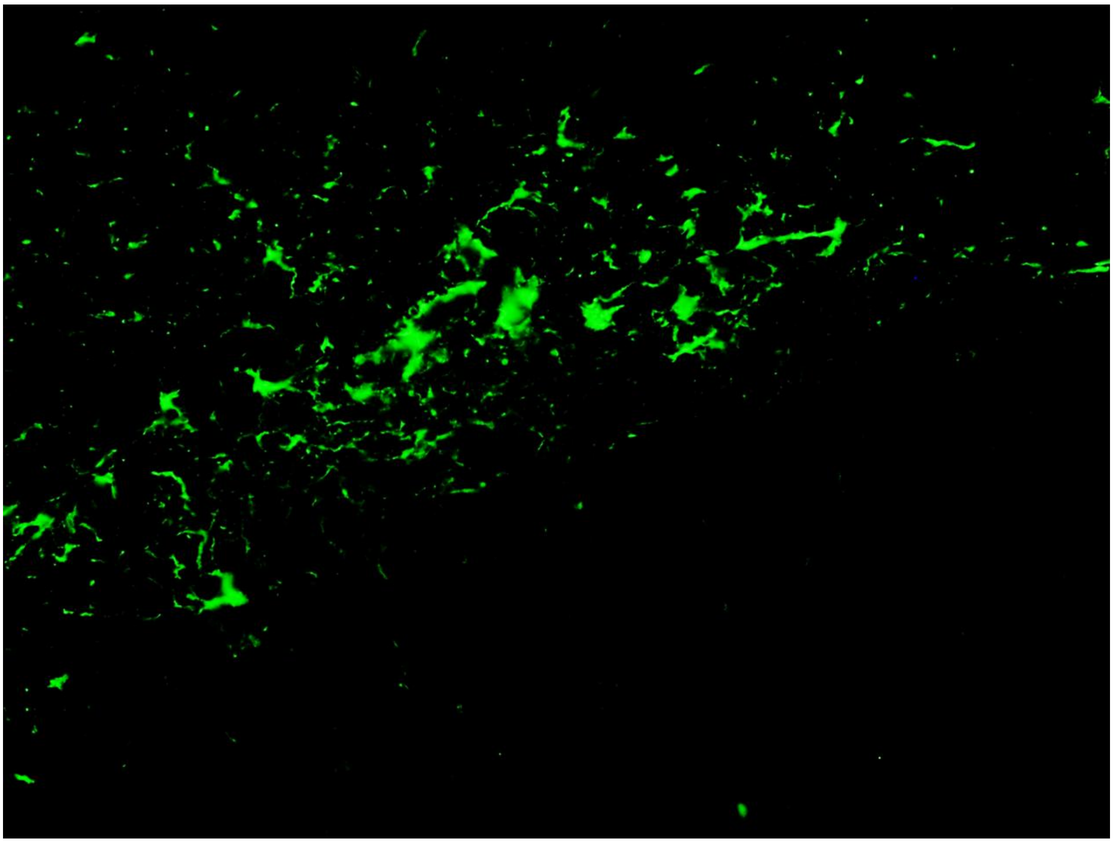

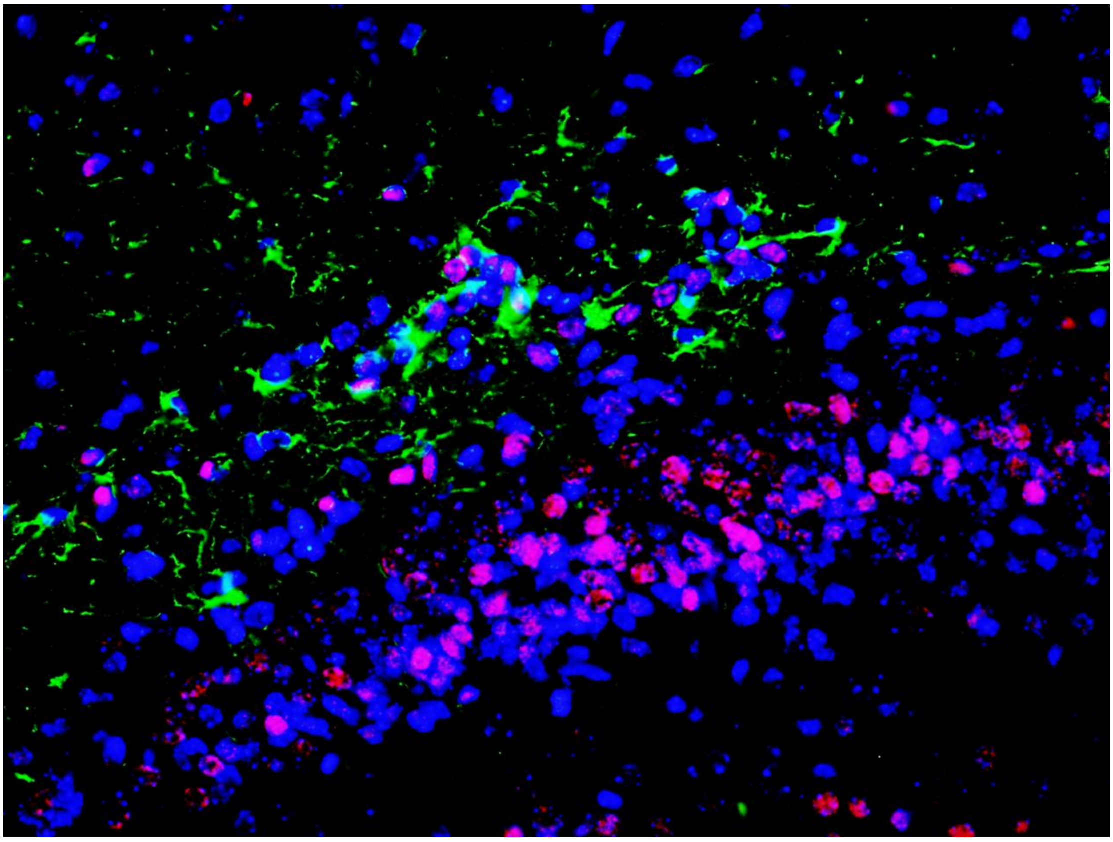

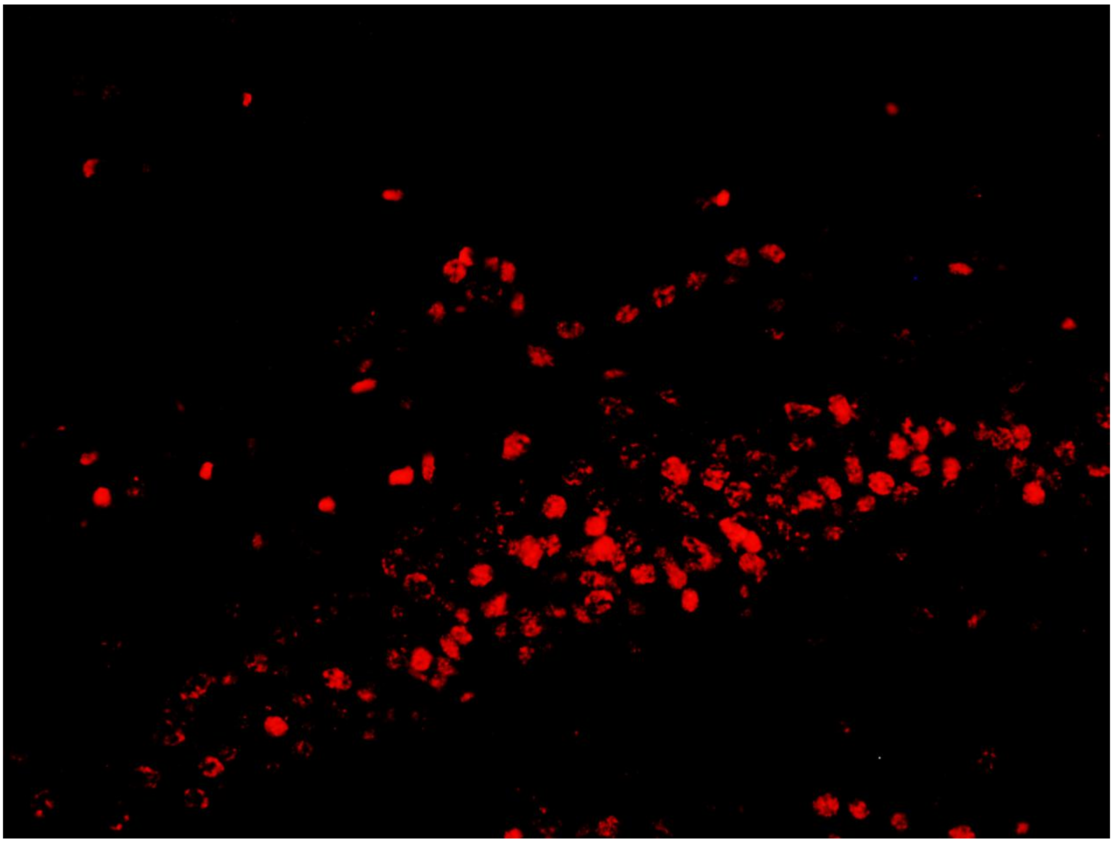

Figure6G **PIA**

Cortex

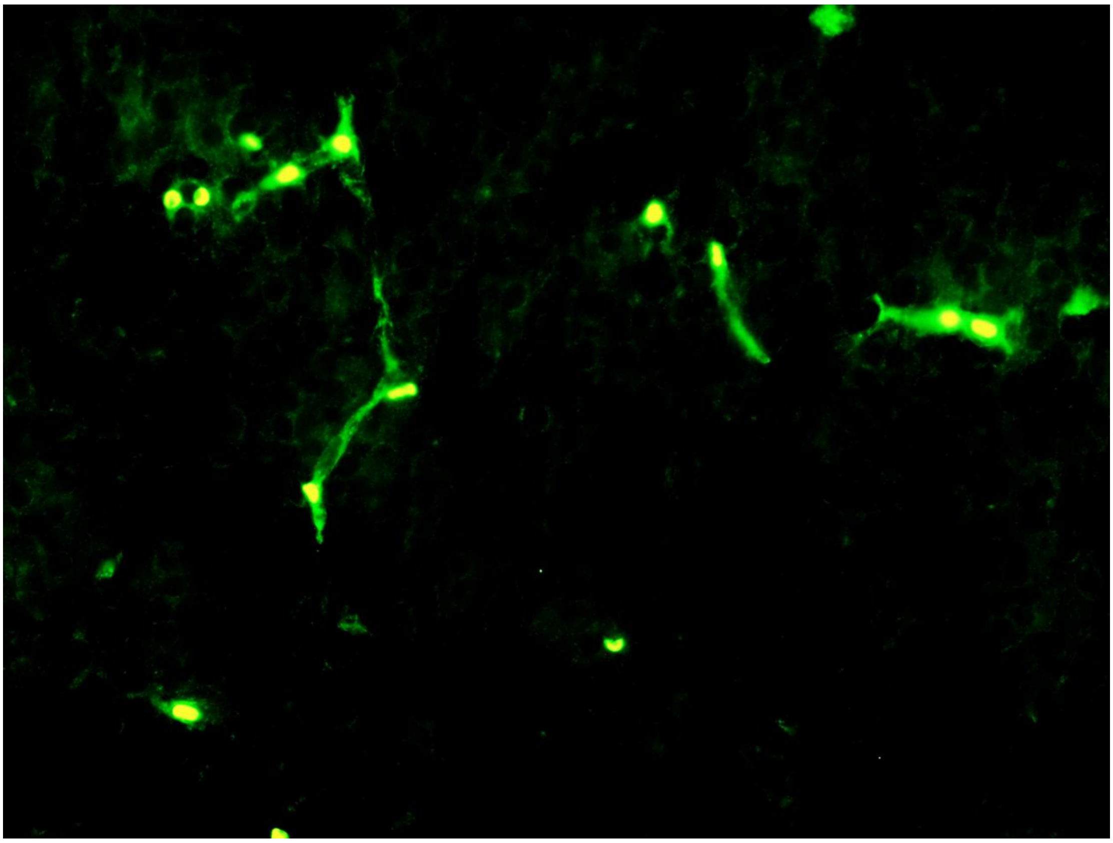

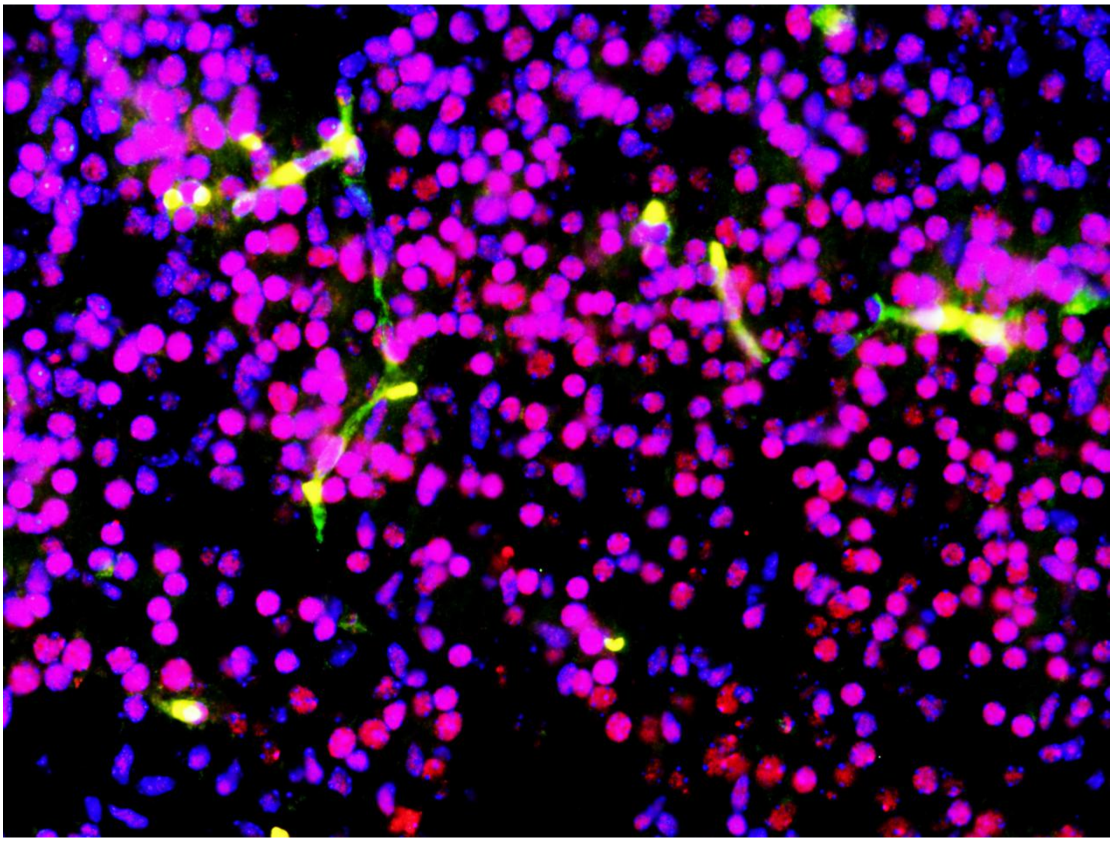

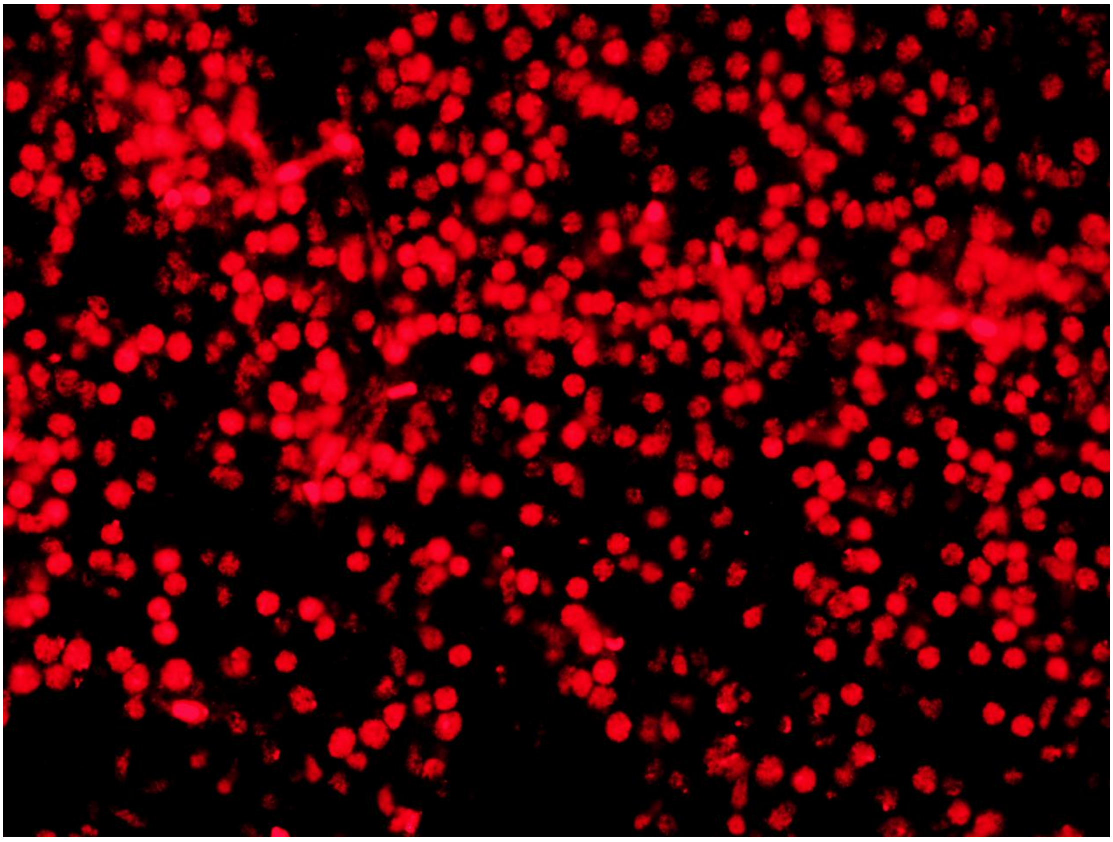

Hippocampus

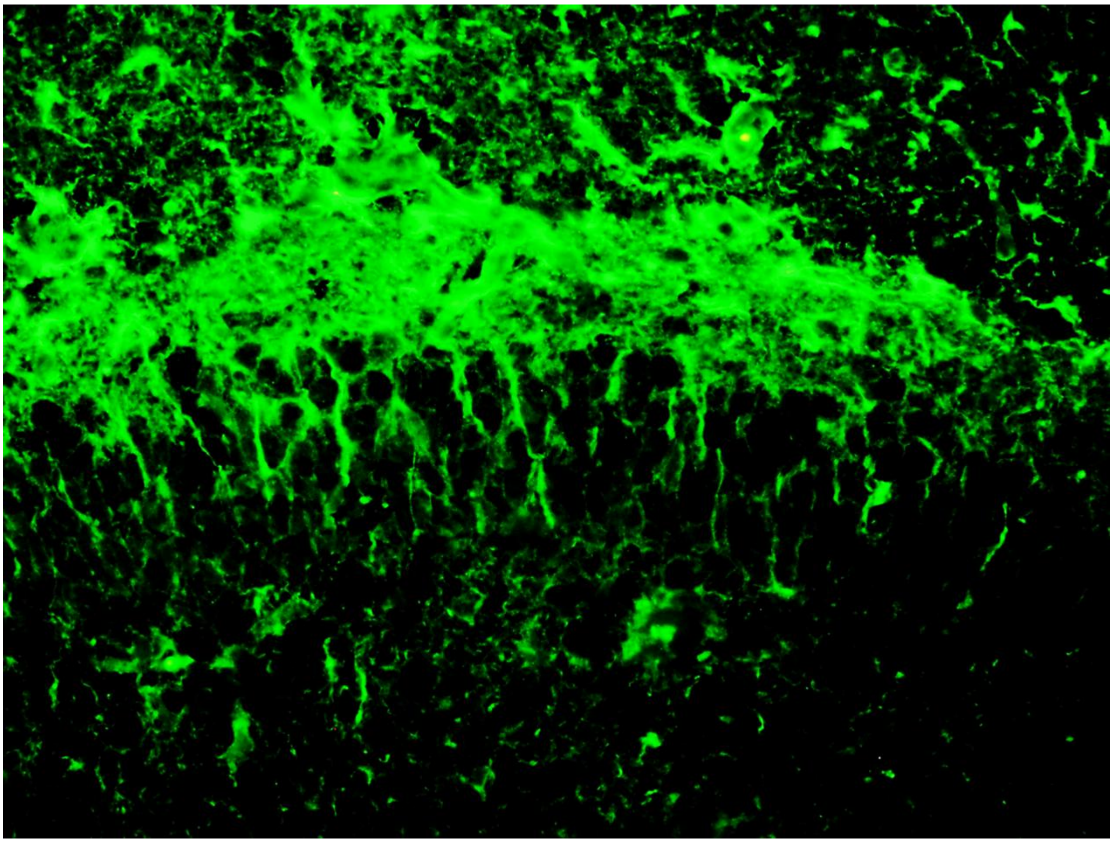

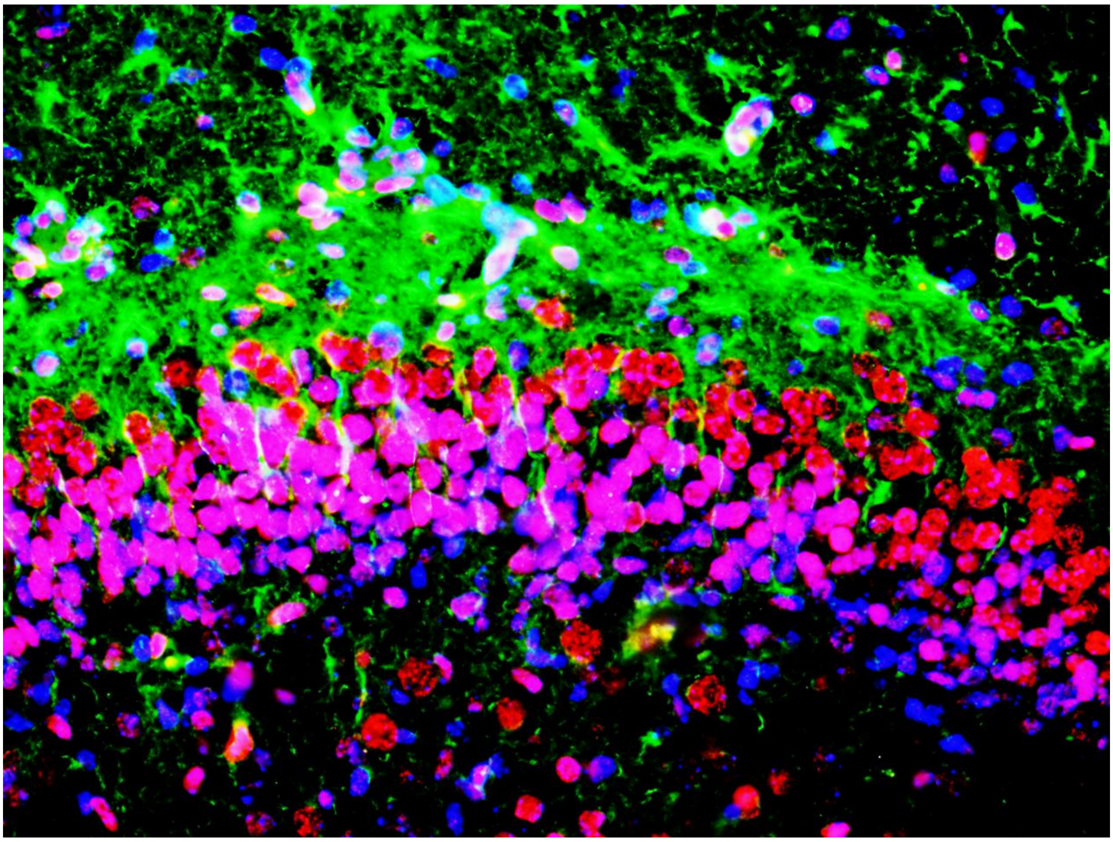

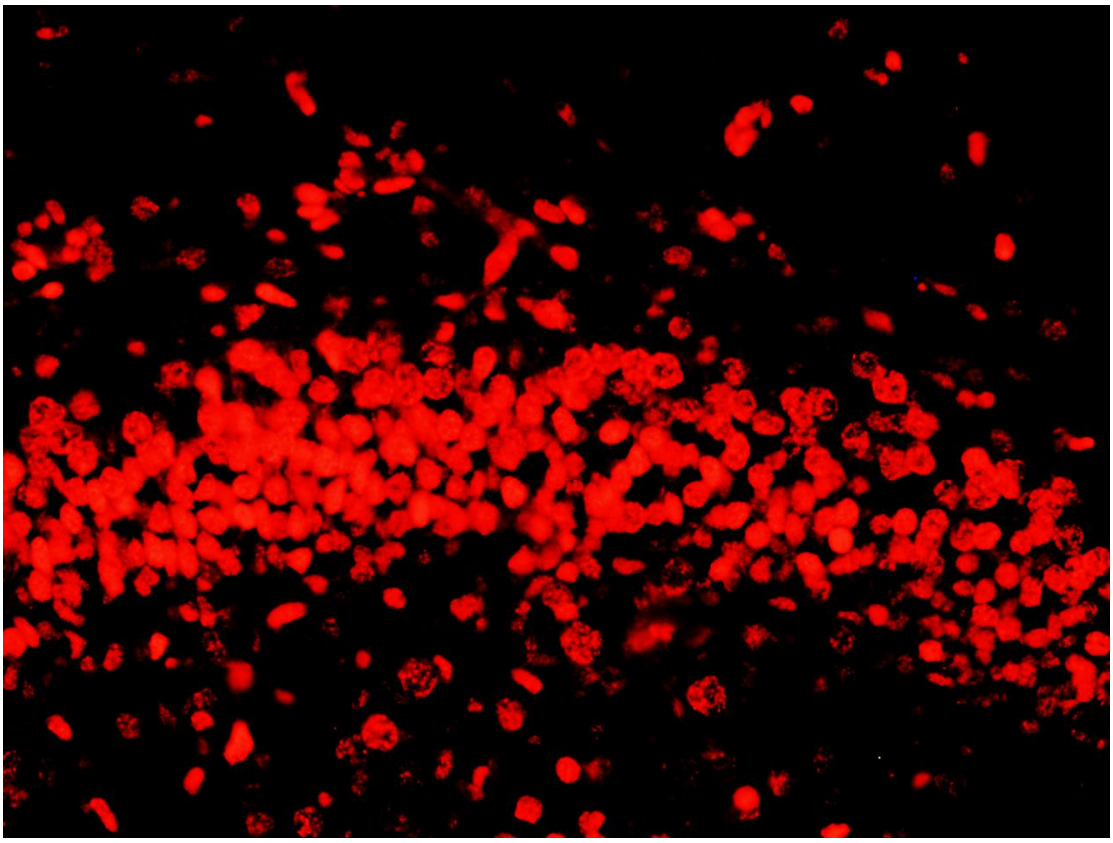

Figure6G **PIA+FA**

Cortex

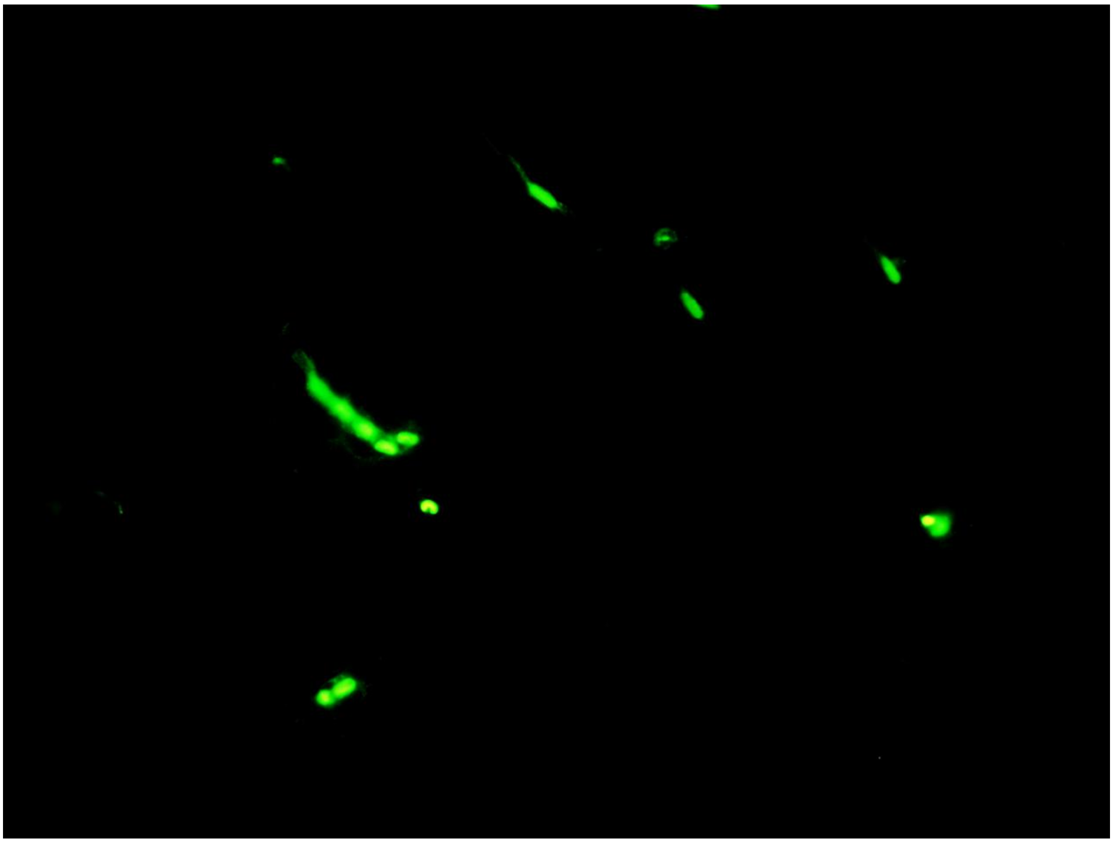

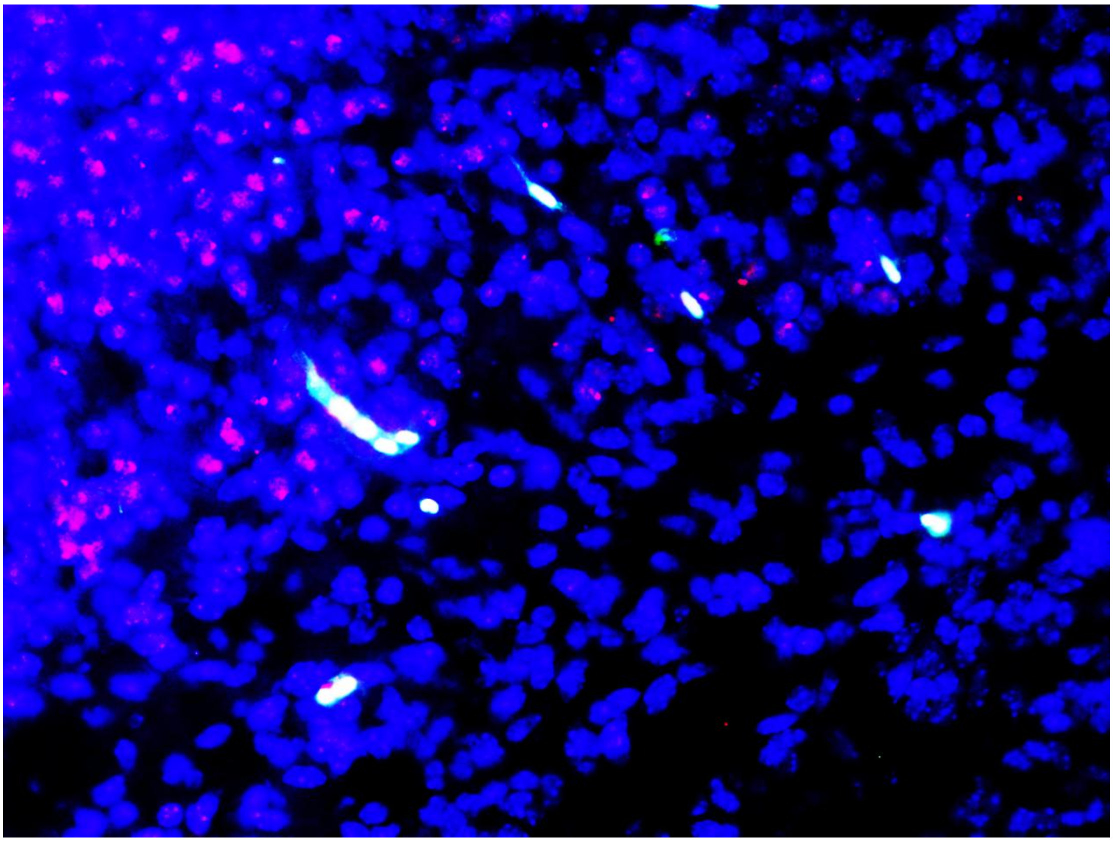

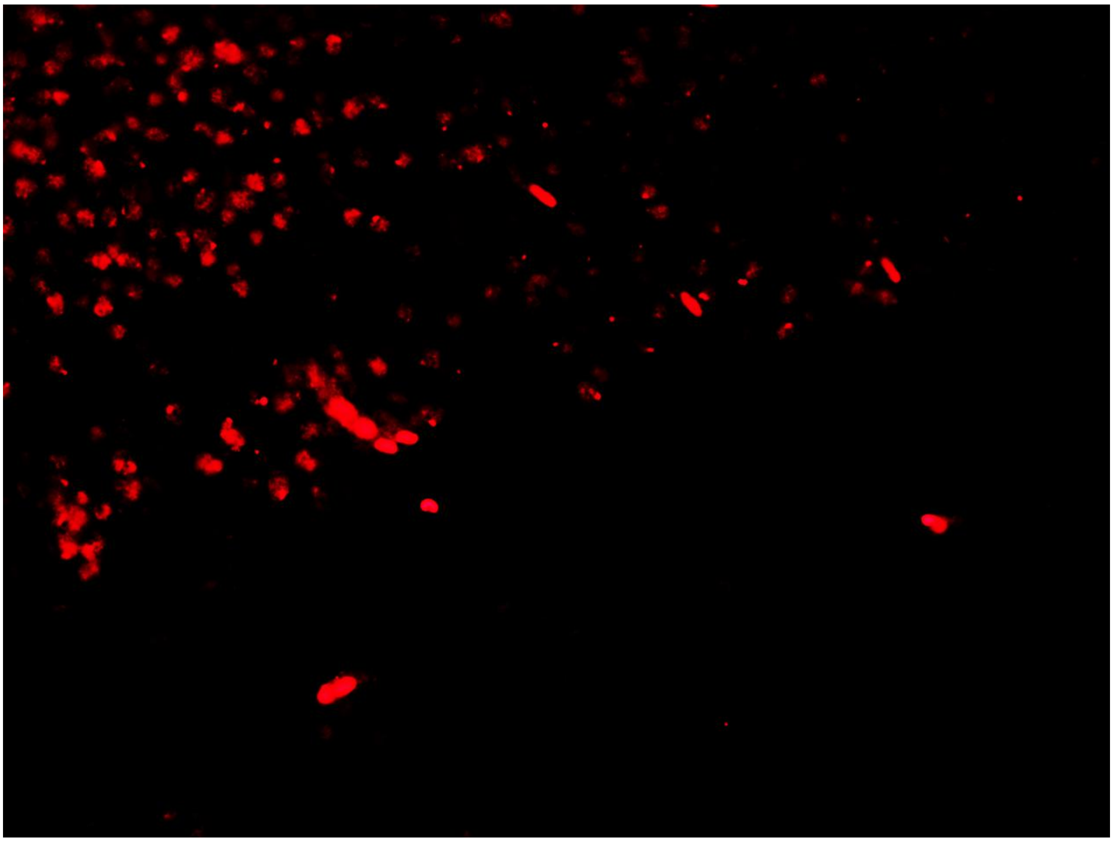

Hippocampus

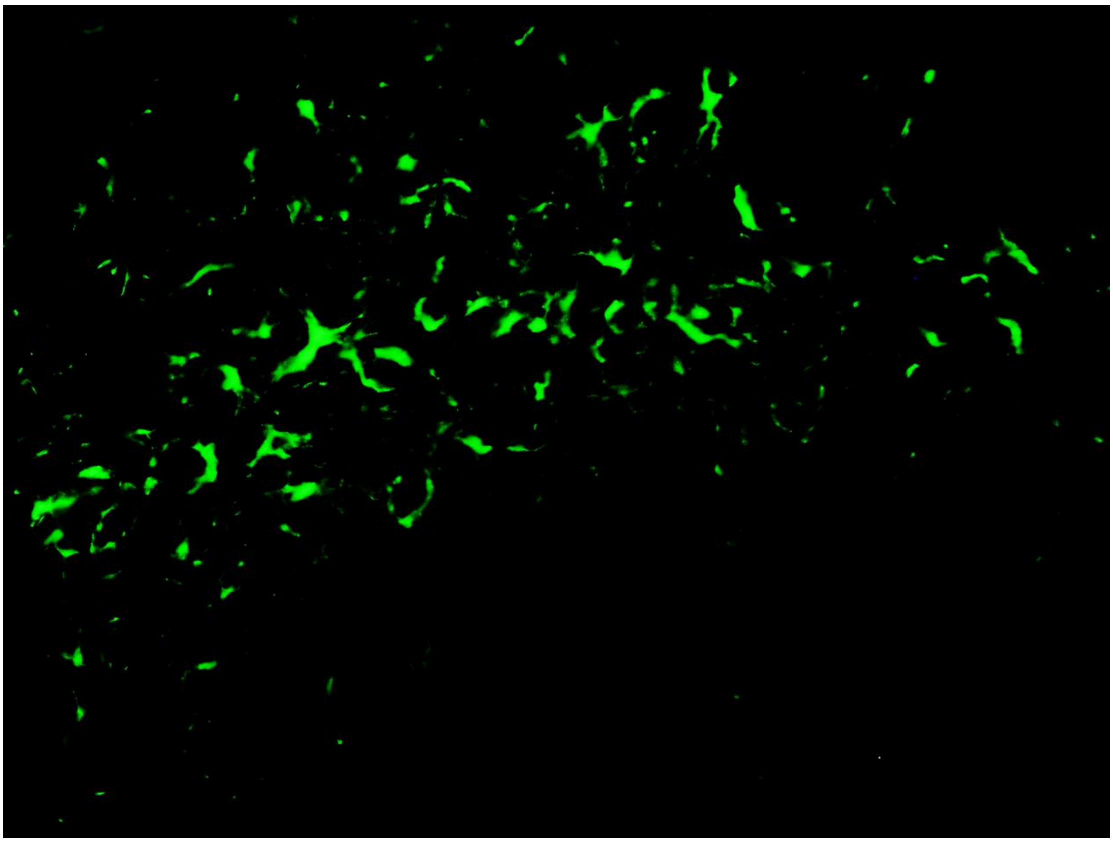

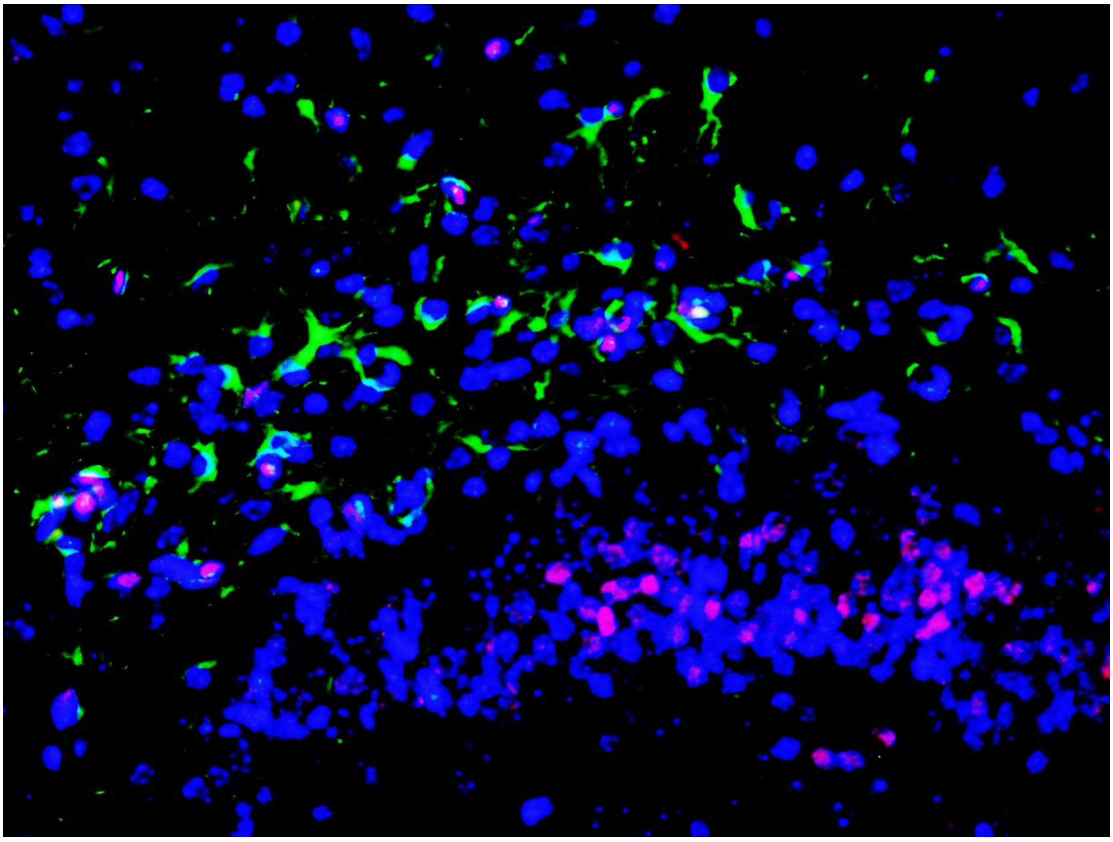

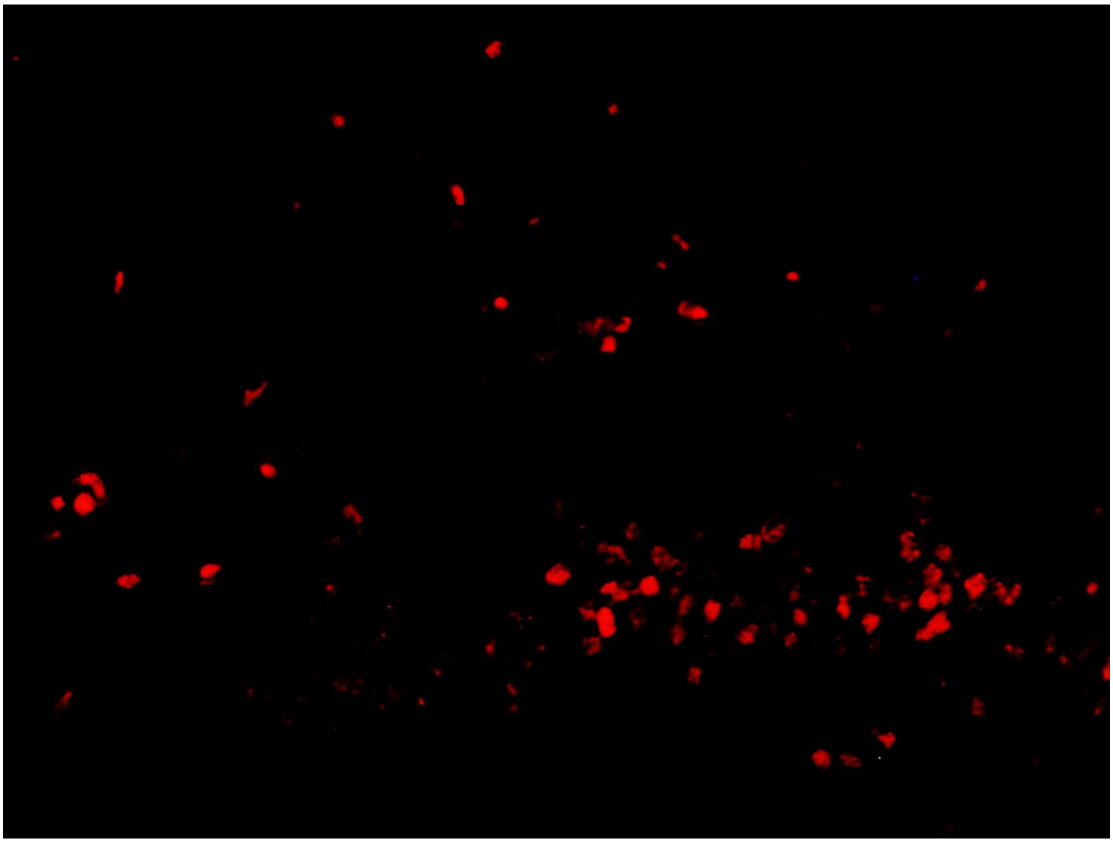

Figure6H **Control**

Cortex

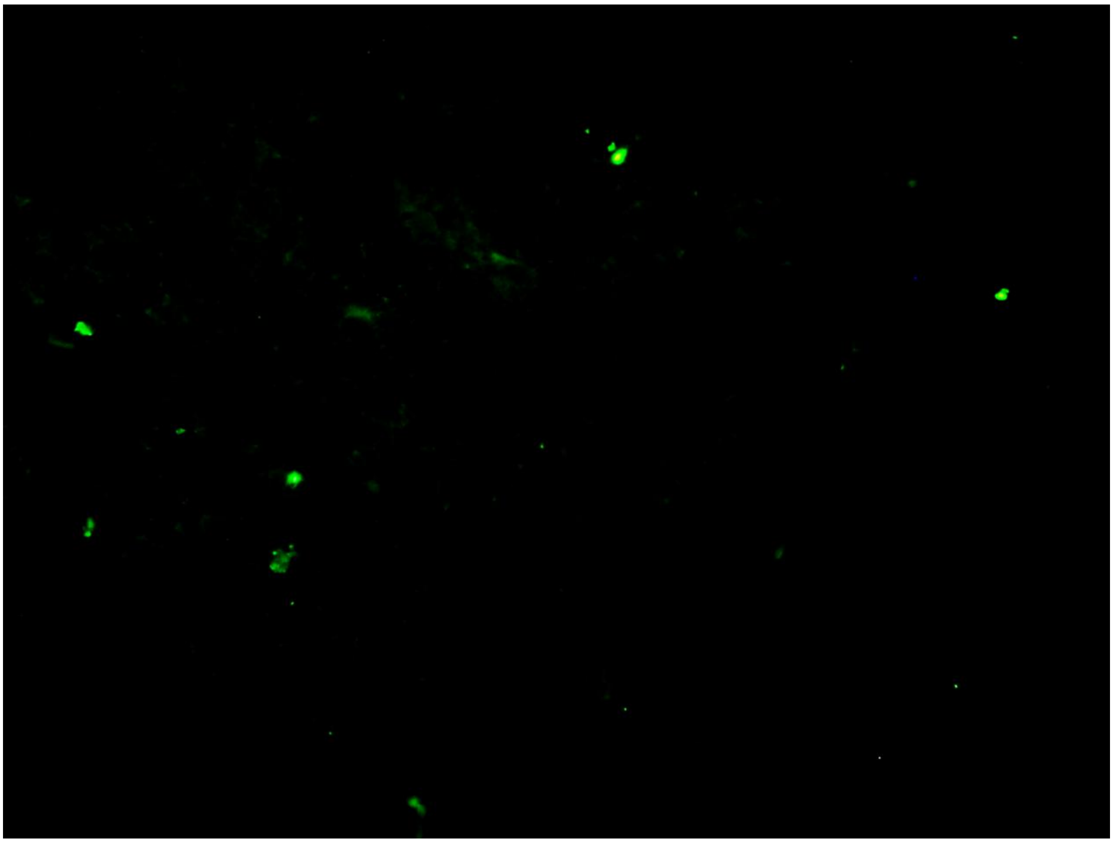

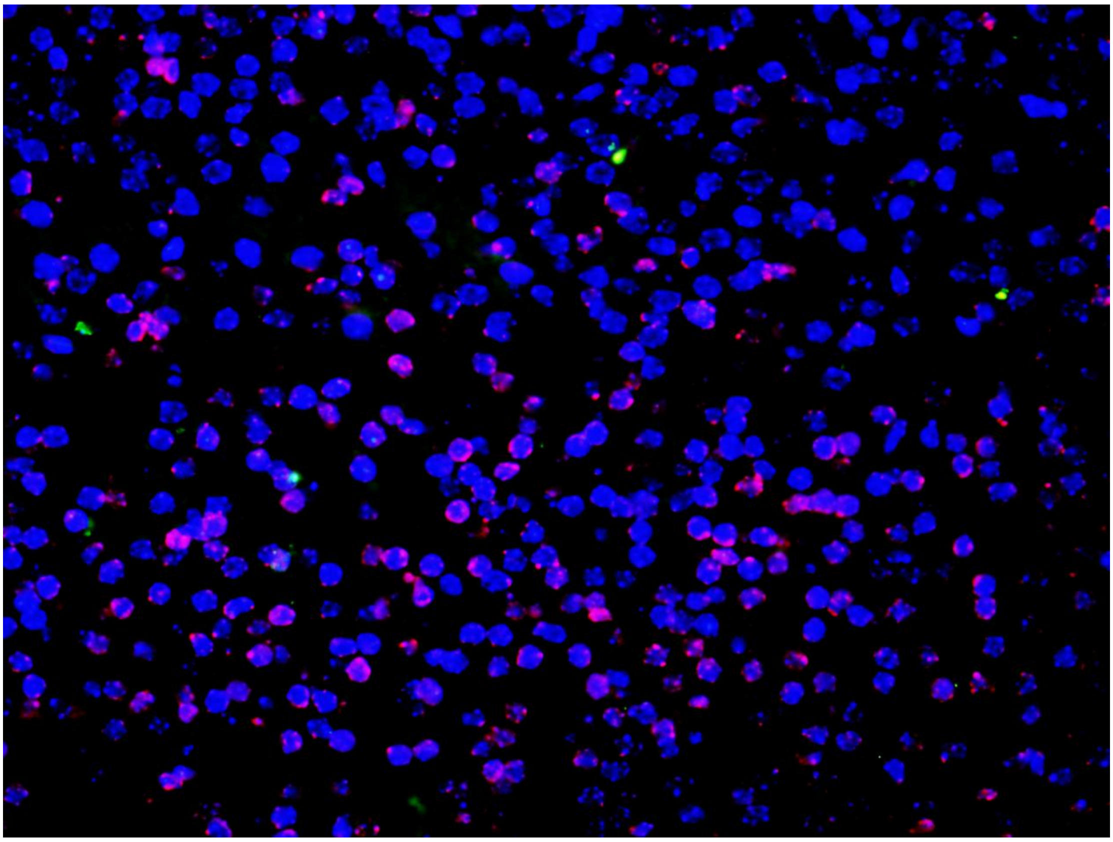

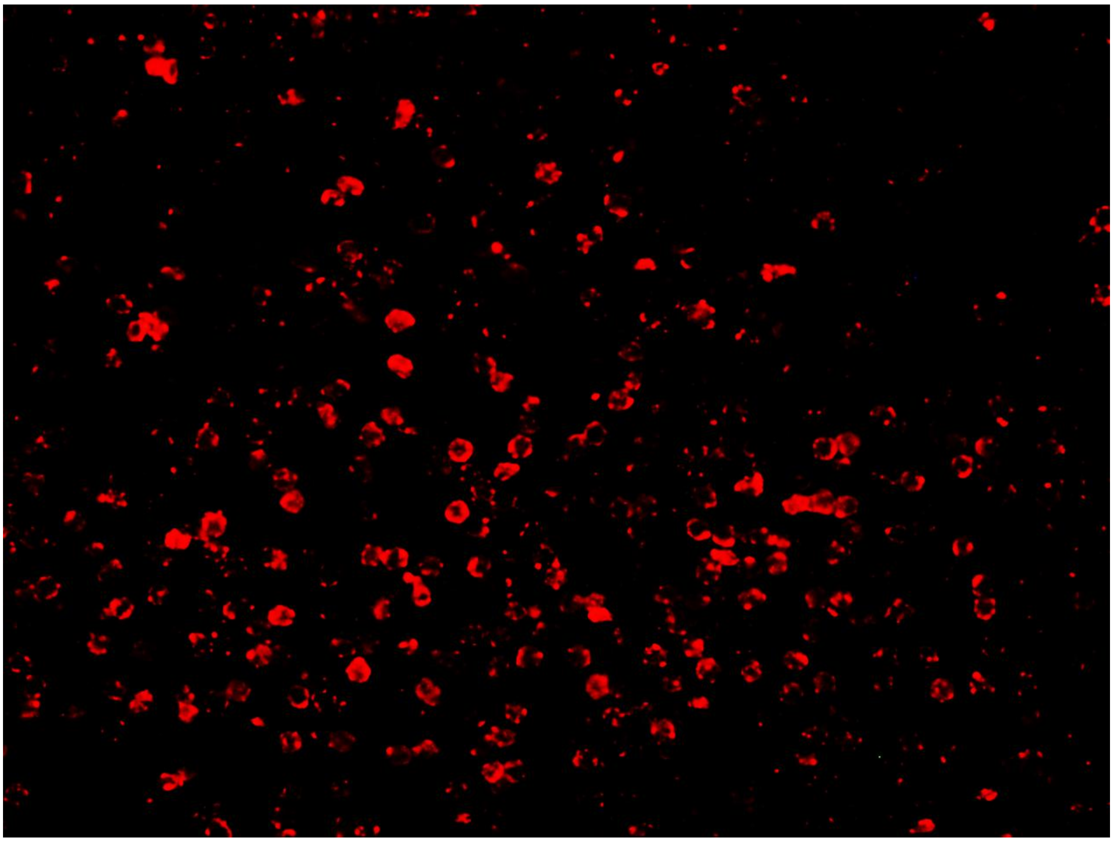

Hippocampus

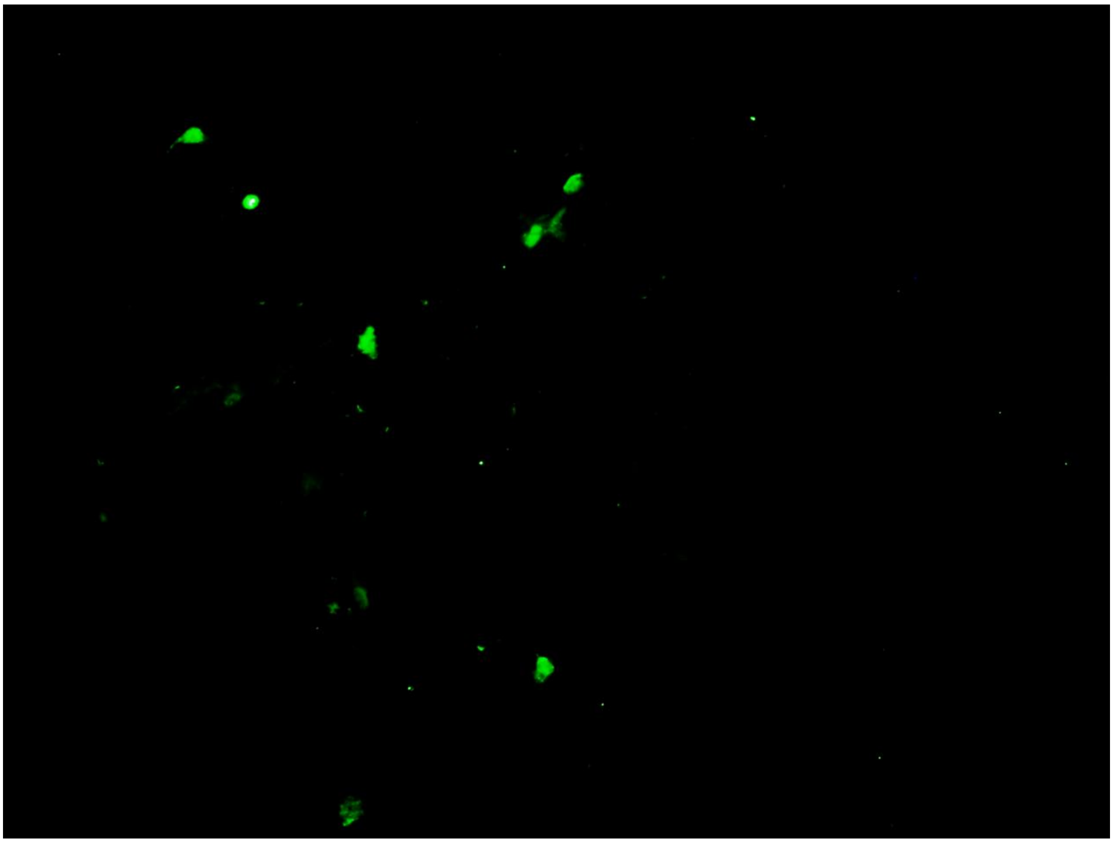

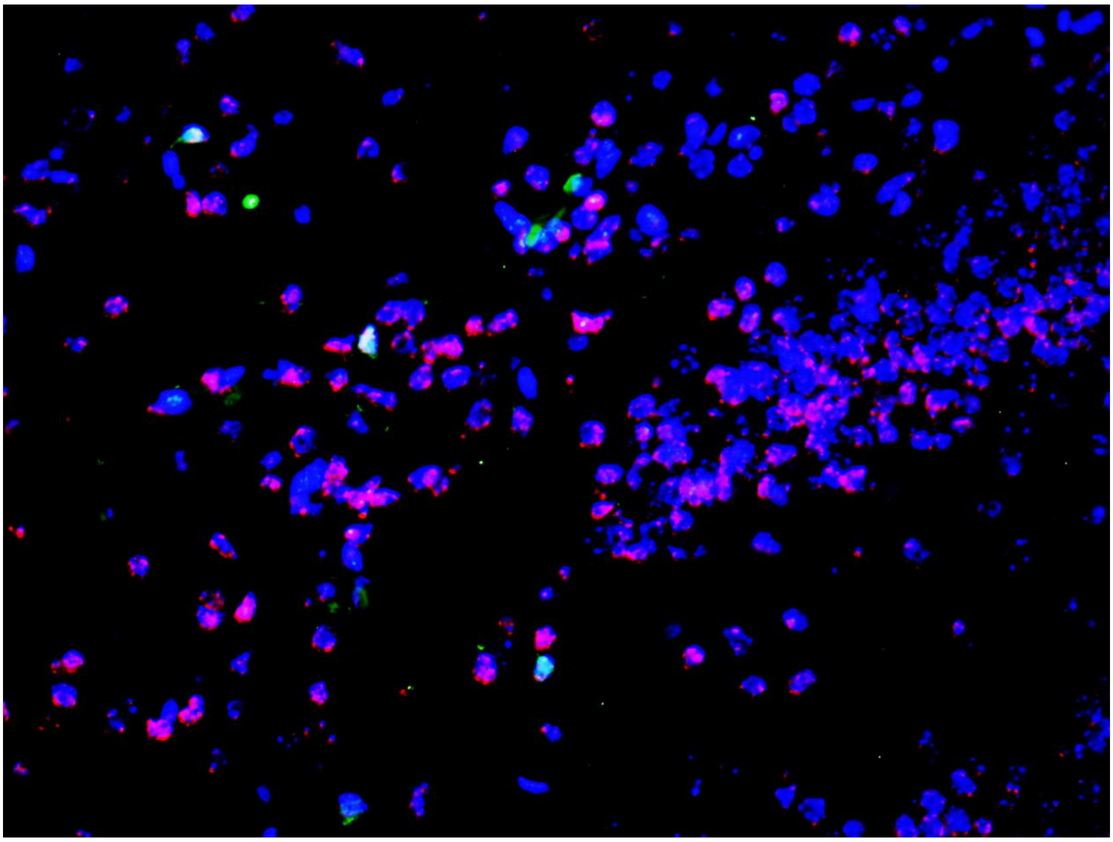

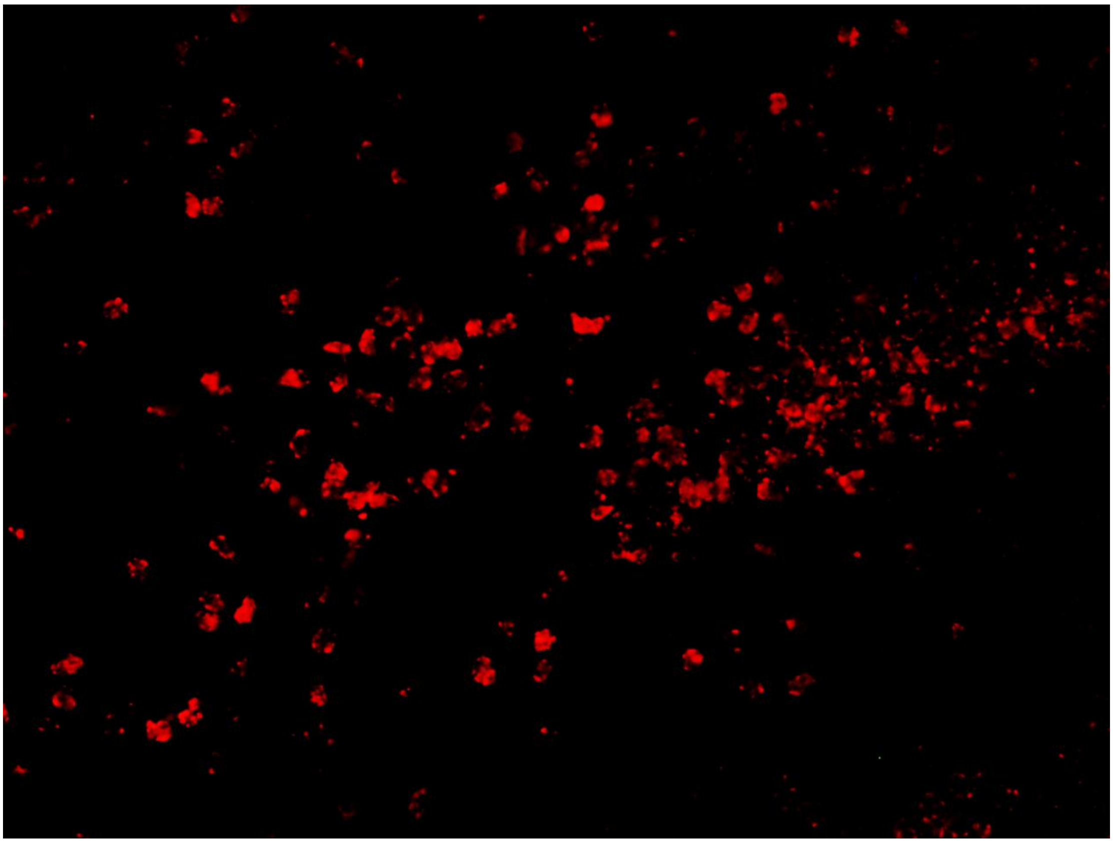

Figure6H **PIA**

Cortex

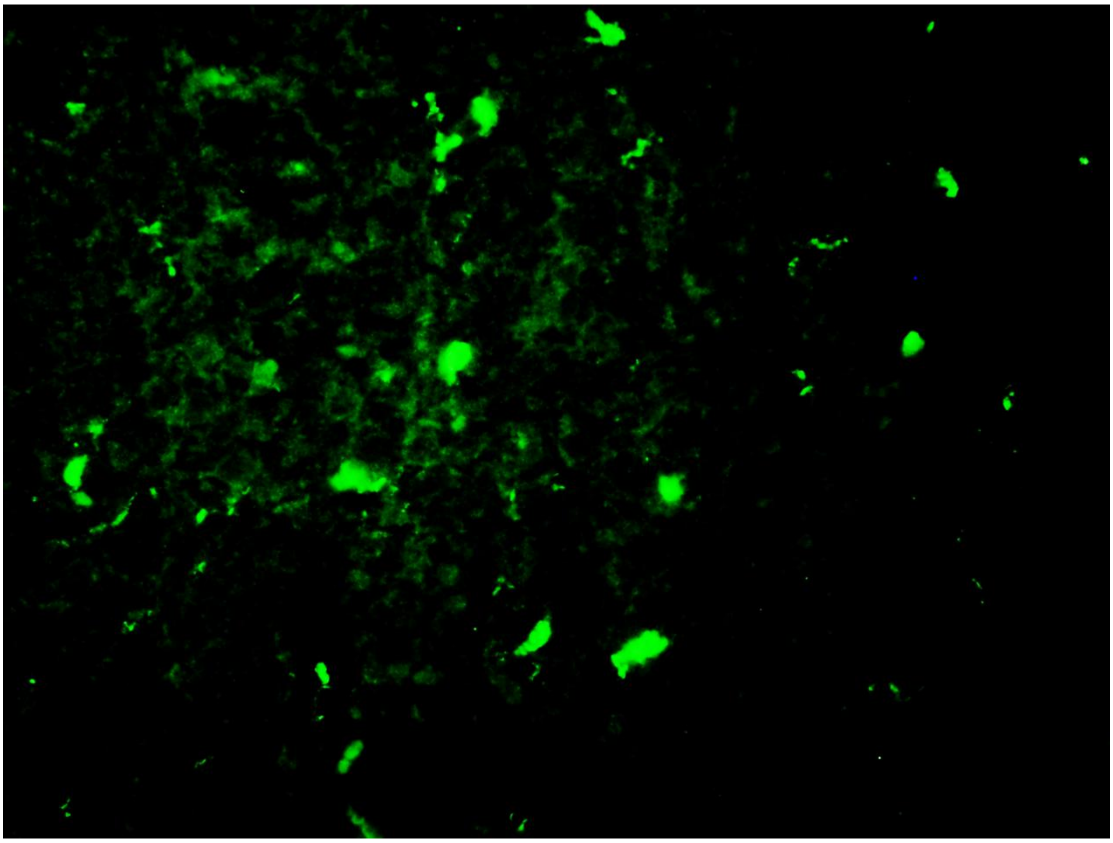

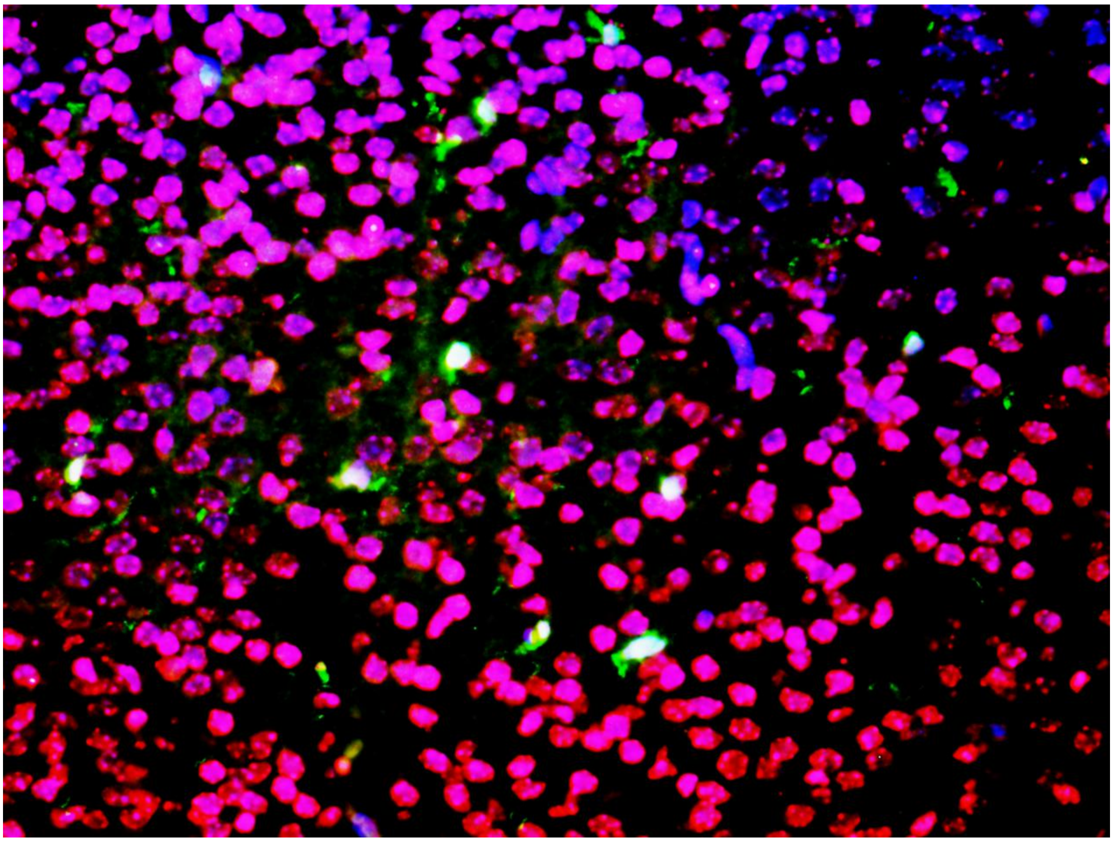

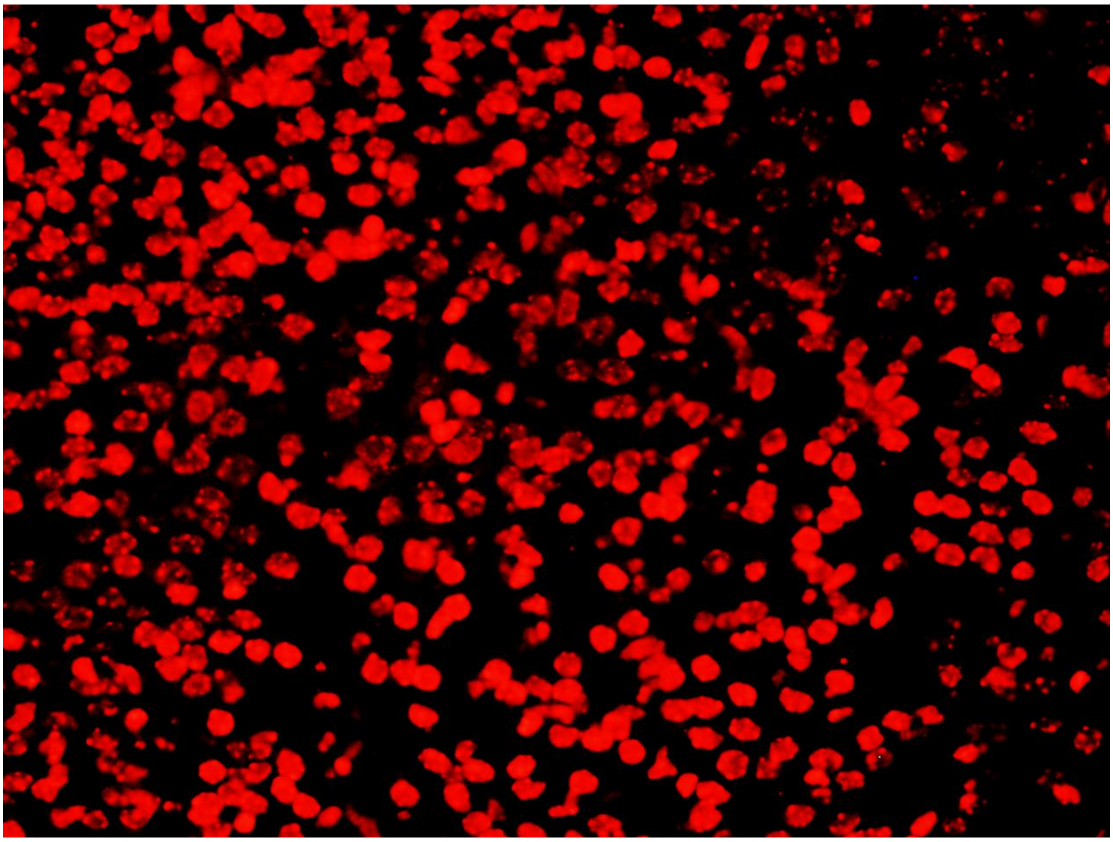

Hippocampus

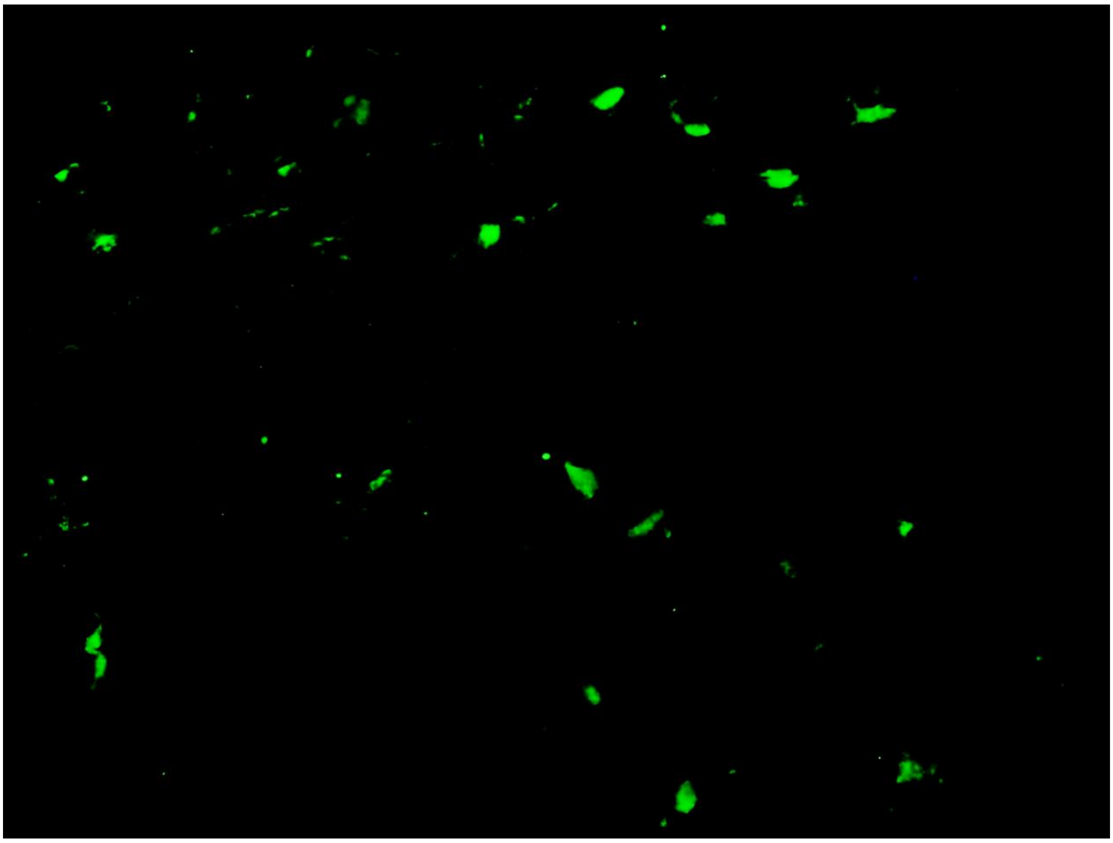

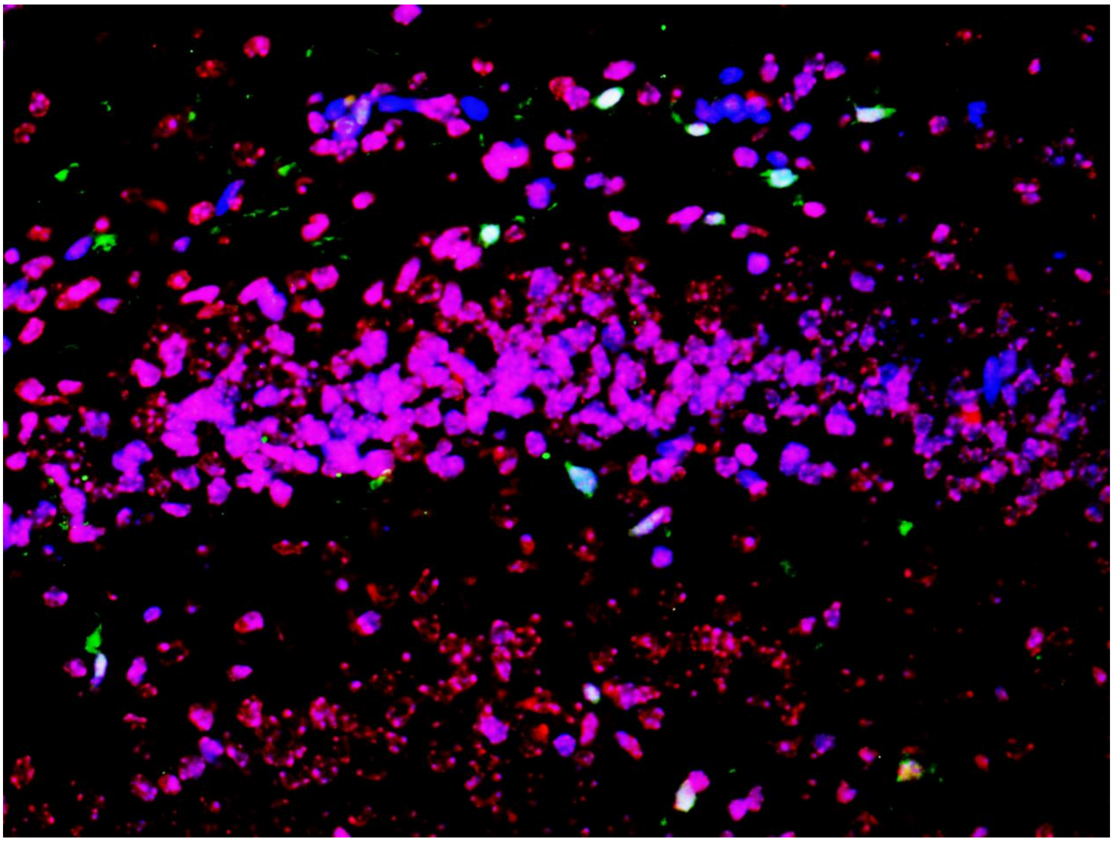

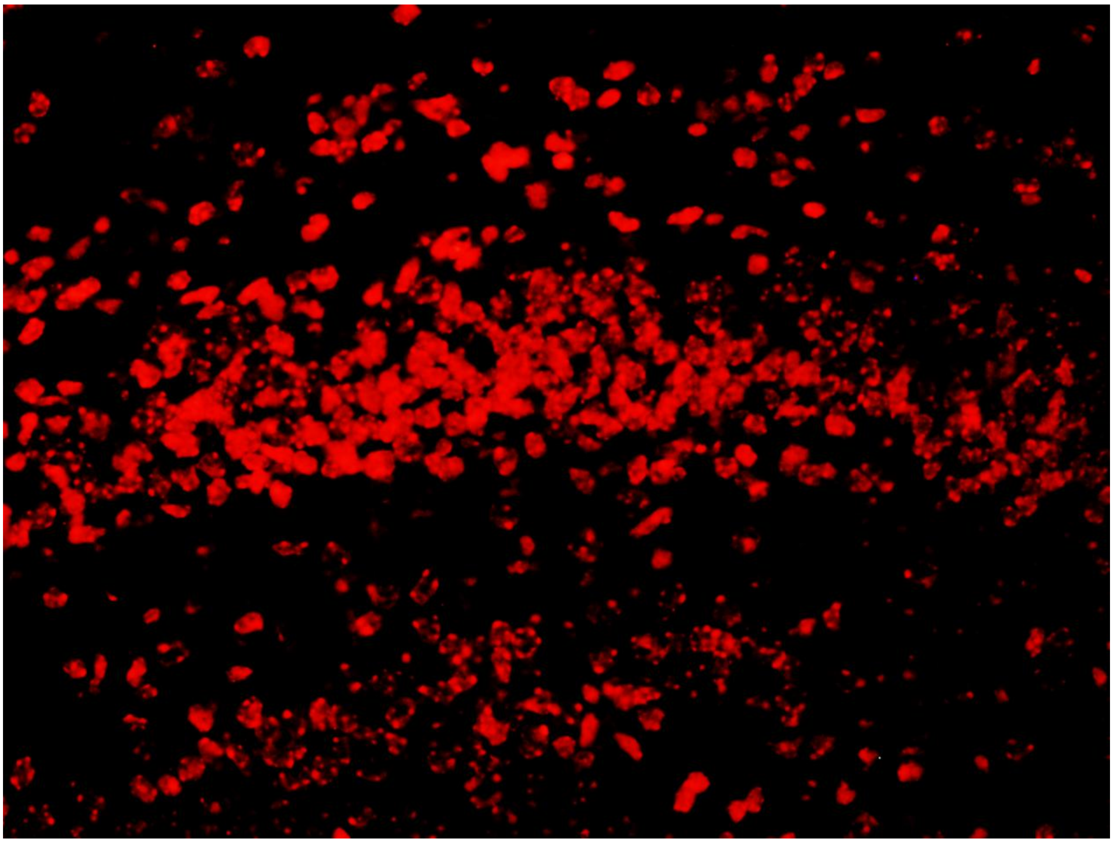

Figure6H **PIA+FA**

Cortex

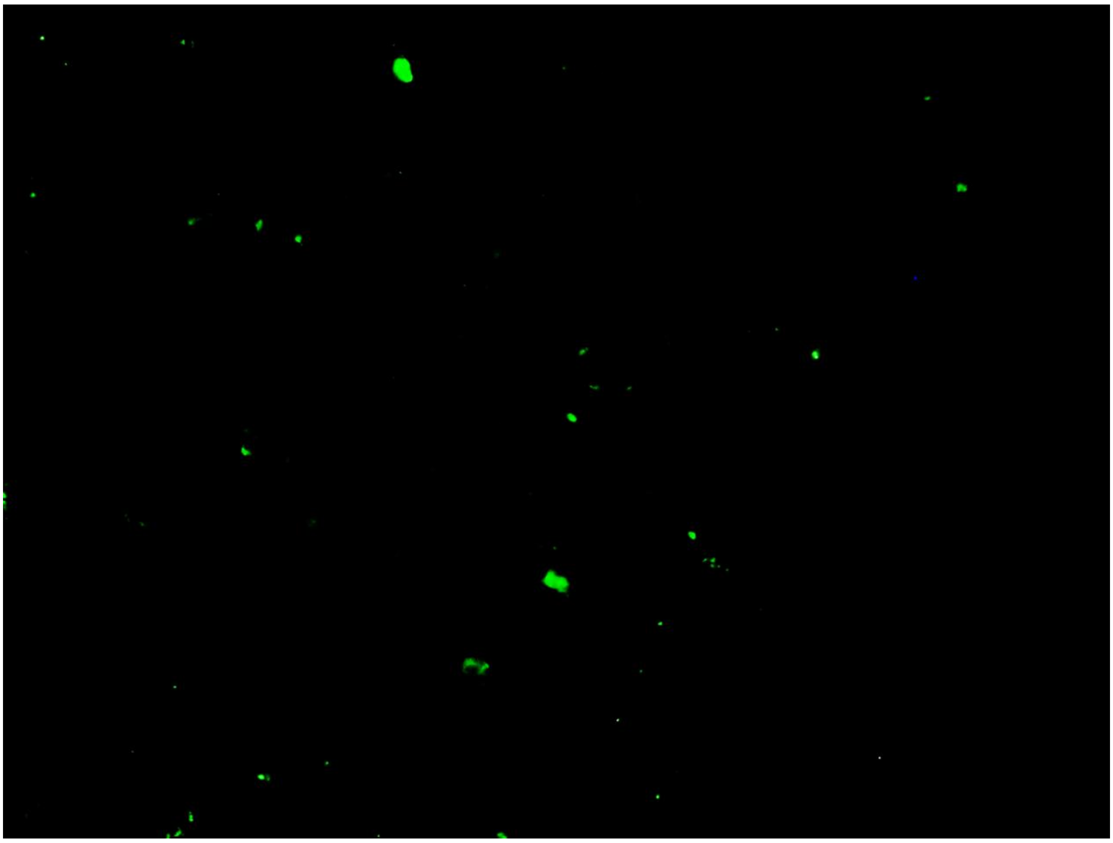

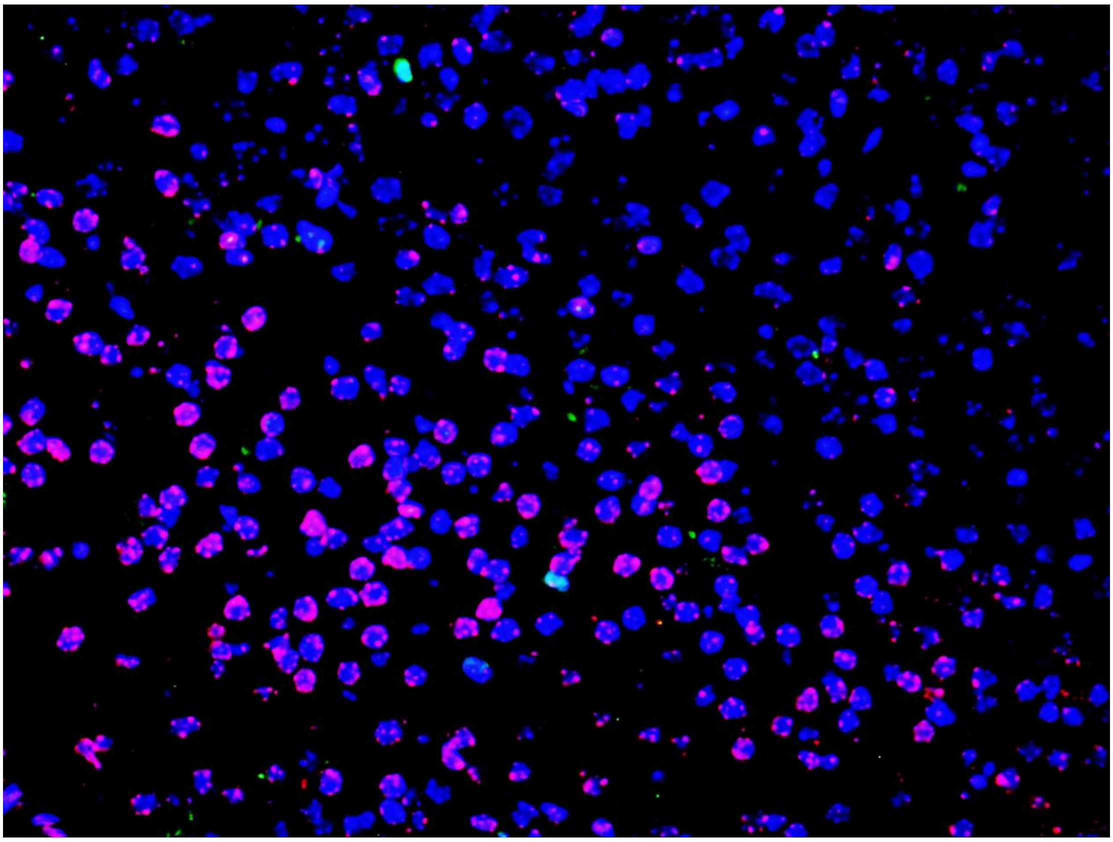

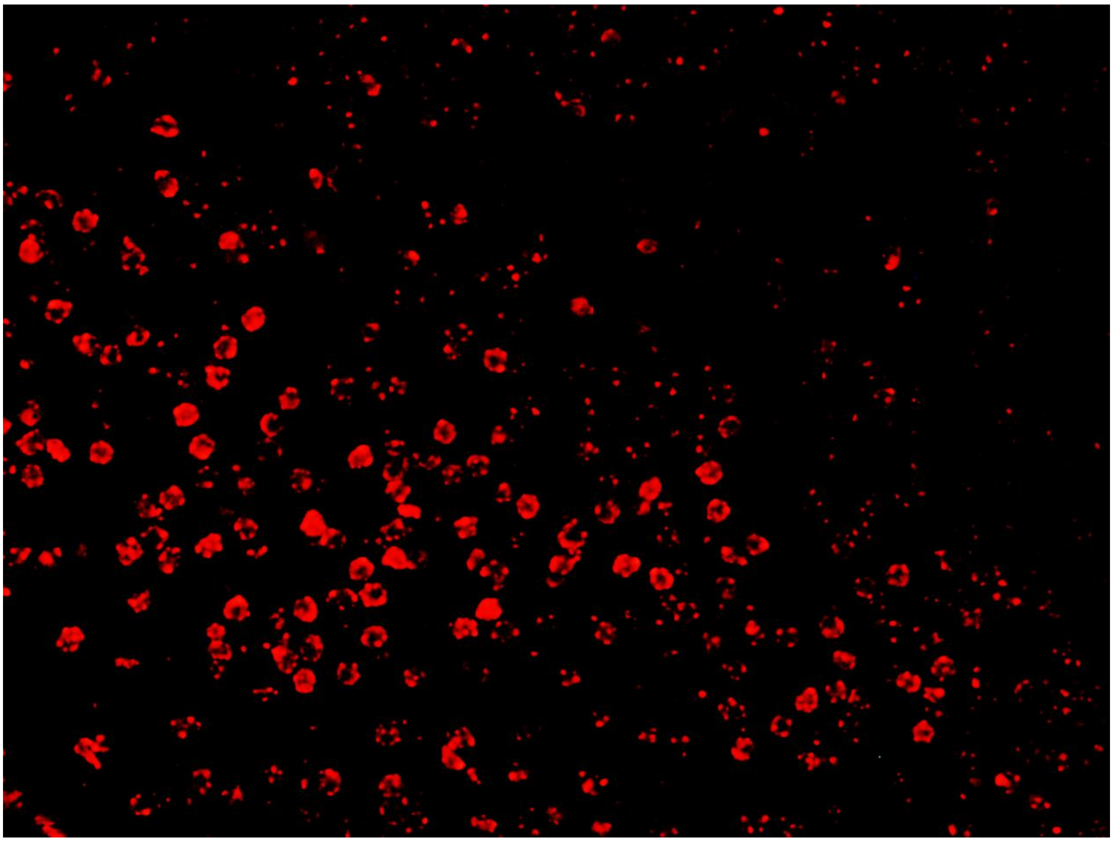

Hippocampus

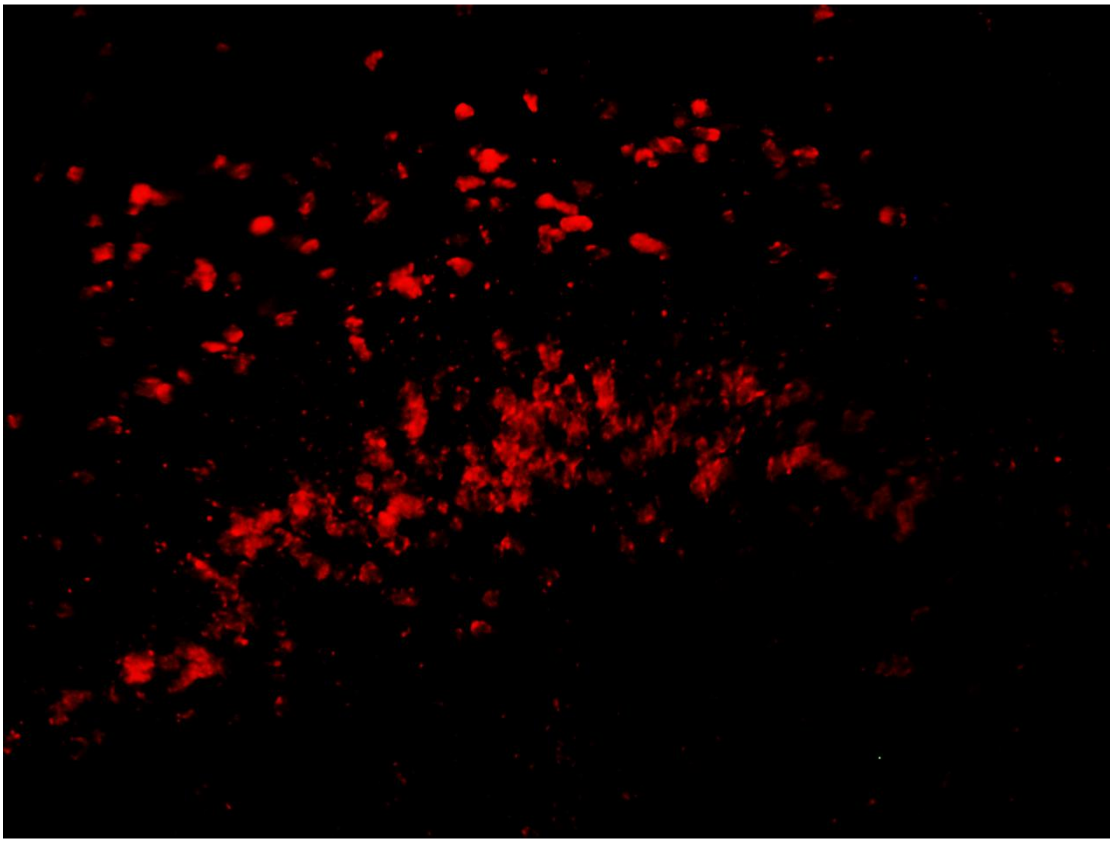

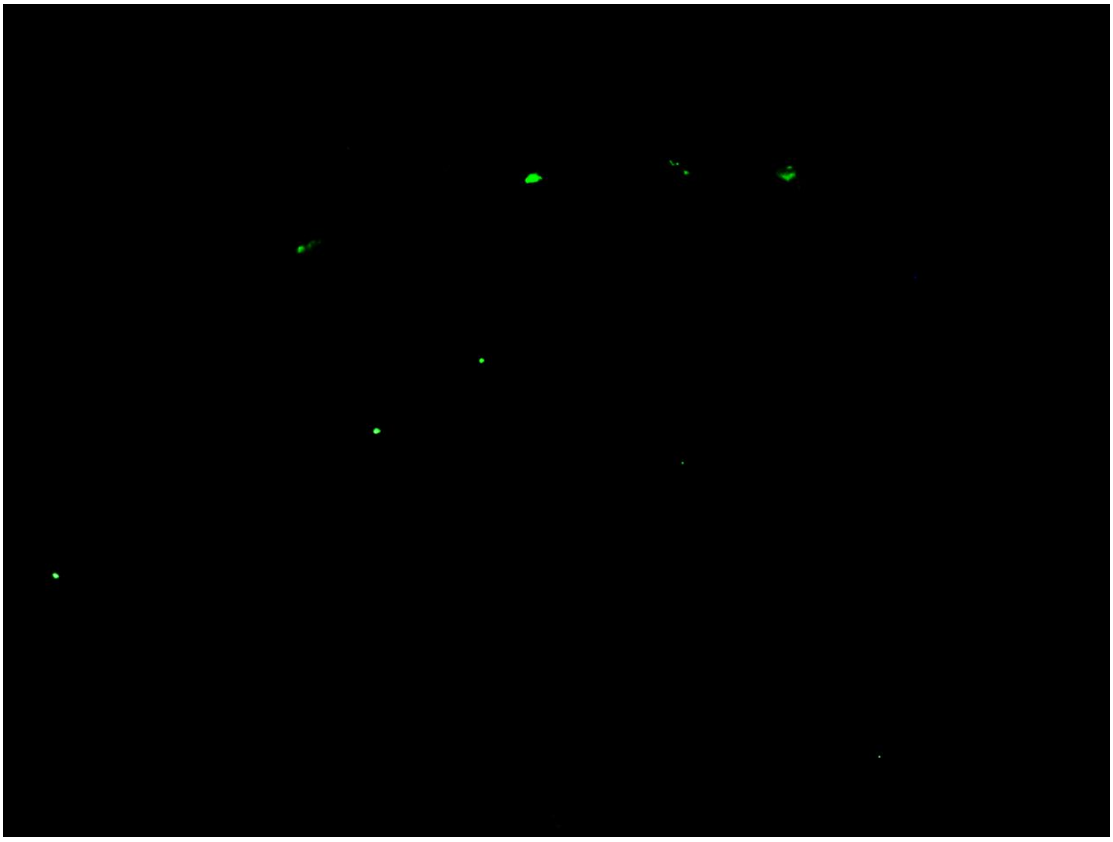

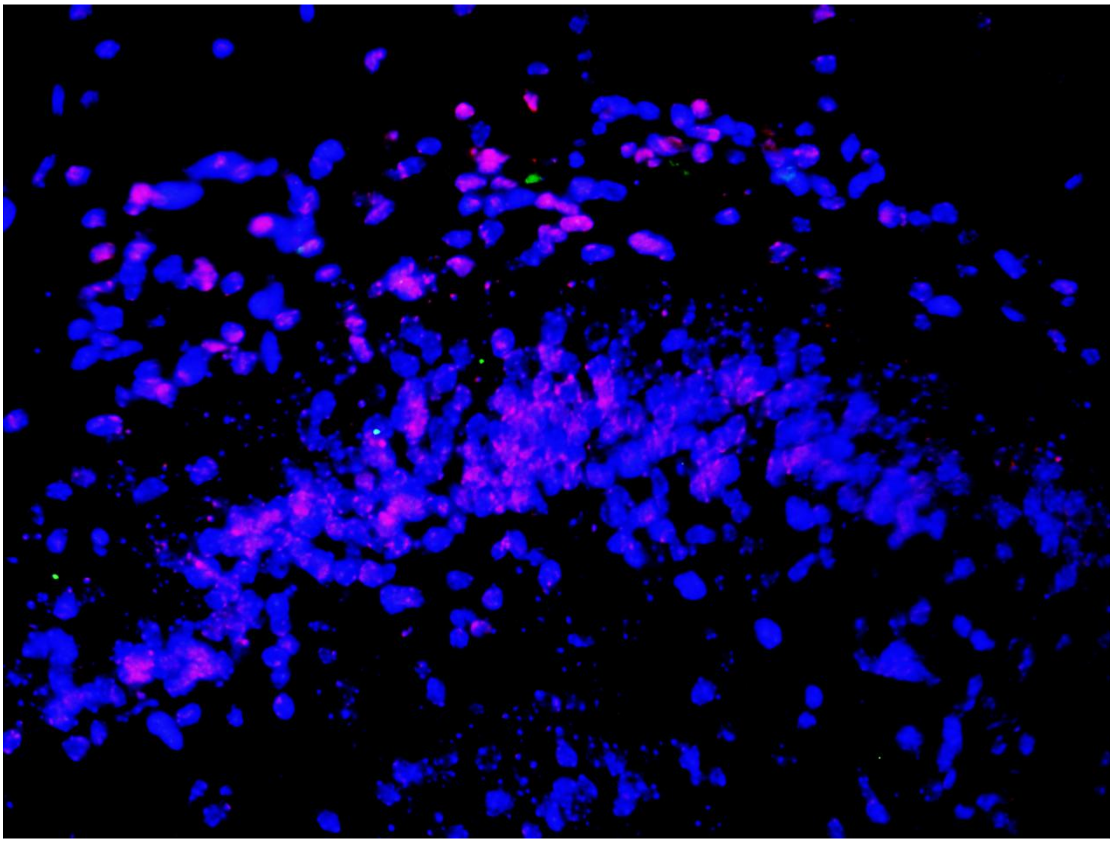

Supplementary figure1B

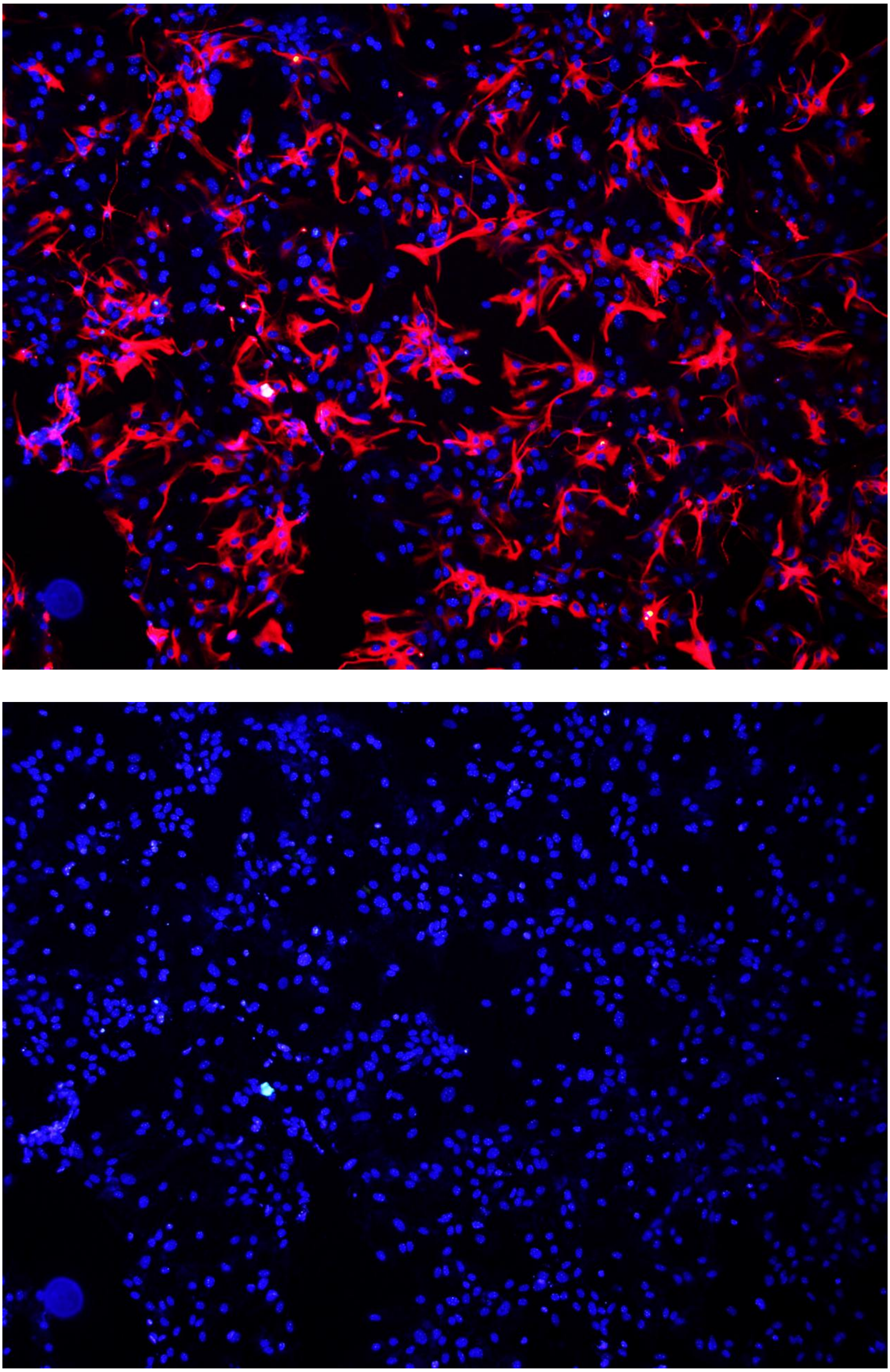

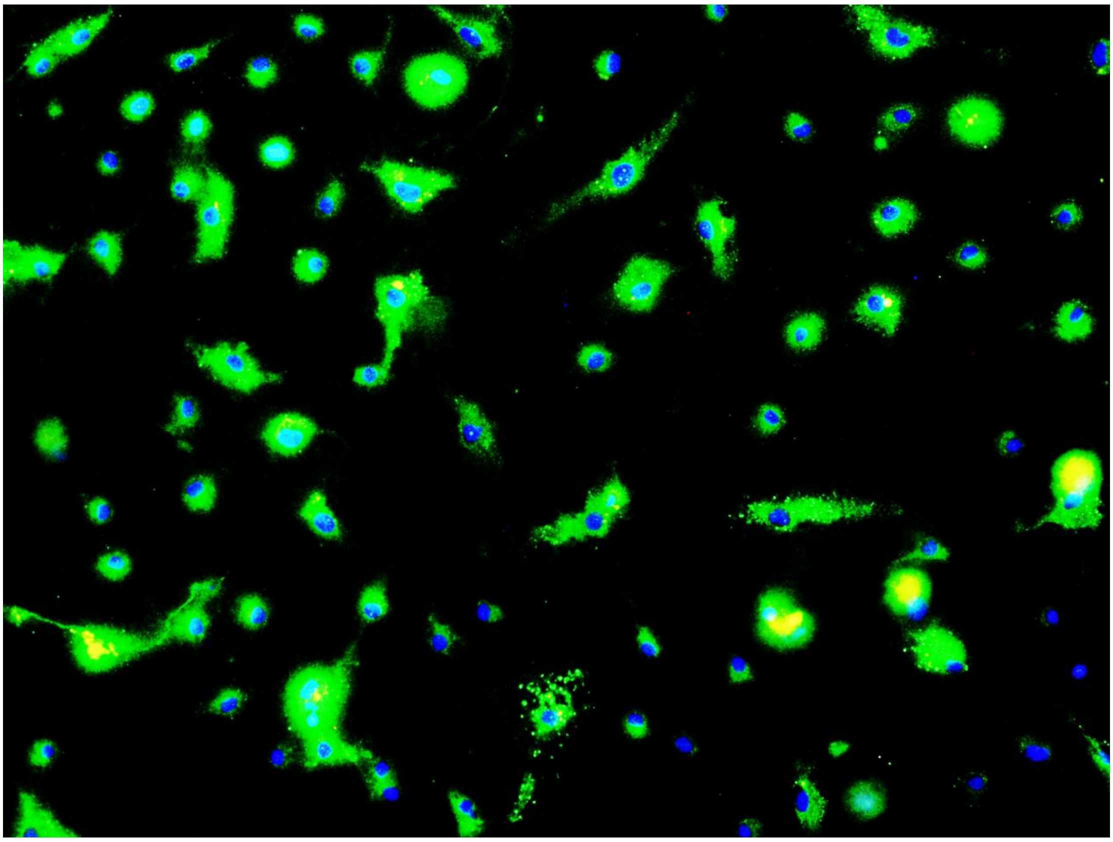

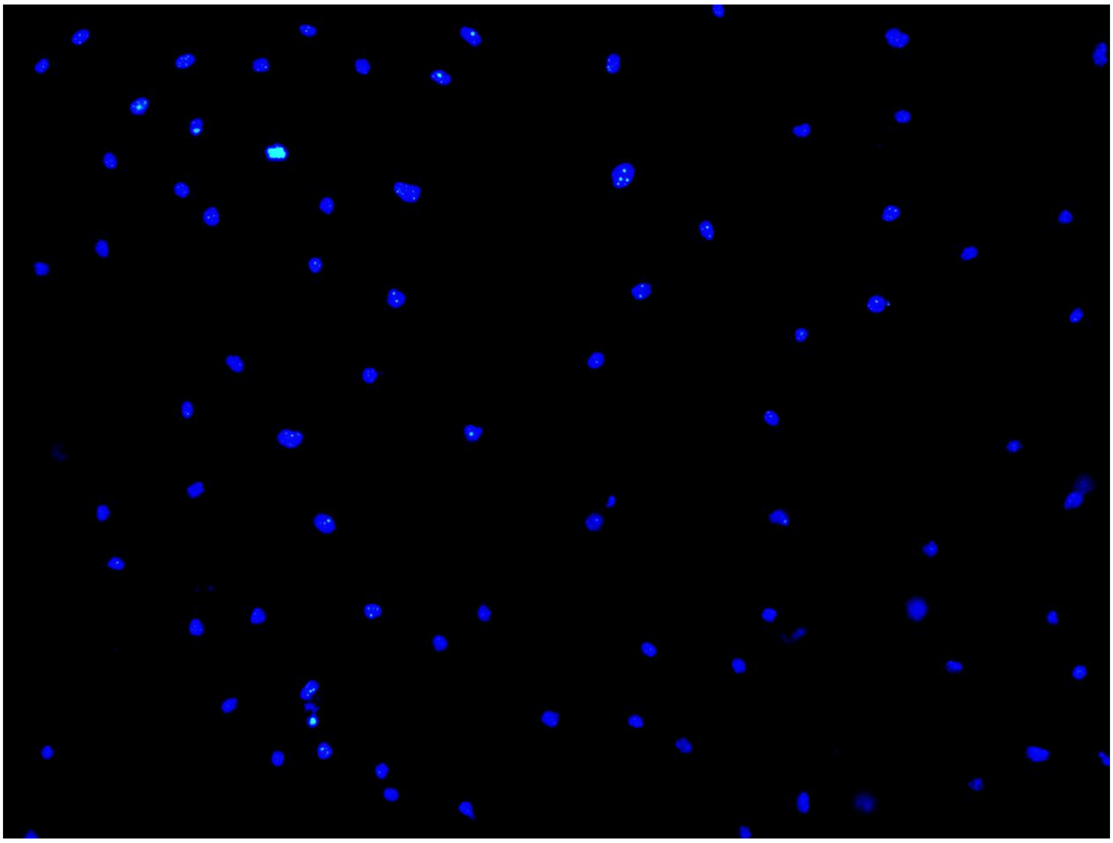

Supplement: Supplementary file 1 [file DataSheet1.PDF]
